# A Detailed History of Intron-rich Eukaryotic Ancestors Inferred from a Global Survey of 100 Complete Genomes

**DOI:** 10.1371/journal.pcbi.1002150

**Published:** 2011-09-15

**Authors:** Miklos Csuros, Igor B. Rogozin, Eugene V. Koonin

**Affiliations:** 1 Department of Computer Science and Operations Research, Université de Montréal, Montréal, Québec, Canada; 2 National Center for Biotechnology Information, National Library of Medicine, National Institutes of Health, Bethesda, Maryland, United States of America; University of Oxford, United Kingdom

## Abstract

Protein-coding genes in eukaryotes are interrupted by introns, but intron densities widely differ between eukaryotic lineages. Vertebrates, some invertebrates and green plants have intron-rich genes, with 6–7 introns per kilobase of coding sequence, whereas most of the other eukaryotes have intron-poor genes. We reconstructed the history of intron gain and loss using a probabilistic Markov model (Markov Chain Monte Carlo, MCMC) on 245 orthologous genes from 99 genomes representing the three of the five supergroups of eukaryotes for which multiple genome sequences are available. Intron-rich ancestors are confidently reconstructed for each major group, with 53 to 74% of the human intron density inferred with 95% confidence for the Last Eukaryotic Common Ancestor (LECA). The results of the MCMC reconstruction are compared with the reconstructions obtained using Maximum Likelihood (ML) and Dollo parsimony methods. An excellent agreement between the MCMC and ML inferences is demonstrated whereas Dollo parsimony introduces a noticeable bias in the estimations, typically yielding lower ancestral intron densities than MCMC and ML. Evolution of eukaryotic genes was dominated by intron loss, with substantial gain only at the bases of several major branches including plants and animals. The highest intron density, 120 to 130% of the human value, is inferred for the last common ancestor of animals. The reconstruction shows that the entire line of descent from LECA to mammals was intron-rich, a state conducive to the evolution of alternative splicing.

## Author Summary

In eukaryotes, protein-coding genes are interrupted by non-coding introns. The intron densities widely differ, from 6–7 introns per kilobase of coding sequence in vertebrates, some invertebrates and plants, to only a few introns across the entire genome in many unicellular forms. We applied a robust statistical methodology, Markov Chain Monte Carlo, to reconstruct the history of intron gain and loss throughout the evolution of eukaryotes using a set of 245 homologous genes from 99 genomes that represent the diversity of eukaryotes. Intron-rich ancestors were confidently inferred for each major eukaryotic group including 53% to 74% of the human intron density for the last eukaryotic common ancestor, and 120% to 130% of the human value for the last common ancestor of animals. Evolution of eukaryotic genes involved primarily intron loss, with substantial gain only at the bases of several major branches including plants and animals. Thus, the common ancestor of all extant eukaryotes was a complex organism with a gene architecture resembling those in multicellular organisms. The line of descent from the last common ancestor to mammals was an uninterrupted intron-rich state that, given the error-prone splicing in intron-rich organisms, was conducive to the elaboration of functional alternative splicing.

## Introduction

Spliceosomal introns that interrupt most of the protein-coding genes and the concurrent splicing machinery that mediates intron excision and exon splicing are defining features of gene architecture and expression in eukaryotes [1], [2]. To date, eukaryote genomes including the compact genomes of parasitic protists, previously suspected to be intronless, have been shown to possess at least a few introns [3], [4], [5] and a (nearly) full complement of spliceosomal proteins [6]. However, eukaryotes dramatically differ in their intron densities, ranging from only a few introns per genome in many unicellular forms to over 8 introns per gene in vertebrates as well as some invertebrates like the sea anemone [7], [8].

Despite the ubiquity of introns in eukaryotic genomes, their biological status is poorly understood. To what extent introns are “junk DNA” as opposed to being functional parts of the genome, remains an open question and the answers are bound to be complicated and multifaceted. There are many reports on the contribution of introns to the regulation of gene expression [9], [10], and in vertebrates introns encode a variety of non-coding RNAs with established or predicted regulatory functions [11]. However, it remains unclear how general such functional roles of introns are. In addition to these specific functions, numerous introns are essential for alternative splicing which involves the great majority of genes in multicellular eukaryotes and is one of the principal mechanisms of proteome diversification [12], [13], [14].

Given that most unicellular eukaryotes are intron-poor whereas complex, multicellular organisms are intron-rich, it would seem intuitively plausible that introns accumulated in the course of evolution of eukaryotes. However, comparative analysis of the exon-intron structures of orthologous genes of plants and animals revealed a high level of intron position conservation, with the implication that the common ancestor of these organisms was relatively intron-rich [15], [16], [17], [18], [19]. Moreover, reconstructions of the evolution of gene architecture that were performed using maximum likelihood (ML) approaches suggested intron-rich ancestors for several major groups of eukaryotes [19], [20], [21] including even the Chromalveolata, a eukaryotic supergroup that consists entirely of unicellular organisms [22]. These results imply that evolution of eukaryotes involved at least as much intron loss as intron gain, and that intron loss was the main process in the majority of eukaryotic lineages whereas intron gain was only episodic [19], [21]. However, all these reconstructions provided relatively coarse resolution and involved substantial uncertainty with respect to the inference of intron density in deep ancestors, especially, the Last Eukaryotic Common Ancestor (LECA). The uncertainty was caused by the sparseness of the genomic data sets employed for the reconstruction and by the difficulty of assigning confidence intervals to inferences of ancestral state. As a result, depending on the features of the ML models employed and the data sets analyzed, some of the reconstructions yielded evolutionary scenarios with an excess of intron gain over intron loss [23].

Here we employ a probabilistic Monte Carlo model combined with a Markov Chain Monte Carlo (MCMC) method for the inference of ancestral states including robust estimation of confidence intervals to analyze a representative data set of 99 eukaryotic genomes which extensively covered the three supergroups of eukaryotes, Unikonta, Archaeaplastida (Plantae), and Chromalveolata, for which multiple genome sequences are available. The results clearly show that ancestral eukaryote forms were intron-rich, with LECA having a high intron density, on the order of two-thirds of the introns density in human genes. The subsequent evolution was heavily dominated by intron loss, with several episodes of massive intron gain associated with the emergence of some of the major eukaryote groups, in particular, animals.

## Results

**Figure 1:**
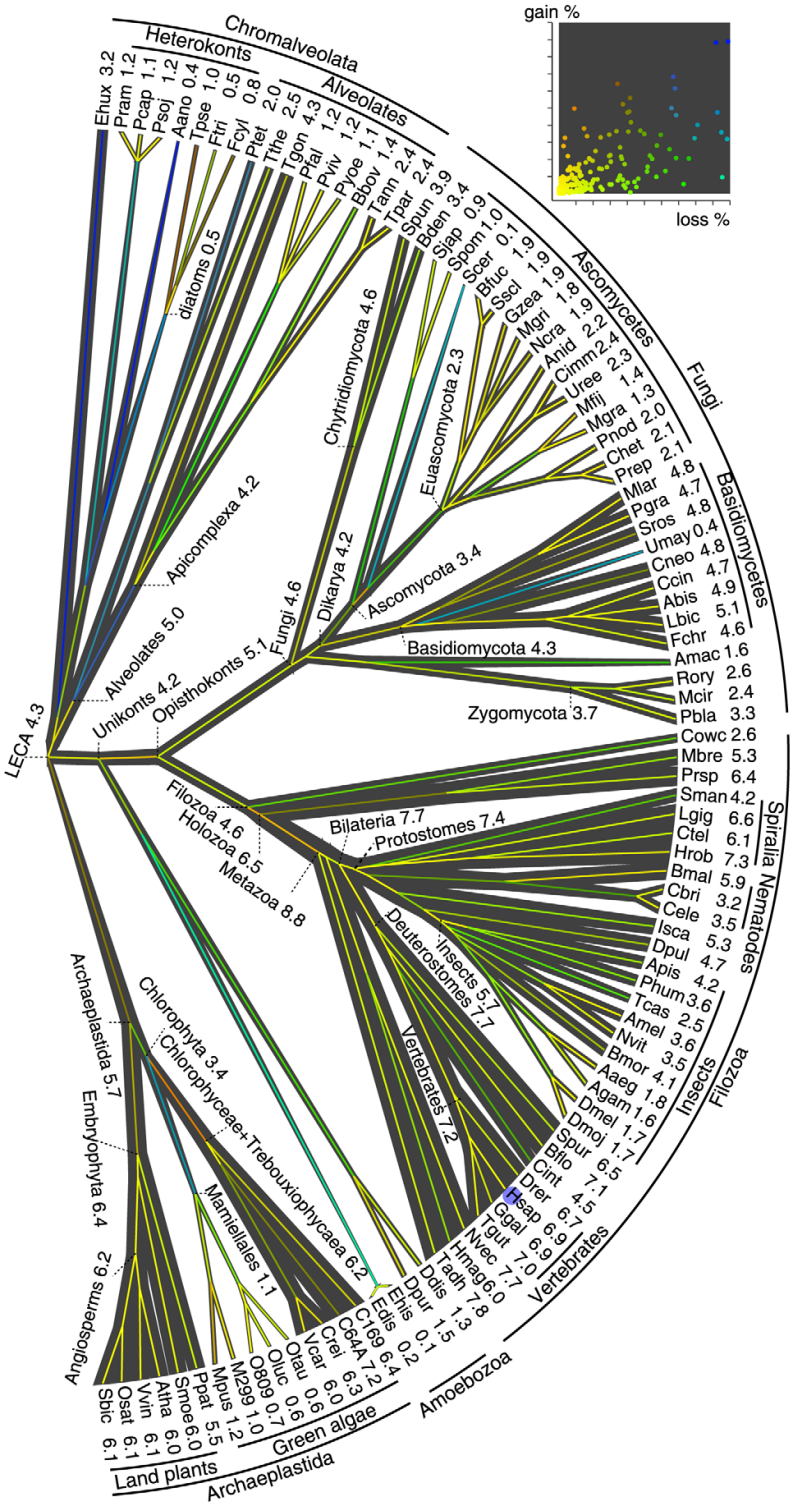
Reconstruction of intron gains and losses in the evolution of eukaryotes and intron density in ancestral eukaryote forms. Branch widths are proportional to intron density which is shown next to terminal taxa and some deep ancestors, in units of the introns count per 1 kbp coding sequence. Human (Hsap) is marked by a blue dot. Edges are colored by the relative amount of intron gain and loss, as indicated in the inset scatter plot where each point corresponds to an edge in the tree. Gain% is the percentage of introns gained in the given lineage from the parent node; loss% is the percentage of the parent's introns lost within the same lineage. Species names and abbreviations: *Aureococcus anophagefferens* (Aano), *Aedes aegypti* (Aaeg), *Agaricus bisporus* (Abis), *Anopheles gambiae* (Agam), *Allomyces macrogynus* ATCC 38327 (Amac), *Apis mellifera* (Amel), *Aspergillus nidulans* FGSC A4 (Anid), *Acyrthosiphon pisum* (Apis), *Arabidopsis thaliana* (Atha), *Babesia bovis* (Bbov), *Batrachochytrium dendrobatidis* (Bden), *Branchiostoma floridae* (Bflo), *Botryotinia fuckeliana* B05.10 (Bfuc), *Brugia malayi* (Bmal), *Bombyx mori* (Bmor), *Coccomyxa sp.* C-169 (C169), *Chlorella sp.* NC64a (C64a), *Caenorhabditis briggsae* (Cbri), *Caenorhabditis elegans* (Cele), *Coprinopsis cinerea okayama*7#130 (Ccin), *Cochliobolus heterostrophus* C5 (Chet), *Coccidioides immitis* RS (Cimm), *Ciona intestinalis* (Cint), *Cryptococcus neoformans var. neoformans* (Cneo), *Chlamydomonas reinhardtii* (Crei), *Capitella teleta* (Ctel), *Capsaspora owczarzaki* ATCC 30864 (Cowc), *Dictyostelium discoideum* (Ddis), *Dictyostelium purpureum* (Dpur), *Drosophila melanogaster* (Dmel), *Drosophila mojavenis* (Dmoj), *Daphnia pulex* (Dpul), *Danio rerio* (Drer), *Entamoeba dispar* (Edis), *Entamoeba histolytica* (Ehis), *Emiliania huxleyi* (Ehux), *Fragilariopsis cylindrus* (Fcyl), *Phanerochaete chrysosporium* (Fchr), *Phaeodactylum tricornutum* (Ftri), *Gallus gallus* (Ggal), *Gibberella zeae* PH-1 (Gzea), *Hydra magnipapillata* (Hmag), *Helobdella robusta* (Hrob), *Homo sapiens* (Hsap), *Ixodes scapularis* (Isca), *Laccaria bicolor* (Lbic), *Lottia gigantea* (Lgig), *Micromonas sp.* RCC299 (M299), *Monosiga brevicollis* (Mbre), *Mucor circinelloides* (Mcir), *Mycosphaerella fijiensis* (Mfij), *Mycosphaerella graminicola* (Mgra), *Magnaporthe grisea* 70-15 (Mgri), *Melampsora laricis-populina* (Mlar), *Micromonas pusilla* CCMP1545 (Mpus), *Neurospora crassa* OR74A (Ncra), *Nematostella vectensis* (Nvec), *Nasonia vitripennis* (Nvit), *Ostreococcus sp.* RCC809 (O809), *Ostreococcus lucimarinus* (Oluc), *Oryza sativa japonica* (Osat), *Ostreococcus taurii* (Otau), *Phytophthora capsici* (Pcap), *Plasmodium falciparum* (Pfal), *Puccinia graminis* (Pgra), *Pediculus humanus* (Phum), *Phaeosphaeria nodorum* SN15 (Pnod), *Physcomitrella patens subsp. patens* (Ppat), *Phytophthora ramorum* (Pram), *Pyrenophora tritici-repentis* Pt-1C-BFP (Prep), *Proterospongia sp.* ATCC 50818 (Prsp), *Phytophthora sojae* (Psoj), *Paramecium tetraurelia* (Ptet), *Plasmodium vivax* (Pviv), *Plasmodium yoelii yoelii* (Pyoe), *Rhizopus oryzae* (Rory), *Sorghum bicolor* (Sbic), *Saccharomyces cerevisiae* (Scer), *Schizosaccharomyces japonicus* yFS175 (Sjap), *Schistosoma mansoni* (Sman), *Selaginella moellendorffii* (Smoe), *Schizosaccharomyces pombe* (Spom), *Spizellomyces punctatus* DAOM BR1173 (Spun), *Strongylocentrotus purpuratus* (Spur), *Sporobolomyces roseus* (Sros), *Sclerotinia sclerotiorum* 1980 UF-70 (Sscl), *Trichoplax adhaerens* (Tadh), *Theileria annulata* (Tann), *Tribolium castaneum* (Tcas), *Toxoplasma gondii* (Tgon), *Taenopygia guttata* (Tgut), *Theileria parvum* (Tpar), *Thalassiosira pseudonana* (Tpse), *Tetrahymena thermophila* (Tthe), *Ustilago maydis* 521 (Umay), *Uncinocarpus reesii* 1704 (Uree), *Volvox carteri* (Vcar), *Vitis vinifera* (Vvin).

The present analysis of gene structure evolution included an extensive data set of sequenced and annotated genomes from the Unikonta (the Opisthokont group that combines animals and fungi, together with Amoebozoa), the Archaeplastida (green algae and land plants), and Chromalveolata (Heterokonta and Alveolata). Of the five supergroups of eukaryotes [24], [25], [26], only these three are currently represented by multiple genomes with broad ranges of intron densities. There are no sequenced genomes for the supergroup of Rhizaria. The fifth supergroup, Excavata, includes mostly parasitic forms with very few introns and only one sequenced genome of a free-living organism, *Naegleria gruberi*, with a moderate intron density [27], which renders ancestral reconstruction moot within this supergroup. Thus, our data set effectively covers the entire available diversity of eukaryotic genomes. The evolutionary relationships between the supergroups remain uncertain [26], [28], so they are represented as a trifurcation in the schematic evolutionary tree shown in Figure 1. We identified large orthologous protein-coding gene sets that are represented in a substantial majority of the analyzed genomes using a procedure that combined ortholog clustering and gene-species tree reconciliation techniques (see Methods and Supporting Text S1 for details). The encoded protein sequences from each of the orthologous gene sets were aligned and projected onto the coding nucleotide sequences, annotated with the exon-intron structures. The data set was further filtered to exclude aligned positions with significant ambiguity (see Methods and Text S1 for details). The final data set contained 8403 intron presence-absence profiles from 245 sets of orthologous genes.

Intron loss and gain were modeled using a probabilistic Markov model encompassing lineage-specific loss and gain rates, as well as rate variation across sites. The Markov Chain Monte Carlo (MCMC) method [29] was employed to sample model parameters and ancestral reconstructions by their posterior distributions, and to infer ancestral states along with the respective Bayesian confidence intervals (see Methods and Supporting Text S1 for details). Experiments with various rate variation models across sites showed that only the loss rate variation had a significant impact on the model fit (Figure 9 in Supporting Text S1). Thus, it appears that, when uniform site preferences that apply across all eukaryotes are considered, introns in certain positions are prone to be lost significantly more often than others whereas no sites are significantly more prone to intron gain.

This reconstruction provides a thorough view of the evolution of gene structure across three eukaryotic supergroups and reveal several general trends (Figure 1 and Supporting Figure S1). Most lineages show net intron loss that can be substantial as in alveolates, some lineages of fungi, green algae and insects, or well-balanced by concomitant intron gains as in land plants [30], most animal lineages, and some fungi [31]. Massive intron gains were inferred only for several deep branches, most conspicuously, the stem of the Metazoa, and to a lesser extent, the stems of Mamiellales (a branch of green algae), Viridiplantae, Opisthokonta, and Metazoa together with Choanoflagellata (Figure 1). These findings vindicate, on a much larger data set and with greater confidence, the previous conclusions that intron gain was rare during evolution of eukaryotes compared to intron loss. Episodes of substantial intron gain seem to coincide with the emergence of major new groups of organisms with novel biological characteristics such as Metazoa [19].

**Figure 2 pcbi-1002150-g002:**
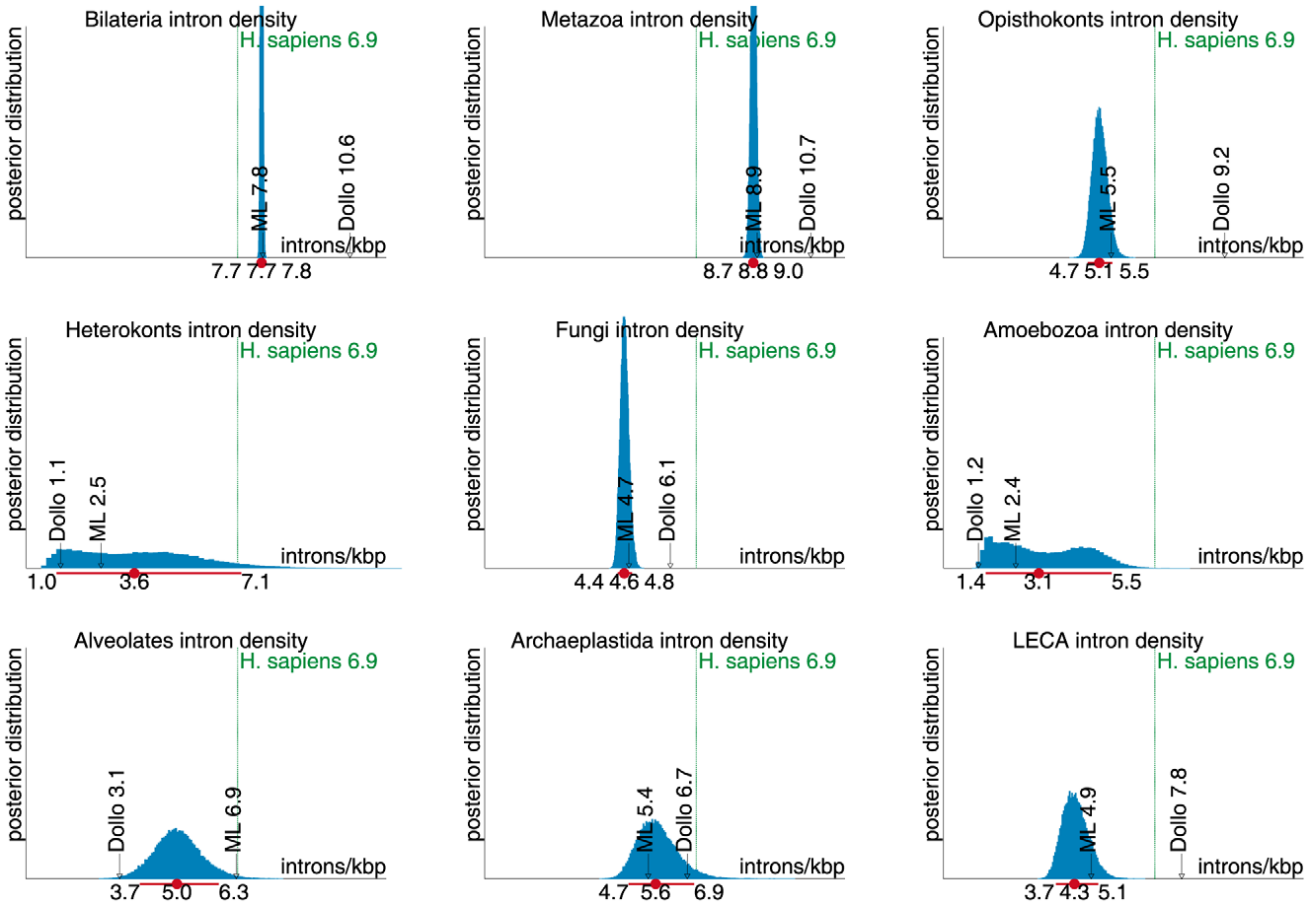
Inferred ancestral intron densities and confidence intervals. The plots for 9 key ancestral forms show the posterior distributions of the ancestral intron density inferred from the sampling chains. On each plot, the horizontal red line shows the median (the dot) and the 95% (+/−47.5%) confidence interval around it, estimated from 50,000 sampled MCMC steps.

**Figure 3:**
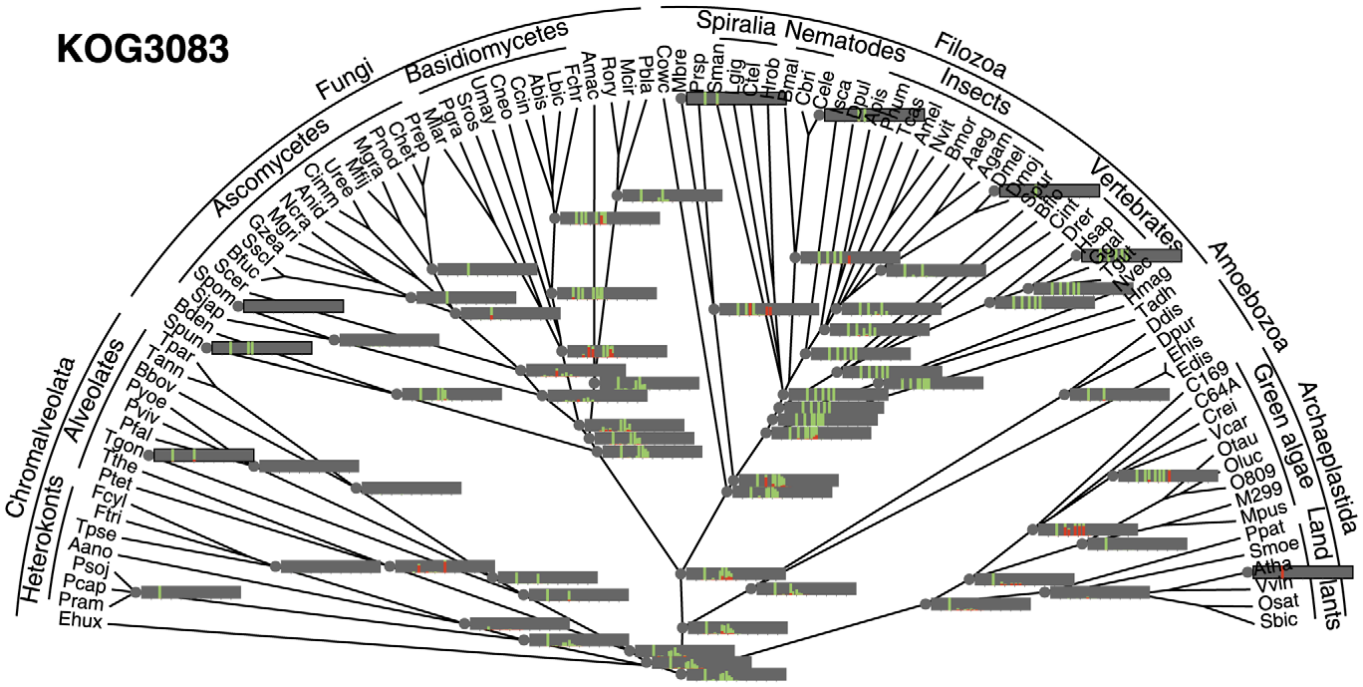
Inferred intron site histories in prohibitin orthologs (KOG3083). The tree from Figure 1 is used as the template for the reconstruction. Vertical bars are placed at intron sites proportionally along the X axis within the bars with respect to the underlying alignment. The height of green bars is proportional to the probability of intron presence; the height of red bars is proportional to the probability of intron gain in the lineage leading to the node.

Several previous studies, performed on much smaller data sets and with less robust reconstruction methods, have suggested that at least some eukaryotic ancestral forms could have possessed intron-rich genes [19], [20], [31]. In particular, we found previously that the last common ancestors of Chromalveolata and particularly Alveolata could possess high intron densities despite the fact that all extant genomes available for in these groups are intron-poor [22]. The present analysis reinforces these conclusions by inferring high intron densities for the ancestors of each major group of eukaryotes within each of the three supergroups (Figures 1, 2, and Supporting Figure S1). The implication is that, whenever an extant eukaryotic genome shows a low intron density, this intron-poor state is a result of extensive, lineage-specific intron loss. Inspection of individual intron site histories revealed the same trends (see Figure 3 and Supporting Video S1). For example, Figure 3 shows the reconstructed history of intron loss and gain in the gene that encodes the membrane protease prohibitin. For this gene, a relatively high intron content was reconstructed for LECA, with four or five introns most likely present in the ancestral gene. The subsequent evolution of this gene involved multiple, parallel loss of introns in most of the eukaryotic lineages. Substantial intron gain is inferred only for Metazoa, one lineage of fungi, and one lineage of green algae. Notably, the intron content in mammals is the same as the inferred intron content of LECA (five introns), and there is no intron-poor stage on the path from LECA to mammals (Figure 3).

In addition to the Bayesian MCMC estimates, we inferred ancestral densities by using Dollo parsimony [32], and by the posterior distributions in the maximum-likelihood (ML) model derived during the MCMC sampling. More precisely, the posterior reconstruction uses a fixed parameter set (the ML model) and infers a “plausible” history by computing the posterior probability of intron presence for every site at each ancestral node. Posterior probabilities are summed across sites to yield expected values [33] which can be interpreted as a parsimonious reconstruction weighed by the inferred lineage- and site-specific predispositions for loss and gain. The results of the comparison between the reconstructions obtained with the three methods indicate that parsimony reconstructions introduce a noticeable bias.

The Dollo and ML estimates show a picture of intron-rich eukaryotic ancestors that is qualitatively similar to the MCMC results. Quantitatively, similarly to the case of ancestral molecular sequence reconstruction [34], the Bayesian estimates often disagree with the parsimony reconstruction. Specifically, the MCMC sampling showed the tendency to infer higher ancestral densities (15–17% higher at intron-rich ancestors; see Figure 11 in Text S1) than Dollo parsimony, with the exception of the ancestors along the lineage from LECA to protostomes, for which Dollo parsimony yields up to 45% higher densities (see Figure 11 in Text S1). The differences highlight the idiosyncrasies of ancestral reconstruction methods and the pitfalls of disregarding model uncertainties. Dollo parsimony places the origin of introns at the most recent common ancestor of intron-bearing terminal taxa at each site, thereby systematically underestimating intron age and parallel gains. In contrast, ML infers similar ancestral reconstructions as MCMC (Figure 11 in Text S1), and the ML model parameters are not very different from the sampled model parameters (93% of the ML parameters fall within the 95% confidence intervals; see Figure S12 in Supporting Text S1).

The MCMC sampling procedure provides robust statistical estimates of ancestral states through Bayesian confidence intervals. The 95% confidence intervals are fairly tight around most estimates, even for such deep ancestors as those of alveolates (3.7–6.3 introns/kilobase), Dikarya (“higher” fungi: 3.7–4.7 introns/kilobase), opisthokonts (4.7–5.5 introns/kilobase) and, most importantly, LECA (see below). The uncertainty is larger in ancestors with subsequent turbulent history in the descendants. A case in point is the amoebozoan ancestor. There was extensive intron loss along the branch leading from the intron-rich unikont ancestor to the extant Amoebozoa. It is unclear, however, whether the losses occurred in parallel in multiple descendant lineages, or prior to the split between *Dictyostelium* and *Entamoeba* (see Figure 4 in Text S1). Even more problematic is the reconstruction of the gene structure evolution in chromalveolates, because of the extensive intron turnover in many lineages within this supergroup. Indeed, there was no detectable intron conservation across haptophytes (*E. huxleyi*), pelagophytes (*A. anophagefferens*), diatoms, and other eukaryotes within or outside chromalveolates (see Table 6 in Text S1). For instance, the diatom *T. pseudonana* shares only 25% of introns with other diatoms in the data set, and only 3–6% with other eukaryotes. For comparison, human intron positions show 75–80% conservation with other Metazoa and 25–30% conservation with plants. Introns of *Phytophthora* and alveolates are also often conserved across large evolutionary distances. Accordingly, the reconstruction is fairly certain for the alveolate, Phytophthora and diatom ancestors and their descendants, and even for the chromalveolate ancestor, but many equally plausible scenarios are apparent for haptophyte ancestors (see Figure 5 in Supporting Text S1). Exploration of alternative phylogenies for the major chromalveolate groups yielded neither a better model fit, nor more precise estimates (data not shown). These examples demonstrate the inherent uncertainties in ancestral reconstruction. Conceivably, the extensive intron turnover in chromalveolate algae, and the massive loss in Amoebozoa all but effaced any clues as to the ancestral gene structures, illustrating the fundamental limits of the reconstruction [35].

The gene architecture of LECA is of special interest. Previous estimates of intron density for LECA were very uncertain due to methodological problems with maximum likelihood inference [19]. The present reconstruction yielded the median value of 4.3 introns/kilobase, with the 95% confidence interval of 3.7–5.1 introns/kilobase (Figure 2), i.e., 53–74% of the human intron density with a 95% confidence. Different resolutions of the trifurcating plant-unikont-chromalveolate root did not significantly affect the model fit (see Figure 9 in Text S1). Our analysis of the gene structure in the only sequenced genome of a free-living excavate (a member of a fourth supergroup of eukayotes), *Naegleria gruberi* [27], identified a high fraction (30–50%) of intron positions shared with other supergroups (see Table 14 in Supporting Text S1), an observation that is compatible with an intron-rich LECA and with a moderate intron turnover within the line of descent leading from the LECA to *Naegleria*.

Strikingly, the greatest intron density among all ancestral and extant eukaryotes was inferred for the last common ancestor of the Metazoa, at 120–130% of the human density, with a 95% confidence (Figures 1 and 2).

We validated the inference procedures by simulating the evolution of intron sites (see Figure 13 in Supporting Text S1). The MCMC and ML methods infer the ancestral intron densities with no obvious bias, concurring on simulated data to a similar extent as on the main data set. In a sharp contrast, Dollo parsimony is significantly biased towards overestimation at many intron-rich ancestors. The variance of the probabilistic estimators at different ancestral nodes recalls the spread of Bayesian confidence intervals: fairly small variance was observed for almost all nodes including the LECA but the inferences for the amoebozoan and heterokont ancestors were unreliable. Additional simulation experiments (see Figure 13 in Supporting Text S1) showed that the probabilistic models performed robustly even in the presence of missing orthologs, or heterotachious model violations.

In all eukaryotes, with the interesting exception of the tunicate *Oikopleura dioca* [36], introns show a non-uniform phase distribution, i.e., an excess of introns that are inserted between codons (phase 0) compared to introns between codon positions 1 and 2, and 2 and 3 (phases 1 and 2, respectively) [16], [37]. We compared the inferred phase distributions for the gained, lost and ancestral introns (or, in other words, derived the phase-specific gain and loss rates, and ancestral states). In most animals, including the ancestral forms, and in LECA, the ratios of the three phases remained nearly constant at 2∶1∶1 (twice as many introns of phase 0 as there were introns of phase 1 or 2). In some of the fungi and chromalveolates, the excess of phase 0 introns was less pronounced, whereas in plants, there was a greater than average excess of phase 0 and a paucity of phase 1 introns (see Figure 7 and Table 8 in Text S1). These findings indicate that the excess of phase 0 was a (nearly) universal feature of intron evolution throughout the history of eukaryotes but also reveal significant deviations from this pattern in some lineages. The mechanistic basis of both the ancestral excess of phase 0 and the lineage-specific variations remains to be identified.

The results of this study reveal three principal modalities of evolution of the eukaryote gene structure:

- relative stasis accompanied by slow, roughly uniform loss of introns
- extensive loss of ancestral introns that in many lineages led to nearly intronless genomes
- extensive turnover of introns when the high loss rate is (approximately) offset by a high gain rate.

The choice between these routes of evolution in a particular lineage could depend primarily on the intensity of purifying selection that is linked to the effective population size [38], [39]. Periods of large effective population size entail strong purifying selection and create a ratchet effect whereby lost introns are unlikely to be regained. Remarkably, the line of descent from LECA to mammals seems to have never gone through a strong selection stage, so the intron density remained continuously high, the only major perturbation being the gain of many introns at the onset of animal evolution followed by subsequent gradual loss (Figure 1).

## Discussion

The results of this work, thanks to the extensive data set of analyzed genomes and the robust reconstruction method that yields inferences of ancestral states with minimal uncertainty, seem to close the debate on the gene architecture of ancestors of extant eukaryotes including LECA. It is now clear that the genes of ancestral eukaryotes possessed high intron density, close to the densities in the most intron-rich modern genomes, those of mammals.

This finding has substantial implications for understanding the evolution of eukaryotes. It has been noticed that intron-poor genomes typically possess strong, highly efficient splice signals, whereas intron-rich genomes contain mostly weak, error-prone splice signals [40], an effect that appears to be due primarily to weak purifying selection that precludes both purging of introns and tightening of the junctions (splice signals) [41]. In intron-rich ancestral genomes, frequent errors of splicing yielding aberrant transcripts were inevitable. The abundance of such transcripts was the driving force behind, first, the evolution of defense systems that attack immature mRNAs and prevent their translation, like the nonsense-mediated decay (NMD) system that also contributes to expression regulation [42], [43], and second, the recruitment of aberrant transcripts to produce variants of proteins, the trend that in animals gave rise to the pervasive alternative splicing, one of the principal mechanisms of diversity generation and protein function regulation [12], [14], [44].

Remarkably, the present results indicate that the entire line of descent from LECA to mammals was a continuous intron-rich state (Figure 1) that provided for uninterrupted evolution of the growing repertoire of functional alternative spliced forms. The unprecedented intron gain at the onset of animal evolution could further contribute to the expansion of alternative forms. This spurt of intron gain might have resulted from a combination of a population bottleneck that led to weak purifying selection with increased transposon activity that could activate double-strand break repair, a likely major mechanism of intron gain [45].

## Methods

Orthologous genes were identified using a modification of the previously described procedure [22]. The groups of putative orthologs from eukaryotes from the eggNog database [46] were employed as “seeds” to which members from the 99 selected genomes were added. The resulting candidate sets of orthologs were further filtered by verifying their phylogenetic relationships. In particular, a non-negative log-likelihood ratio between the neighbor- joining tree and the known species phylogeny, computed by PhyML (Guindon and Gascuel, 2003) was required. The adopted phylogeny reflects known evolutionary relationships between major taxonomic groups [24], [26]. Sequences of *Naegleria gruberi* were selected using the same procedure, but the large evolutionary distance precluded identification of a sufficient number of orthologs and unambiguous alignment of splice sites. Therefore, sequences from *N. gruberi* were not included them from the ancestral inference.

The intron positions were mapped onto gene sequences using a previously developed computational pipeline [22]. The resulting data set is a table of intron absence and presence in which each column corresponds to a splice site projected onto an unambiguous alignment column (retaining intron phase information), and each row corresponds to one of the 99 species. Table entries may be 1 (splice site is present), 0 (no splice site), or “*” (ambiguous) for a missing ortholog or an uncertain alignment portion. The final table was produced using the Malin software [47] and contained all columns with at most 24 ambiguous entries (and at least one entry of 1).

Gene structure evolution was modeled mathematically by assuming that the table columns 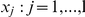 are independent and identically distributed random vectors. The distribution itself incorporates variable intron gain and loss parameters across lineages and splice sites *(16,40)*. For a formal treatment, define *T* as the known phylogeny for the terminal taxon set *S*, i.e., a rooted tree with *n* leaves that are bijectively labeled by taxa from *S*. Internal tree nodes correspond to common ancestors. The history of a potential splice site is modeled as a binary labeling of all tree nodes: ξ = (ξ[*u*]**∈**{0,1}: *u* **∈** *T*). In a Markov model, the labeling is randomly drawn from a distribution for which the parent-child relationships in the phylogeny define conditional independencies. The distribution of ξ at a site is fully determined by the presence probability at the root π = Pr{ξ[root] = 1}, and edge-specific rates 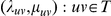. On the edge *uv*, labels change with probabilities
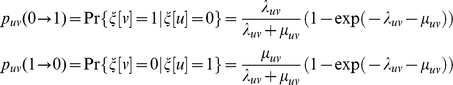



Conversely, 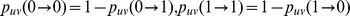. The rates are set on each edge *uv* as 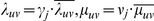 where γ, ν are site-specific rate multipliers, and 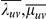 are lineage-specific average rates. The site-specific rate multipliers are drawn independently from discretized Gamma distributions [48] with the mean of 1. The model is thus completely specified by the vector 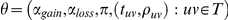, where the hyperparameters α specify the shape of the Gamma distribution for the site-specific rate multipliers, and the edges are parametrized by their *length* and *rate ratio* 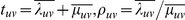, respectively. An input table column is a vector 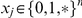, where the character * denotes ambiguity. Accordingly, equivalence between resolved and unresolved labelings is defined by
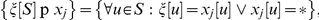
where ξ[*S*] is a random leaf set labeling. The model defines the likelihood 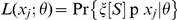 for each table column. The likelihood for the complete data set, defined as
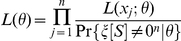
can be computed efficiently for a given model parametrization θ, and numerically optimized to find the maximum-likelihood parameters θ* [22], [33]. The condition in the denominator accounts for the lack of columns with no splice site (entry 1) at any terminal taxon.

Ancestral intron counts were inferred using three methods. Intron count estimates were converted into densities by the formula density = intron count·6.946 kbp^−1^/875. The conversion formula uses human as a reference: 6.946 is the mean number of human introns per 1000 base pairs (kbp) in the coding sequences of the analyzed genes, and 875 is the number of human introns in the data set. The posterior distribution for ancestral intron counts for a given model parametrization is computable without much difficulty [33], and was used to infer the ancestral densities in conjunction with the maximum-likelihood model found during MCMC sampling, as implemented in the Malin software [47]. The ancestral intron positions were also inferred by using the Dollo parsimony principle, as implemented in Malin [47].

In order to estimate ancestral intron densities and lineage-specific changes in a Bayesian setting, we adapted mutation mapping techniques commonly employed with molecular sequence evolution models [34]. The Metropolis- Hastings algorithm [49] was used to estimate the posterior distributions for ancestral reconstructions and model parameters in a Markov-chain Monte Carlo framework [29]. The SAMPLING algorithm (Box 1) generates a random walk by a Markov chain over the parameter space and ancestral reconstructions.

**Box 1. SAMPLING algorithm**

 S1. draw random initial parameters θ by their prior distribution P(θ)

 S1. **repeat**

 S3. propose new random model parameters θ′ by distribution Q(θ→θ′)

 S4. with probability 
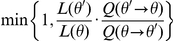
, set θ = θ′

 S5. generate random ancestral labeling by posterior probabilities

In Line S4, the acceptance probability includes the likelihoods L(θ) at different model parameters, the prior distribution P(θ) of parameters, and a proposed model distribution Q(θ→θ′). In Line S5, random ancestral labelings 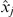 are drawn at each column *j* by using the so-called conditional likelihoods for labeling node *u* with *x* = 0, 1, given the (possibly unresolved) labelings at the terminal taxa *S_u_* within the subtree rooted at *u* :

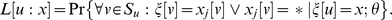



The conditional likelihoods are calculated by dynamic programming in a postorder traversal by adapting the pruning algorithm of Felsenstein [50] (LABELING algorithm, Box 2).

**Box 2. LABELING algorithm**

L1. draw random site-specific rate multipliers γ, ν

L2. set 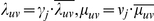 on every edge *uv*

L3. compute conditional likelihoods *L*[*u*∶*x*] for all nodes *u* and labels *x* = 0,1

L4. set 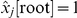 with probability 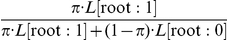; otherwise set 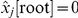

L5. **for** all non-root nodes *v* in a preorder traversal **do**

L6. set 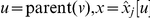

L7. with probability 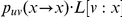, set 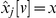; otherwise set 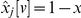

In Line L1, the rate multipliers are drawn from the posterior distribution for the different discretized rate categories using the shape parameters of the respective Gamma distributions. The SAMPLING algorithm generates a Markov chain for pairs of model parameters and ancestral reconstructions. The equilibrium distribution for the chain is the posterior distribution
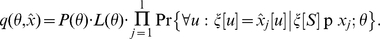



In addition to sampling histories of profiles from the input data, we also generated “all-absent” profiles with introns missing at every terminal taxon [33]. The history of all-absent profiles was randomly sampled with the same procedure, and the number of such profiles was set as a negative binomial random variable with parameters 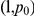, where 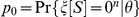 is the probability of an all-absent profile. Ancestral intron counts were computed by tallying 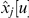 across all *j*, and adding the analogous sum for the sampled histories of all-absent profiles. Intron gains and losses on branches were estimated with a similar calculation.

The prior distribution P(θ) was uniform for every parameter (and thus absent from the formula in Line S4): over the range [0, 10] for shape parameters and edge lengths, and over the range [0, 1] for π and the rate ratios. In a typical MCMC proposal, a subset of model parameters was chosen, and then multiplied by a random value; see Text S1for the details of the proposal distributions Q(θ→θ′).

The convergence and the mixing efficiency were assessed by running 100 chains in parallel (see Figures 1–3 in Text S1). Estimates were computed using 50,000 independent samples from the joint posterior distribution *q* of parameters and ancestral intron densities.

Individual intron site histories were reconstructed using the Malin software [47] with the median parameter values taken from the MCMC sampling.

Simulations were performed by generating 100 random data sets of a comparable size to the input data set using the MCMC median model parameters, coupled with an erasure procedure simulating missing orthologs, or randomly generated multipliers for simulating heterotachy (lognormal multipliers for rate parameters, exponential multipliers for edge lengths): see Figure 13 in Supporting Text S1.

## Supporting Information

Figure S1 Posterior distributions of the ancestral intron densities inferred from the sampling chains for all ancestral forms.
(PDF)

Text S1 Detailed methods and results with the illustrating figures and tables.
(PDF)

Video S1 Dynamic representations of the histories of intron loss and gain for the 245 analyzed clusters of orthologous genes.
(MOV)
