# Supplementary material for: A Detailed History of Intron-rich Eukaryotic Ancestors Inferred from a Global Survey of 100 Complete Genomes
Source: PLoS Comput Biol. 2011 Sep 15;7(9):e1002150. doi: 10.1371/journal.pcbi.1002150 (PMC3174169; doi:10.1371/journal.pcbi.1002150)
Supplement: Figure S1 — Posterior distributions of the ancestral intron densities inferred from the sampling chains for all ancestral forms. (PDF) [file pcbi.1002150.s001.pdf]

A detailed history of intron-rich eukaryotic ancestors inferred from a global survey of 100 complete genomes

Supporting Figures S10.i–xcvii

Miklós Csűrös Igor B. Rogozin Eugene V. Koonin

May 23, 2011

The following plots show the posterior distribution of the ancestral intron density inferred from the sampling chains. On each plot, the horizontal red line shows the median (the dot) and the 95% (±47.5%) credible interval around it estimated from 50000 subsampled MCMC steps.

Phylogeny: ancestral node names

Trifurcating LECA

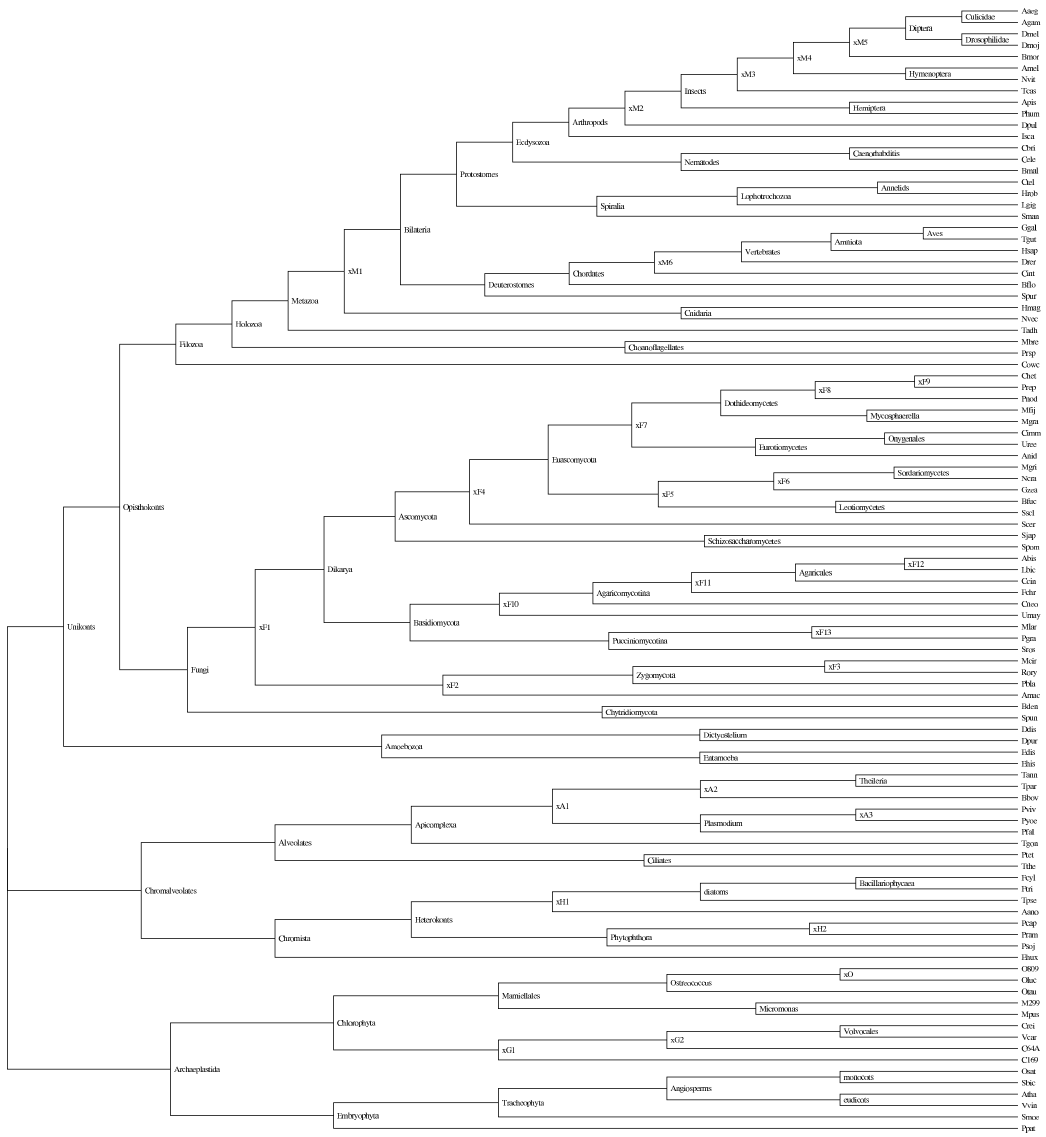

Reconstructions with some uncertainty

S10.i Agaricomycotina

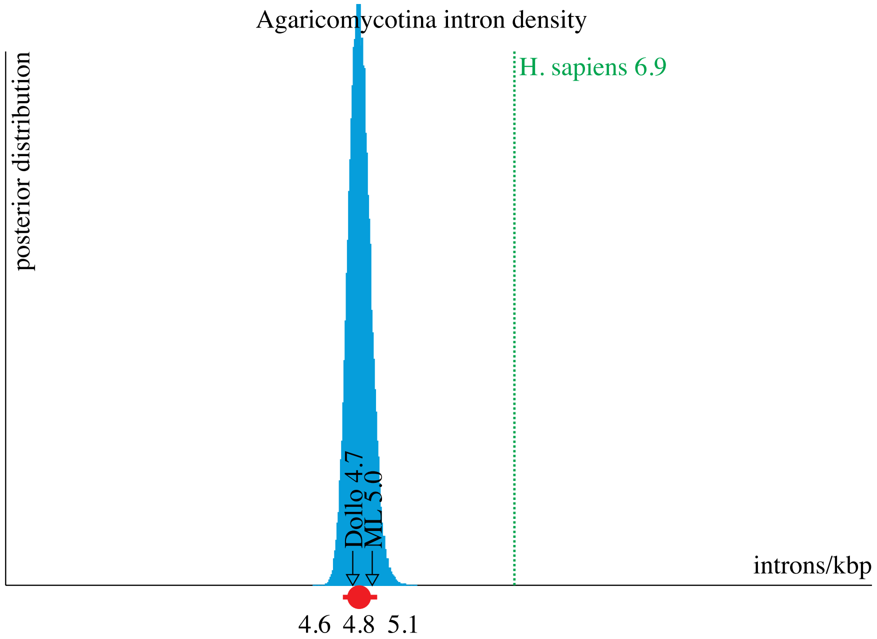

S10.ii Alveolates

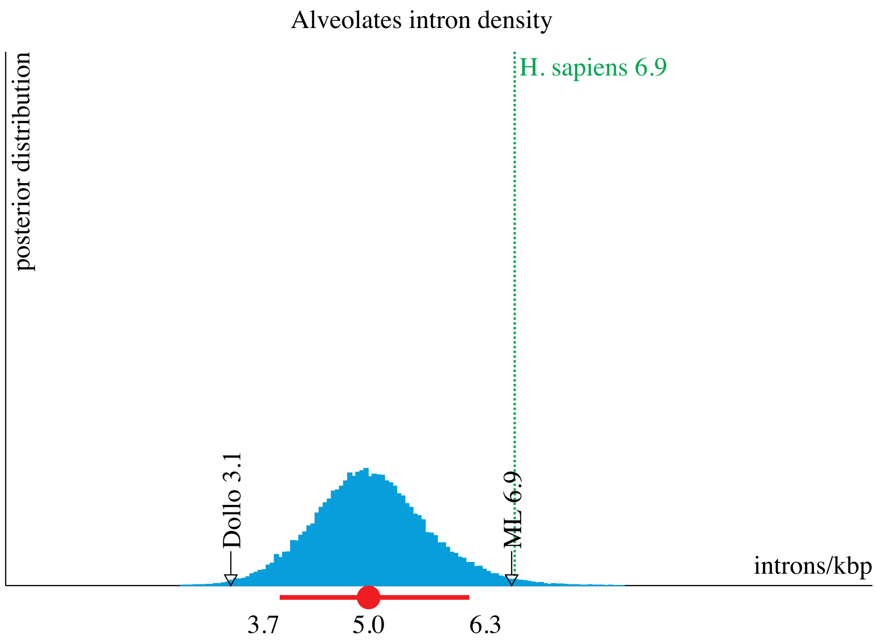

S10.iii Amoebozoa

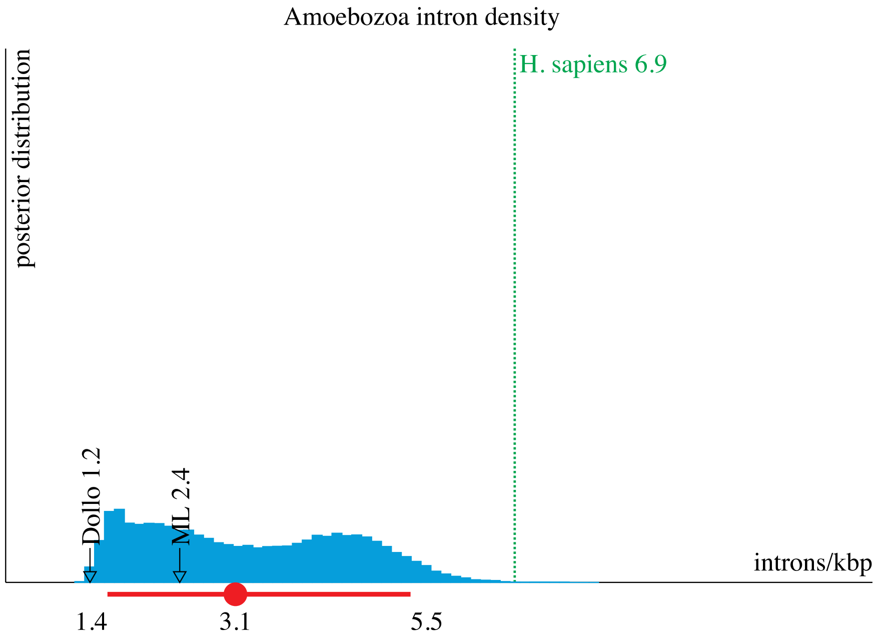

S10.iv Angiosperms

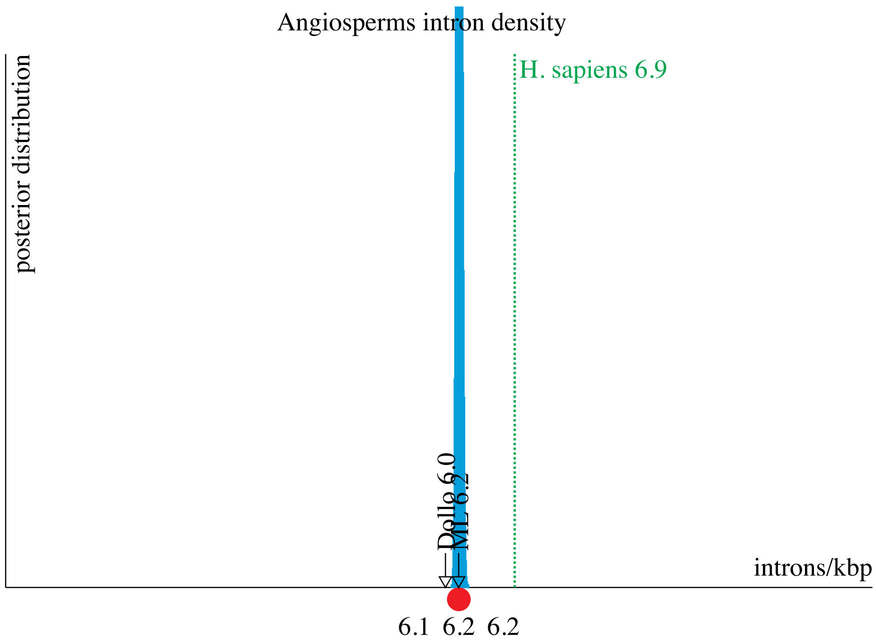

S10.v Annelids

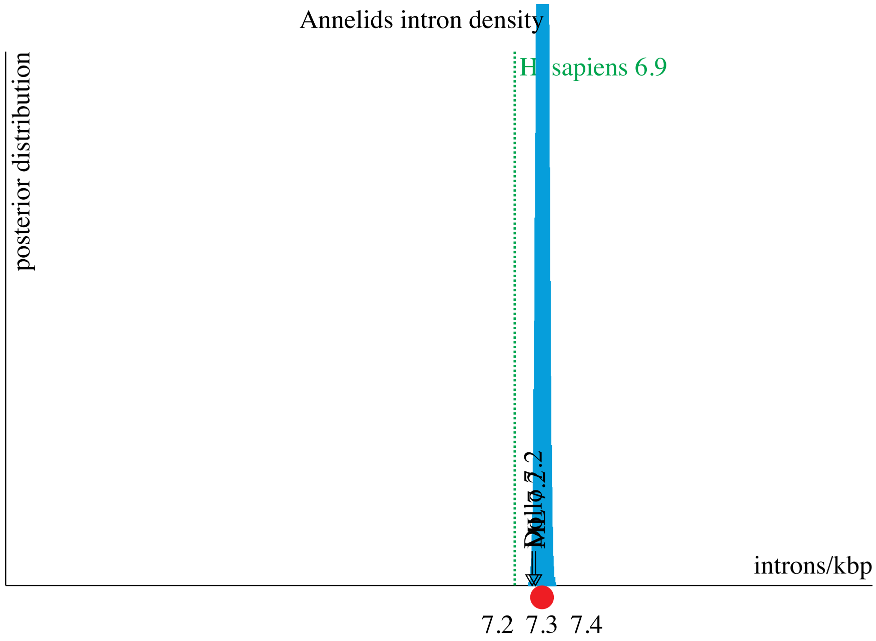

S10.vi Apicomplexa

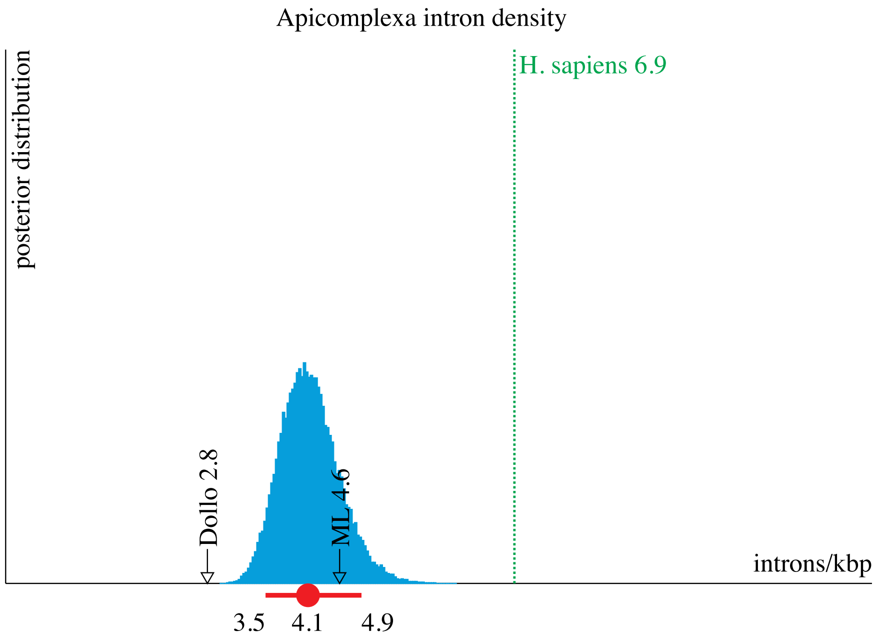

S10.vii Arthropods

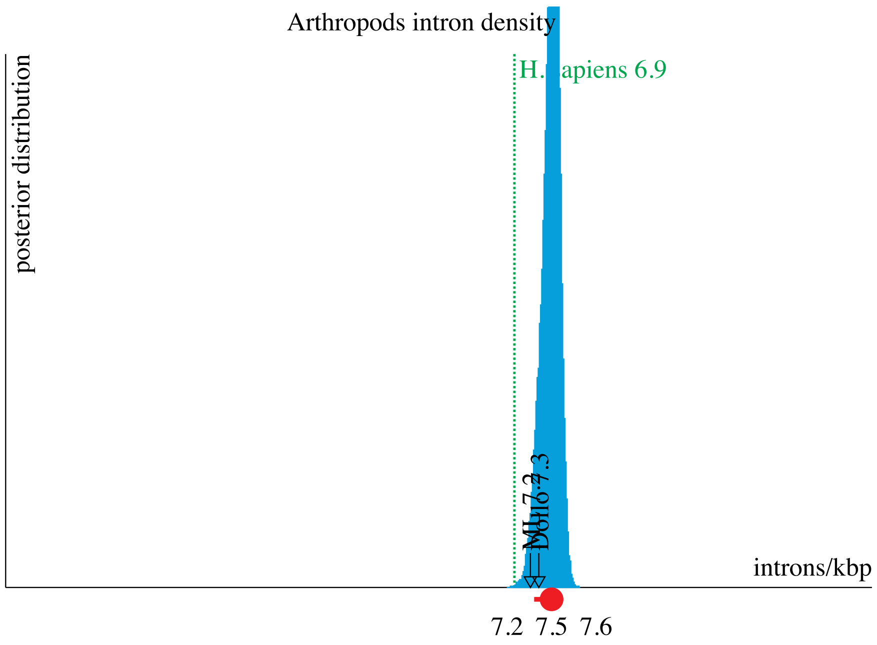

S10.viii Ascomycota

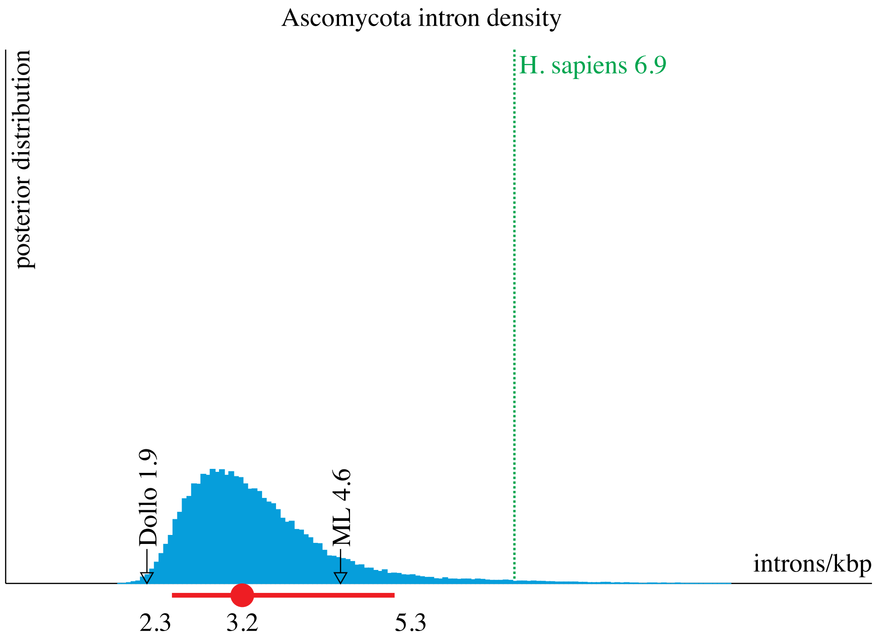

S10.ix Basidiomycota

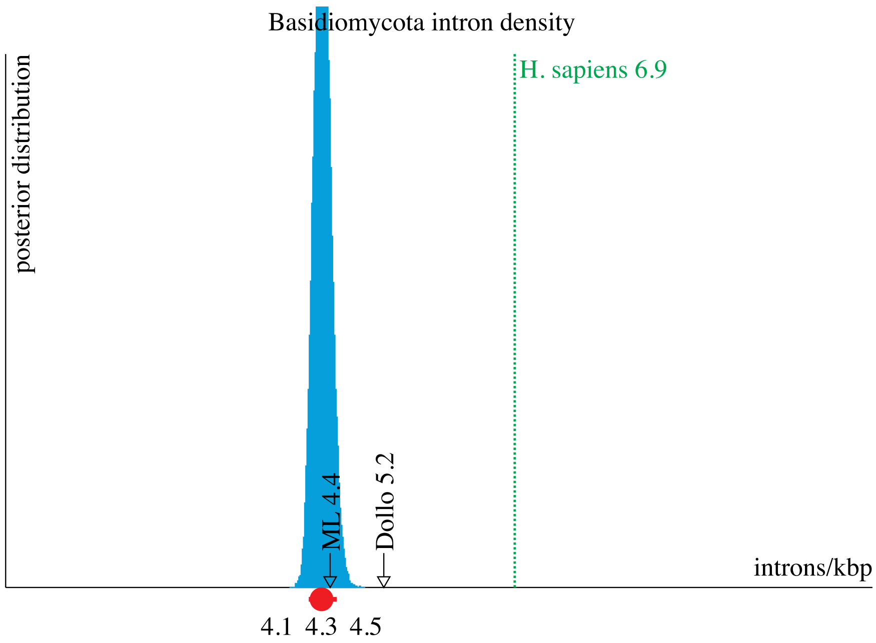

S10.x Bilateria

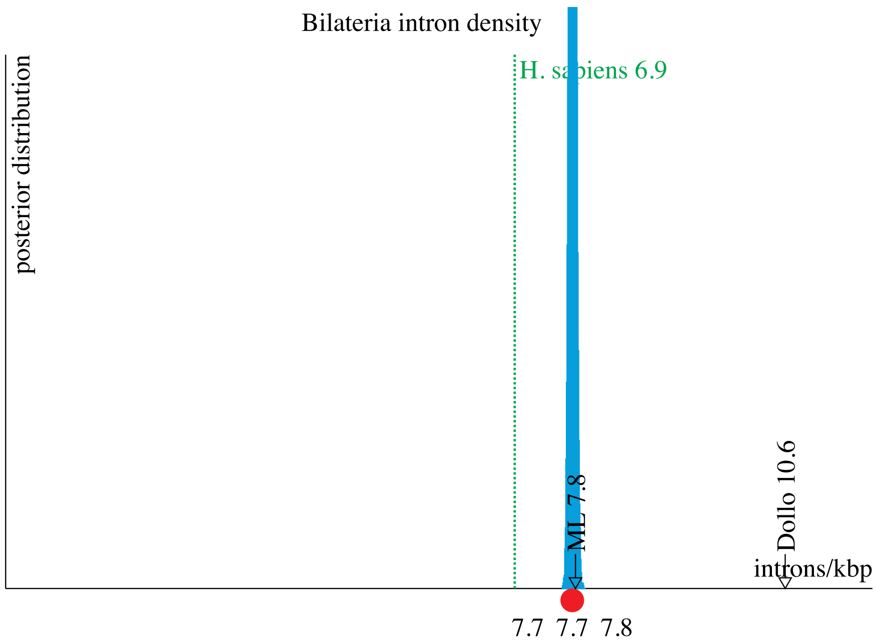

S10.xi Chlorophyta

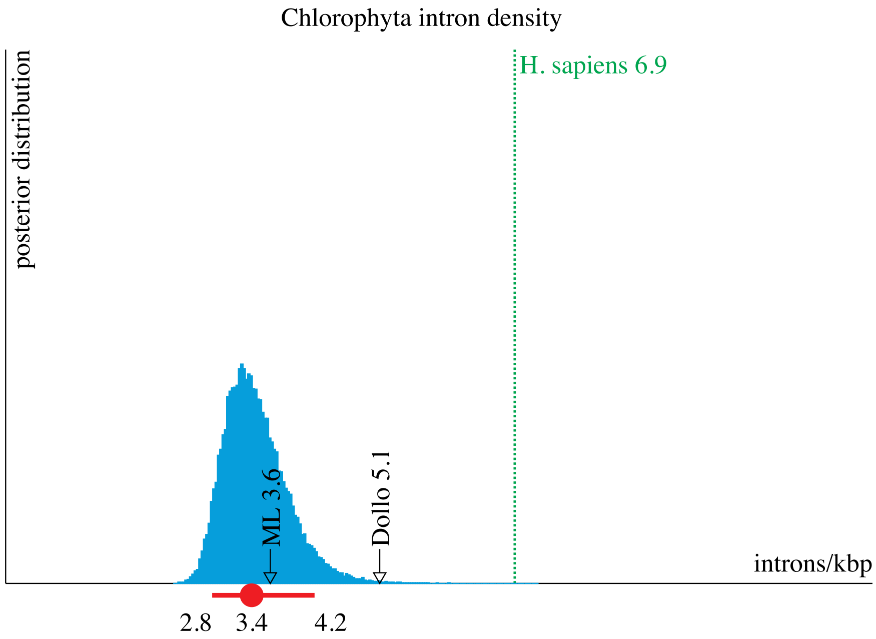

S10.xii Choanoflagellates

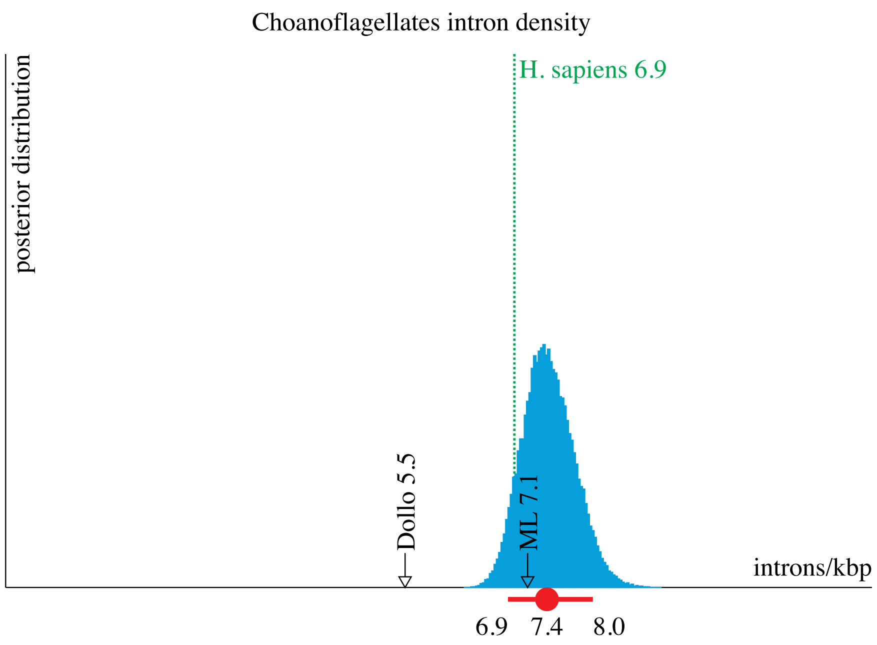

S10.xiii Chordates

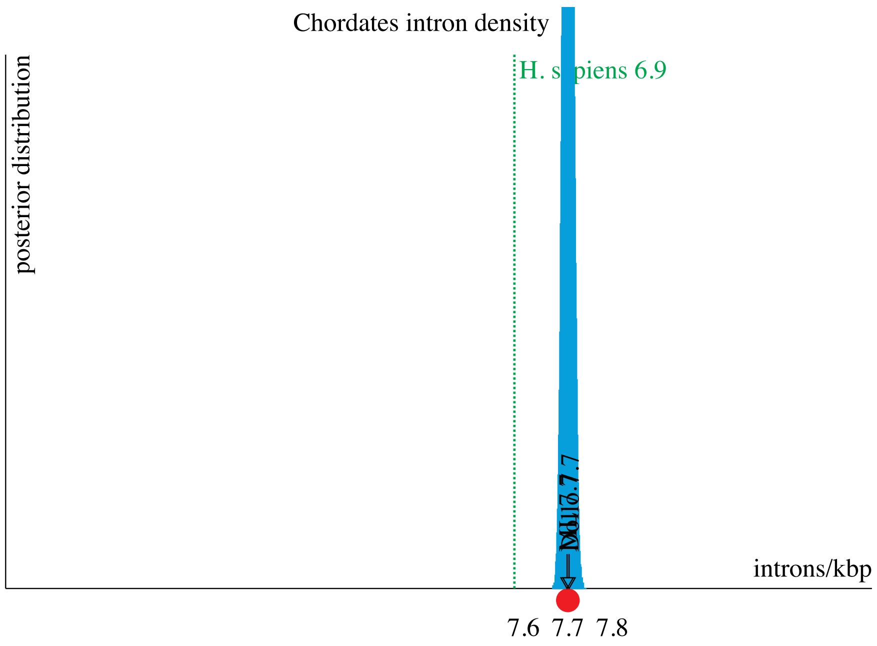

S10.xiv Chromalveolates

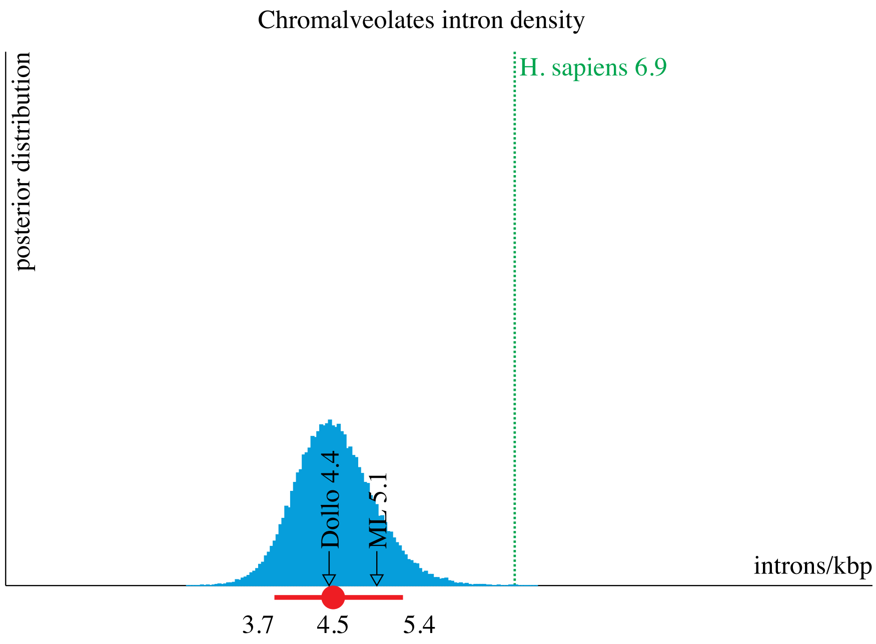

S10.xv Chromista

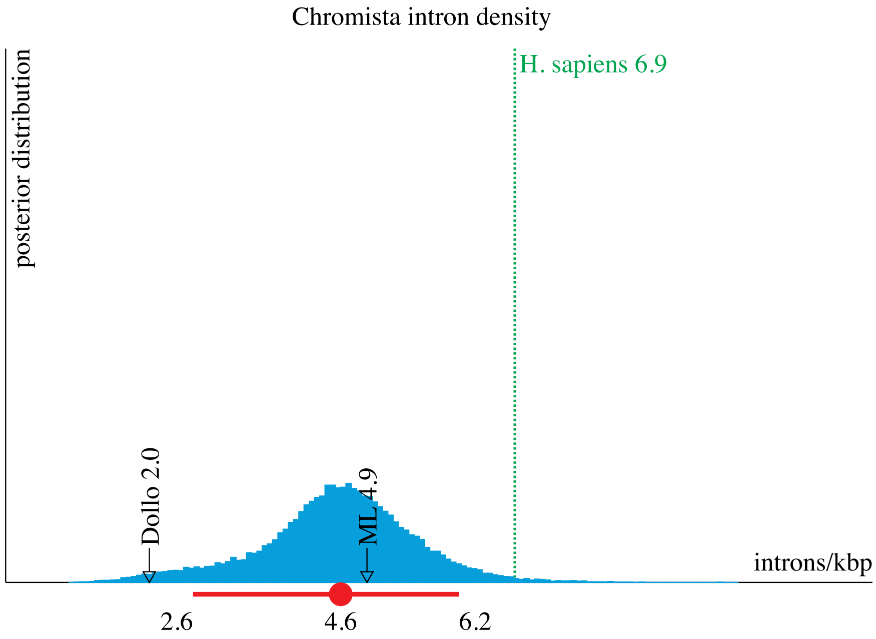

S10.xvi Chytridiomycota

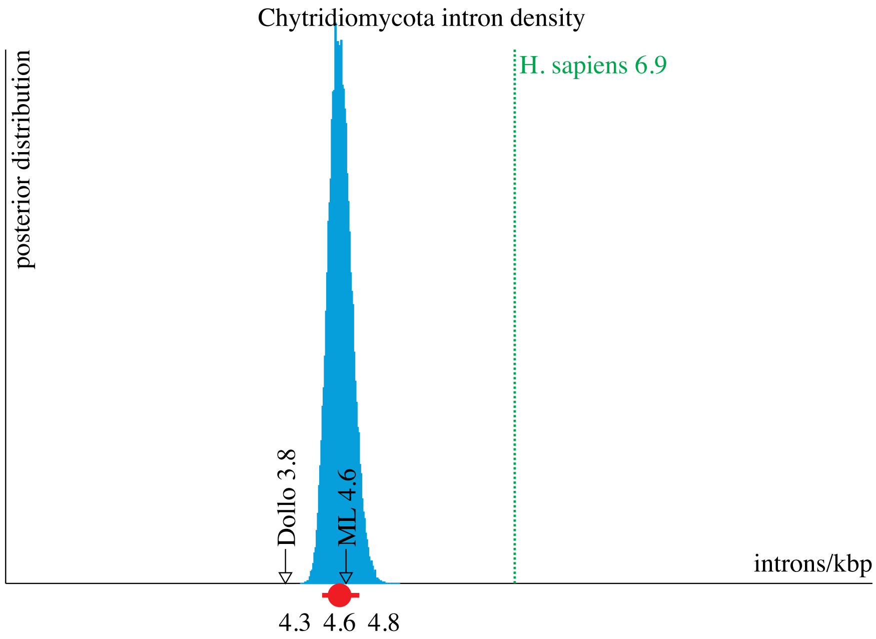

S10.xvii Ciliates

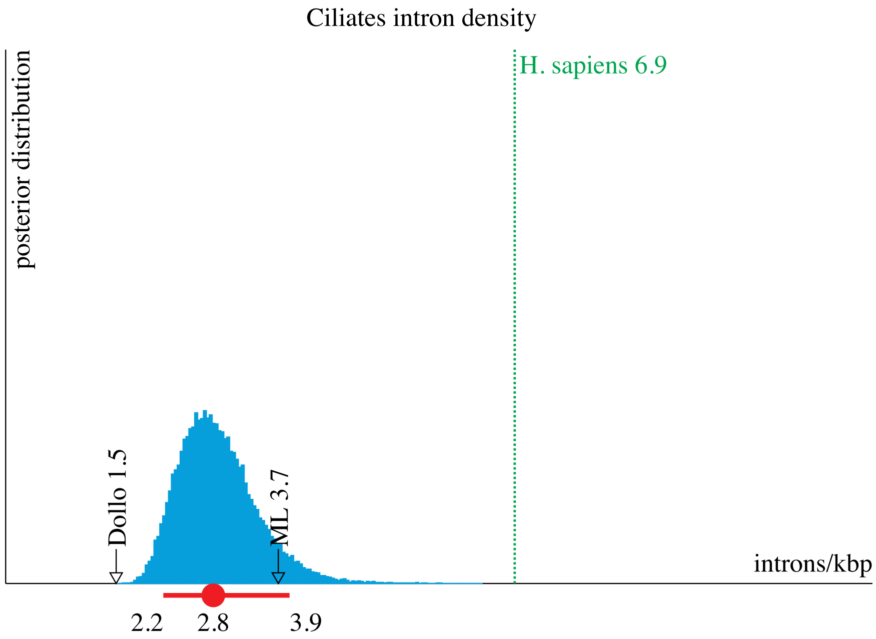

S10.xviii Cnidaria

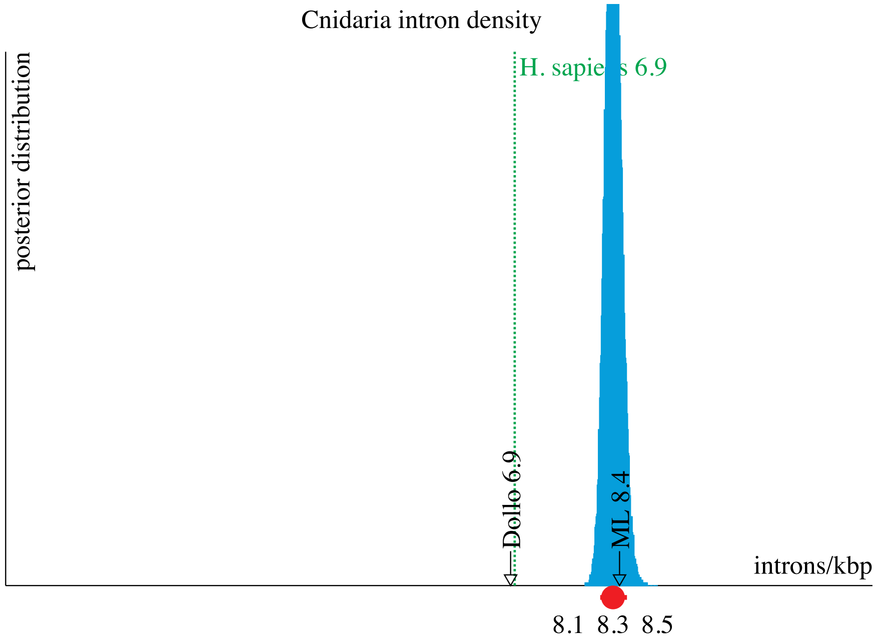

S10.xix Deuterostomes

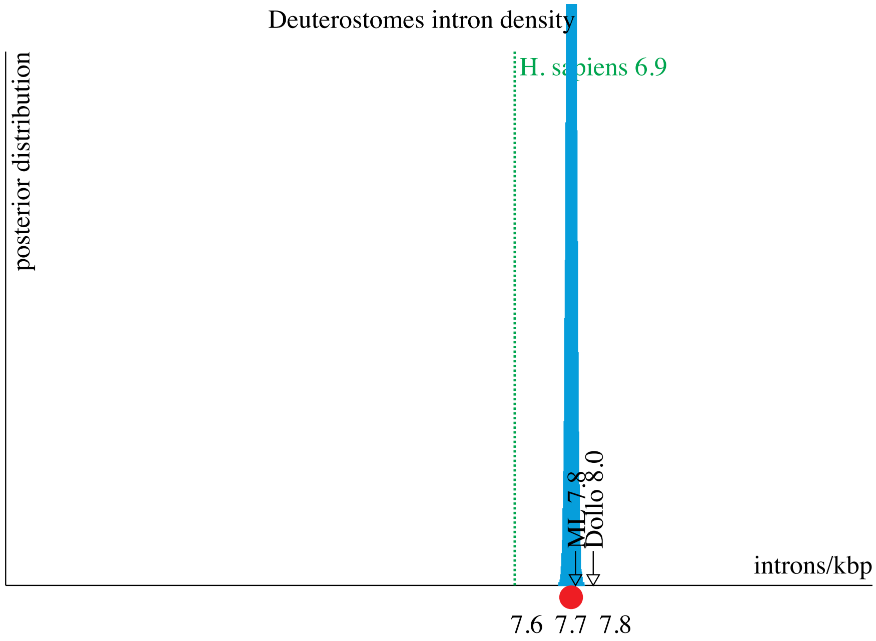

S10.xx Dikarya

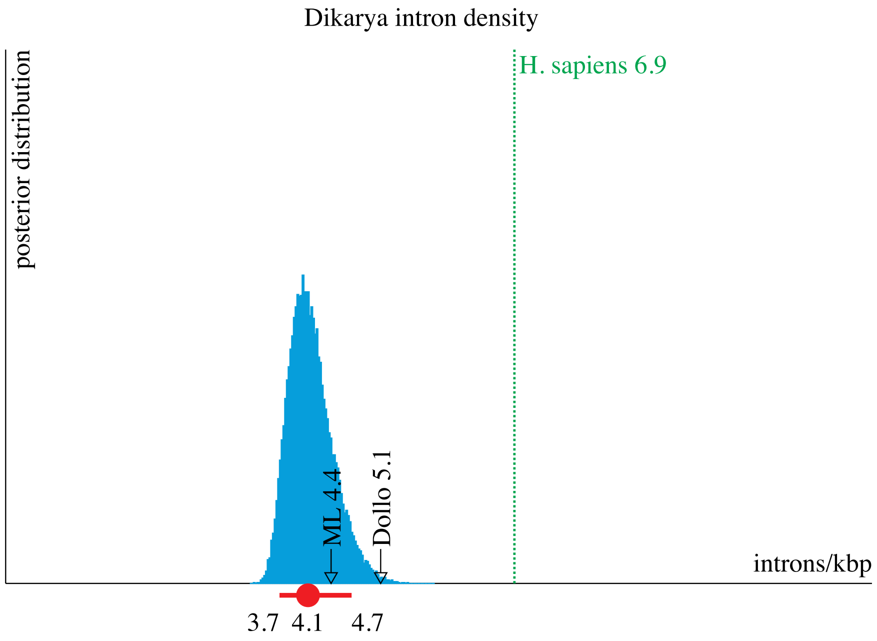

S10.xxi Diptera

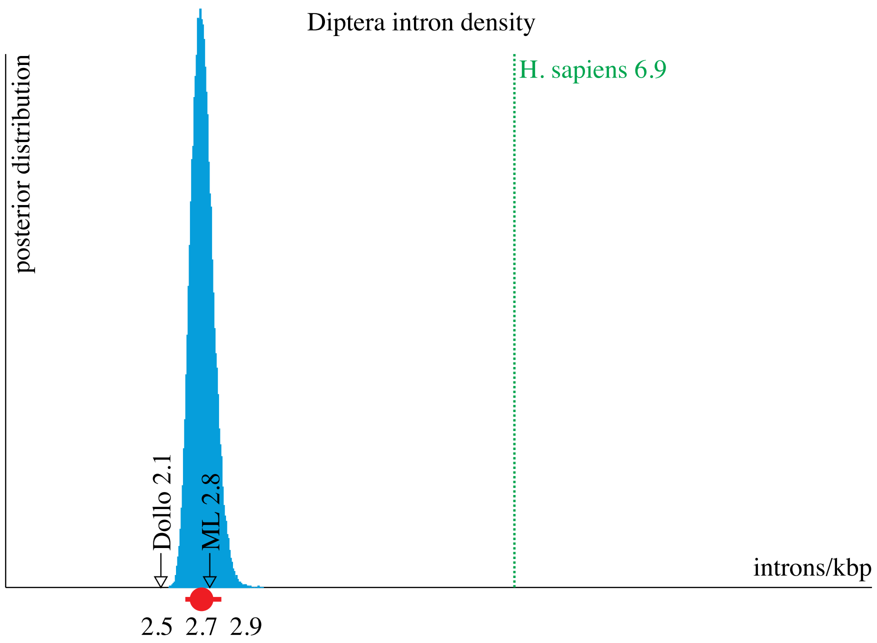

S10.xxii Dothideomycetes

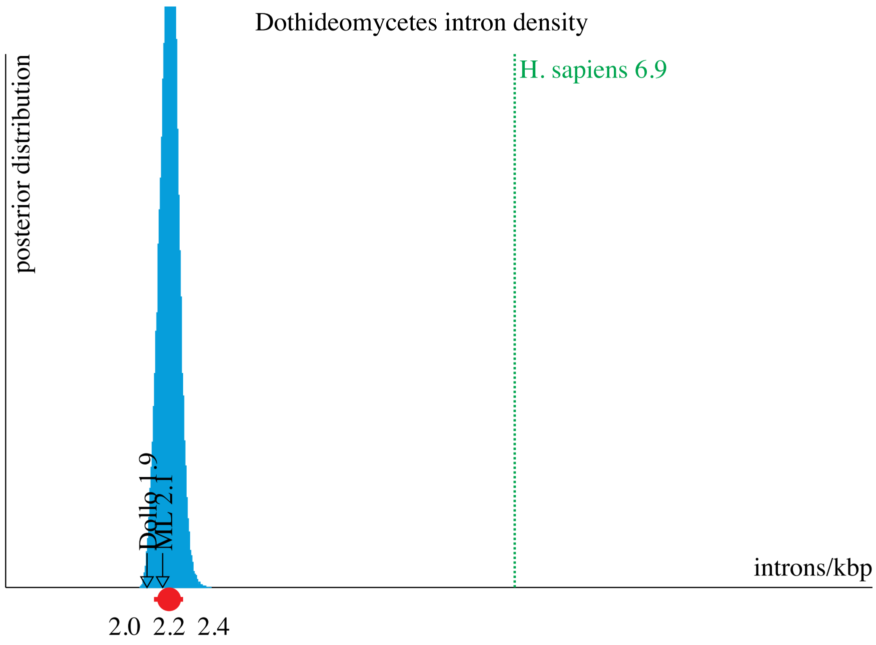

S10.xxiii Ecdysozoa

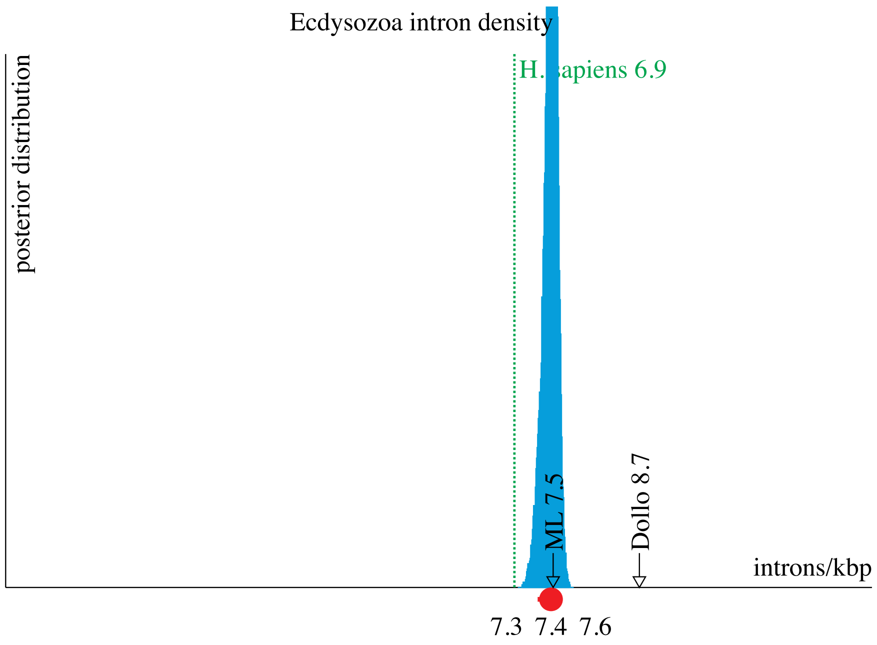

S10.xxiv Embryophyta

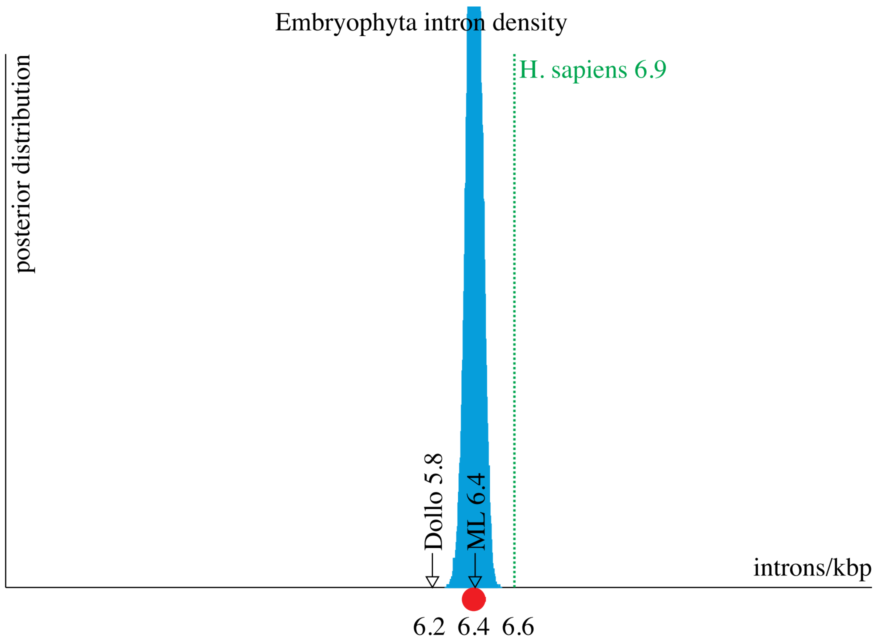

S10.xxv Euascomycota

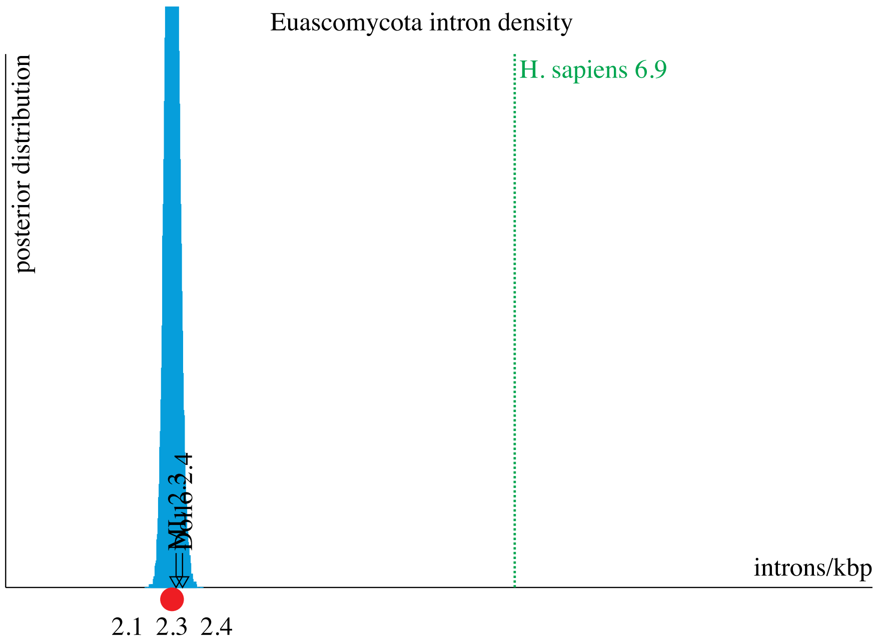

S10.xxvi eudicots

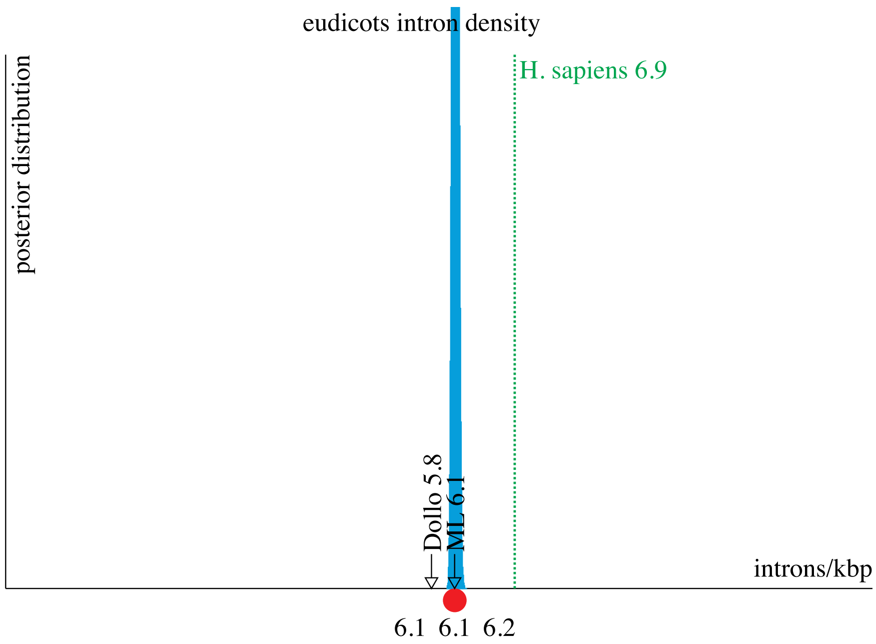

S10.xxvii Filozoa

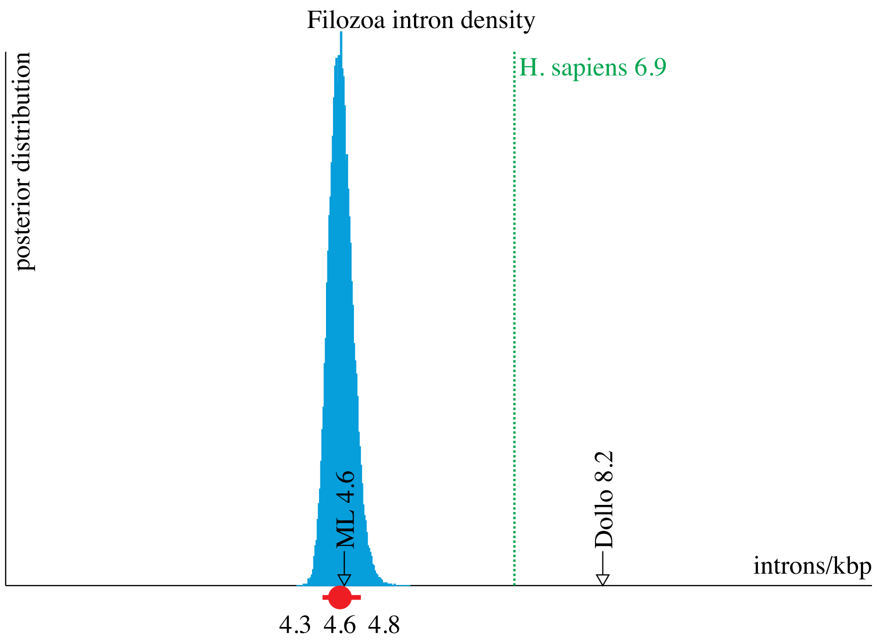

S10.xxviii Fungi

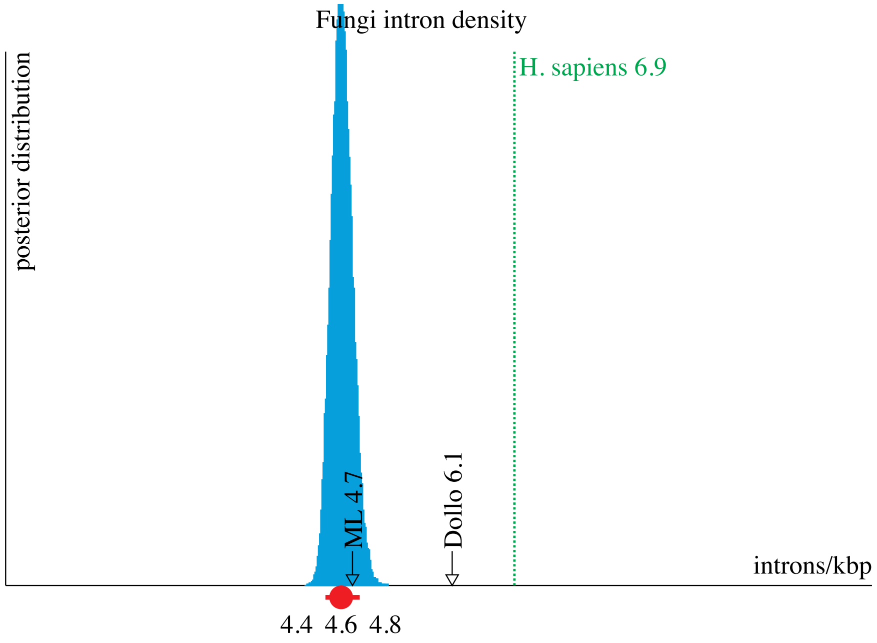

S10.xxix Hemiptera

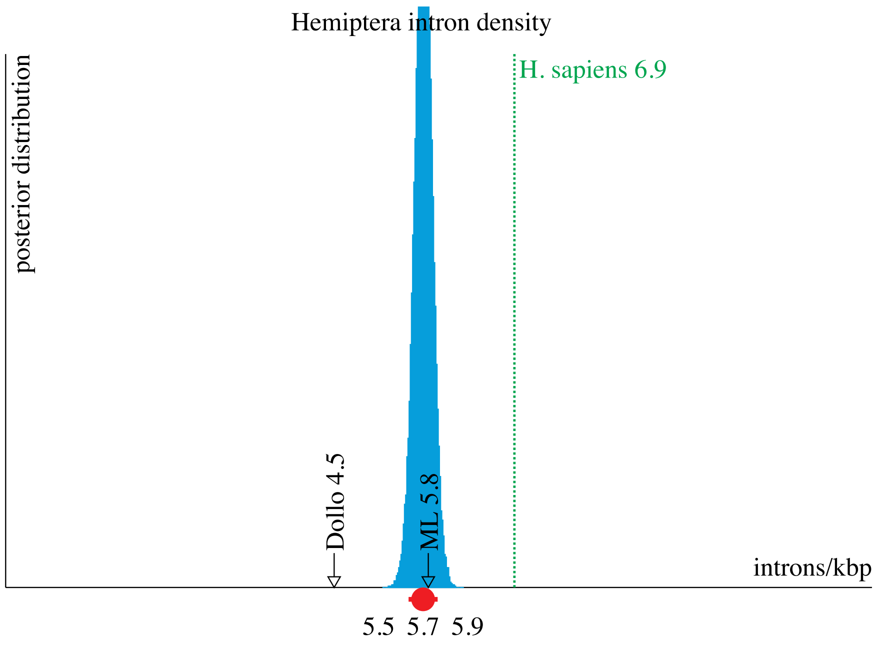

S10.xxx Heterokonts

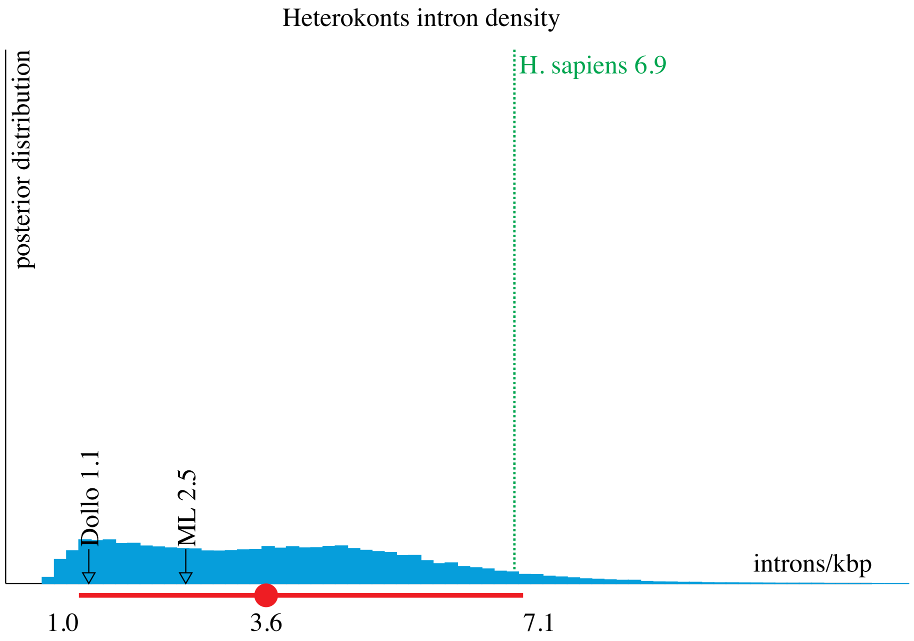

S10.xxxi Holozoa

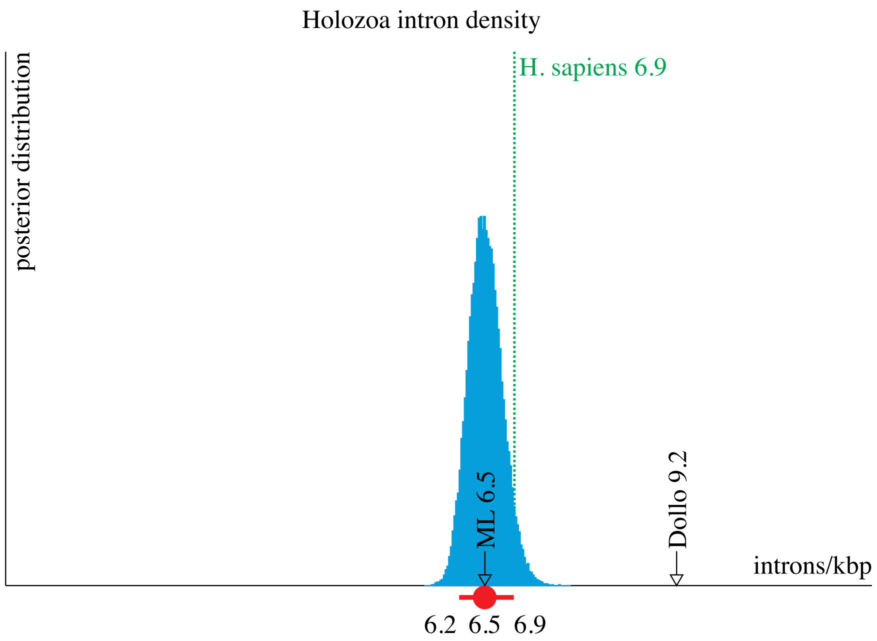

S10.xxxii Insects

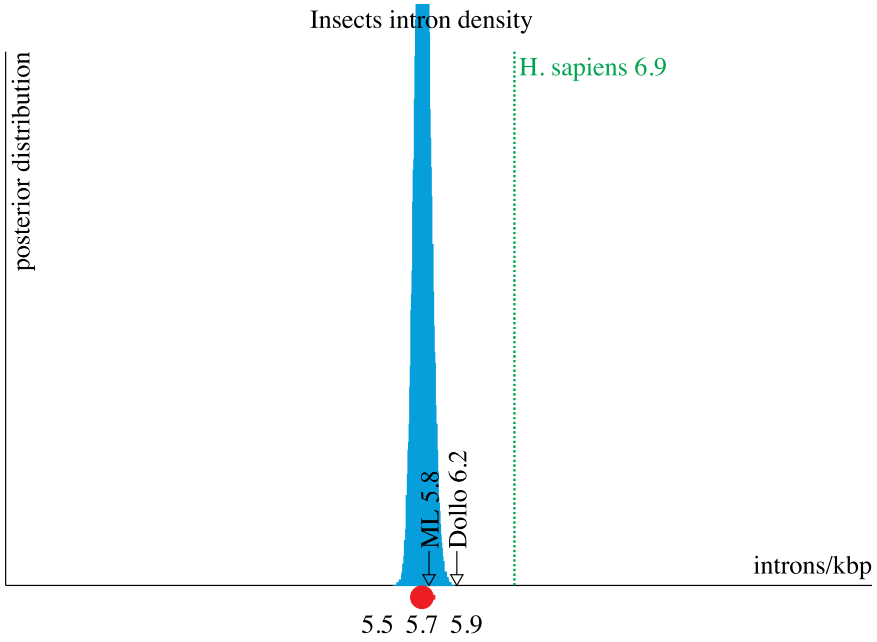

S10.xxxiii LECA

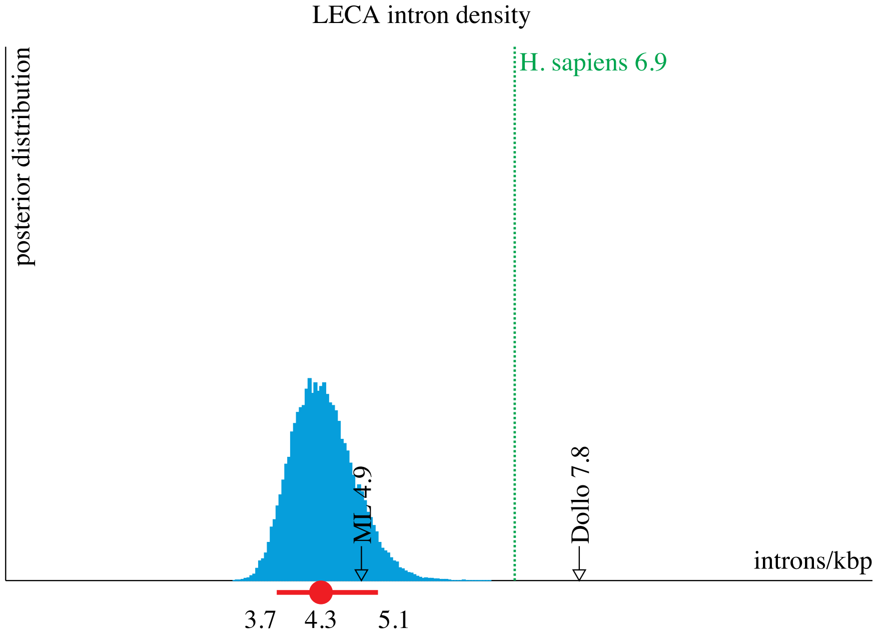

S10.xxxiv Lophotrochozoa

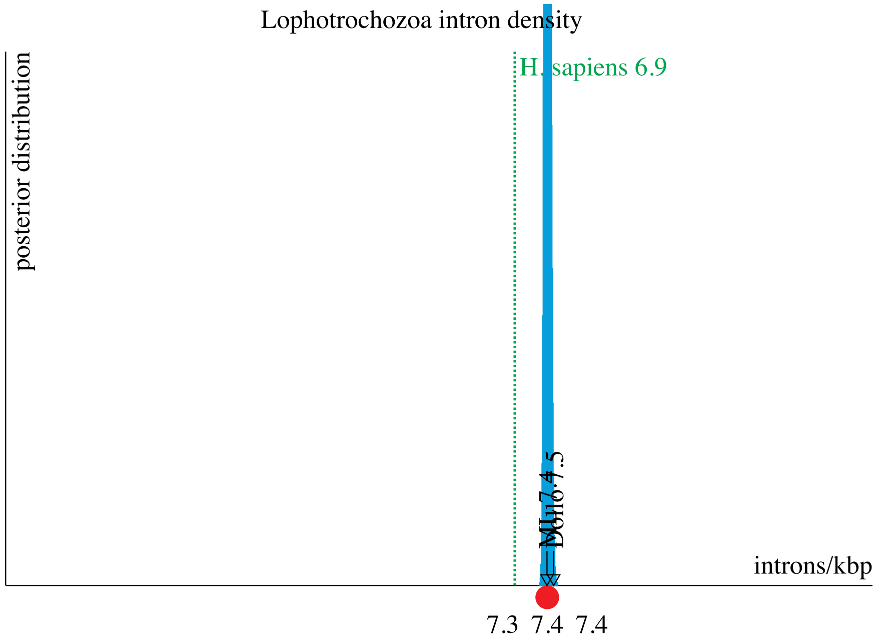

S10.xxxv Metazoa

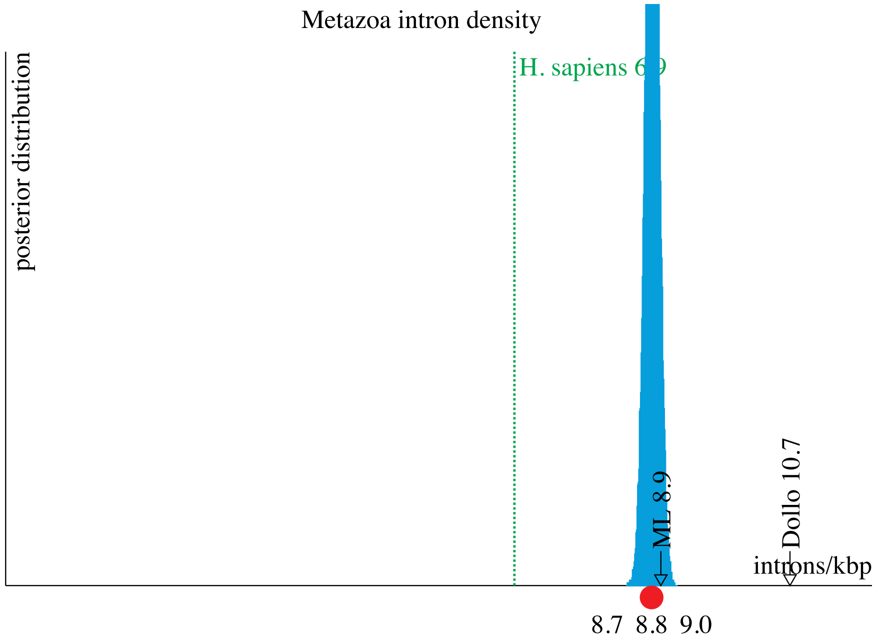

S10.xxxvi monocots

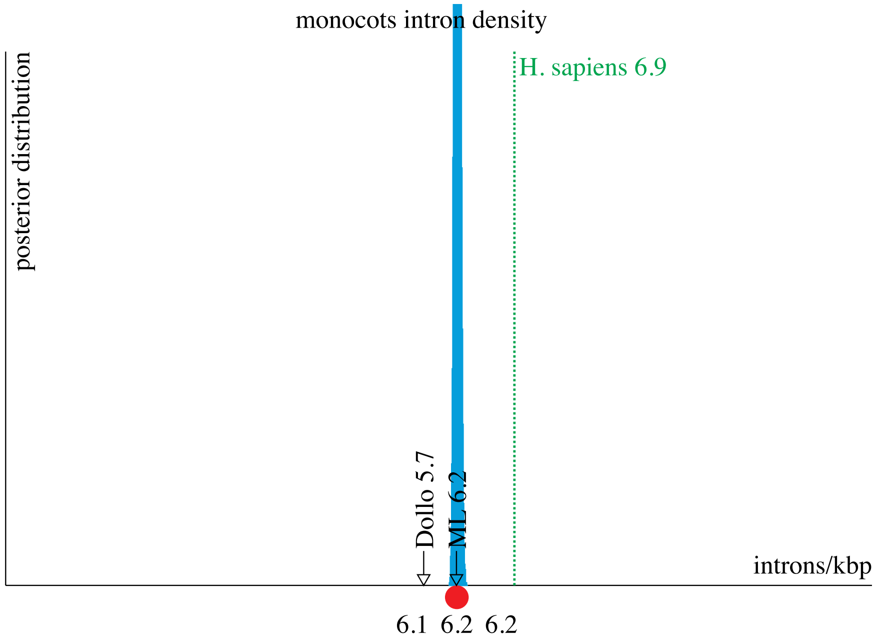

S10.xxxvii Nematodes

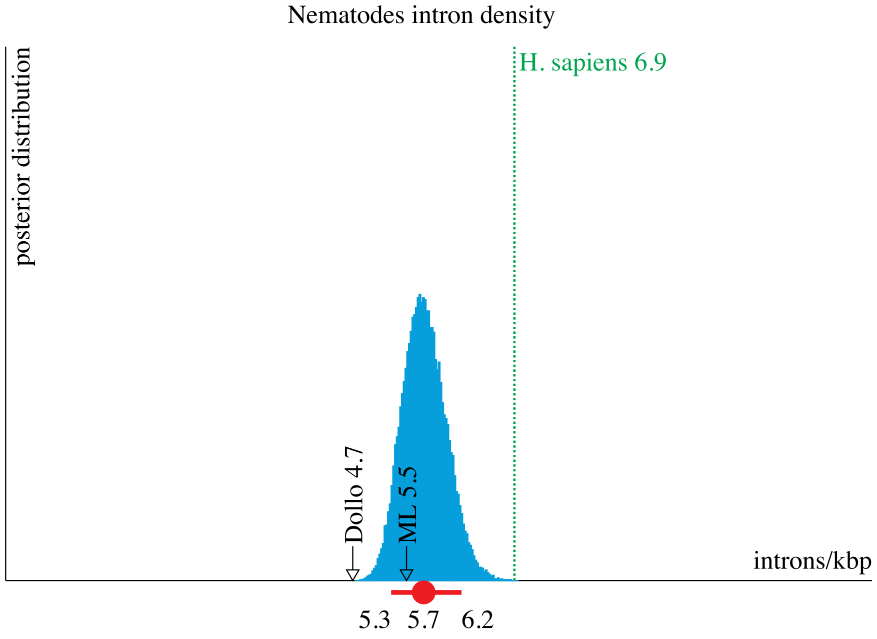

S10.xxxviii Opisthokonts

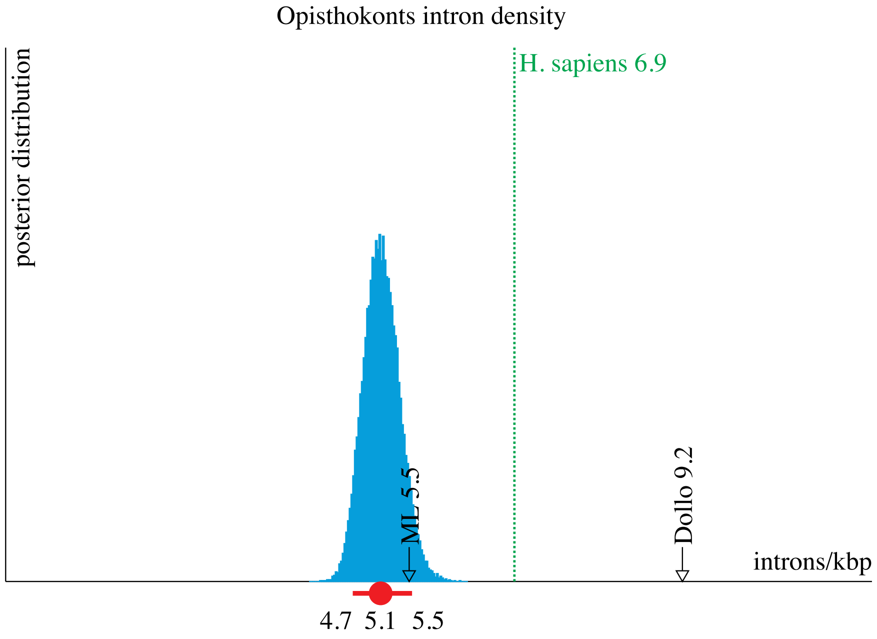

S10.xxxix Protostomes

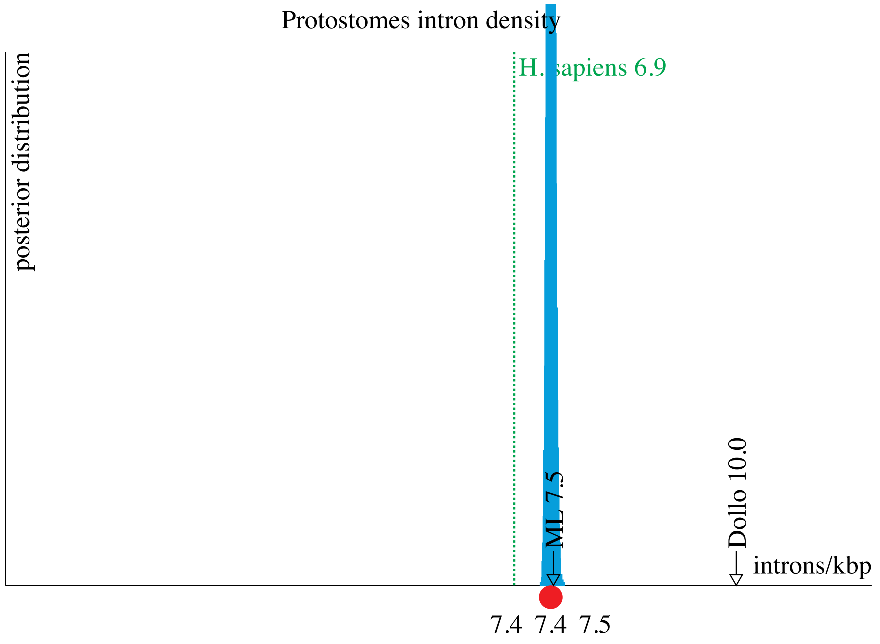

S10.xl Pucciniomycotina

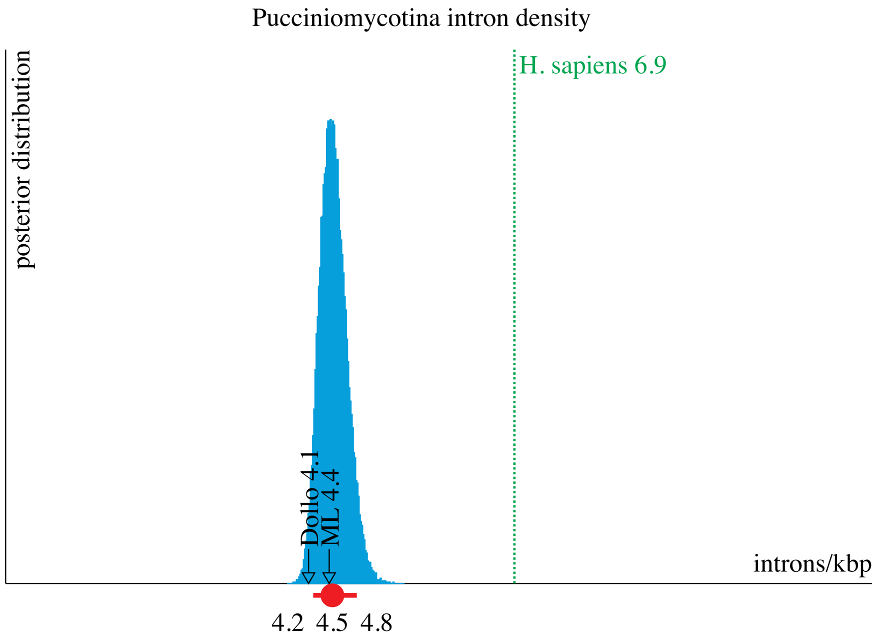

S10.xli Spiralia

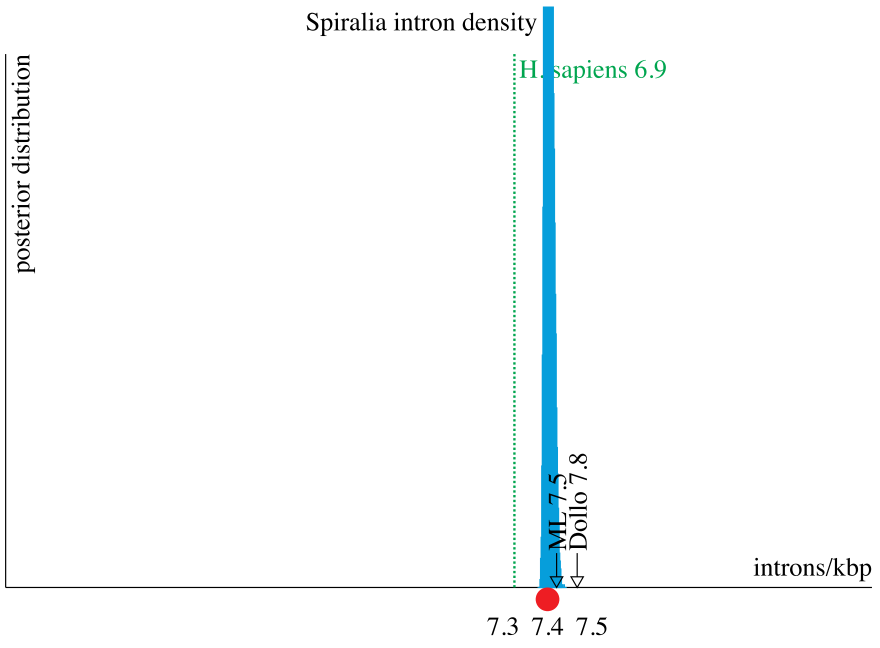

S10.xlii Tracheophyta

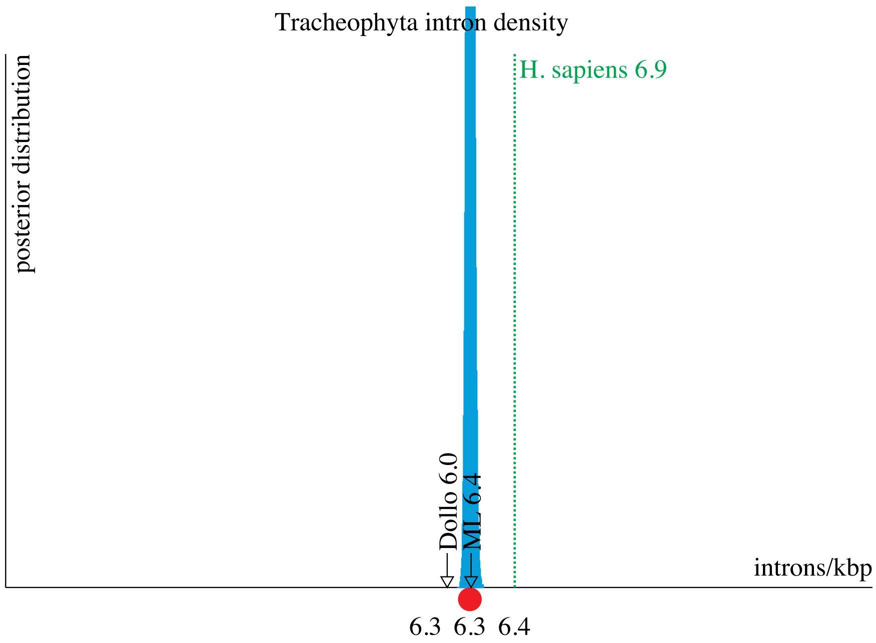

S10.xliii Unikonts

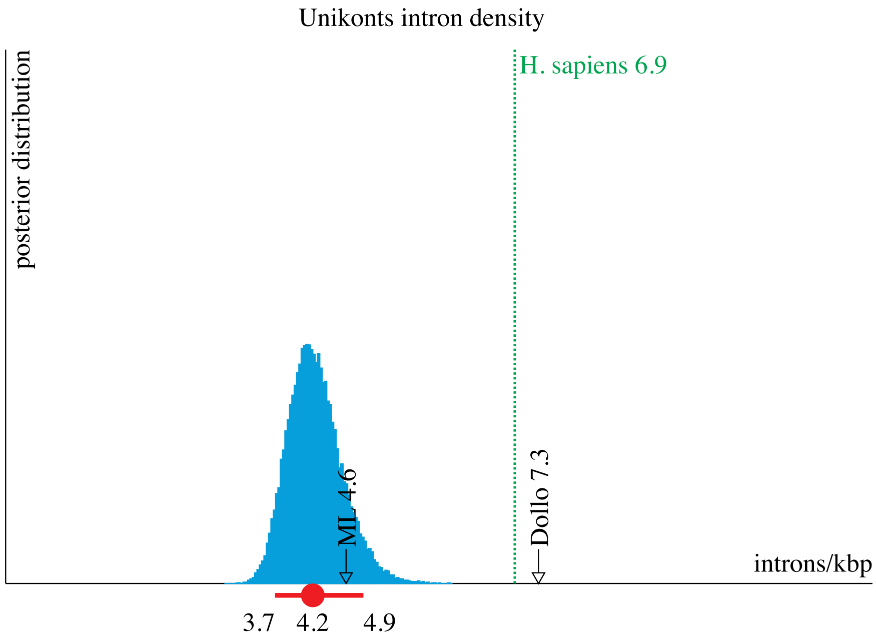

S10.xliv Vertebrates

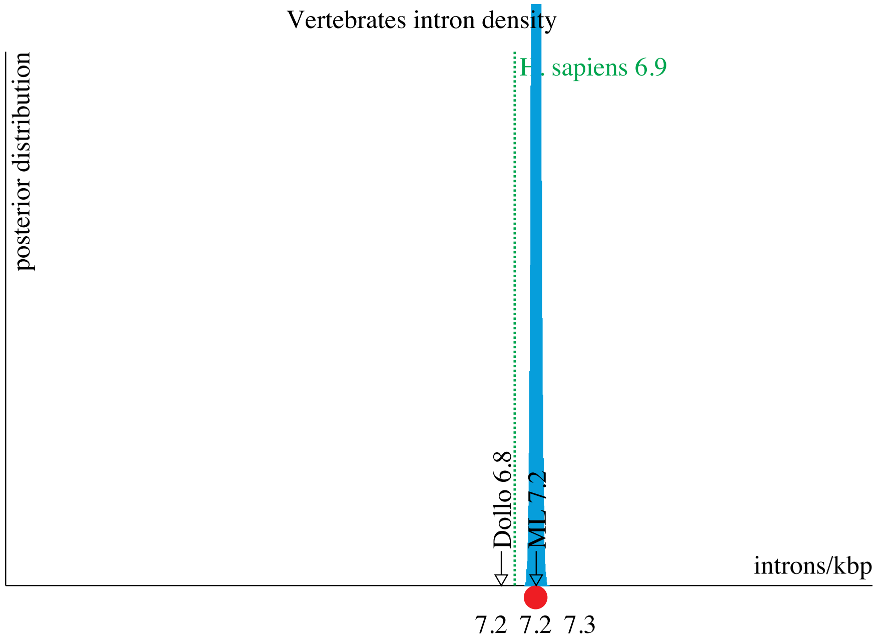

S10.xlv Archaeplastida

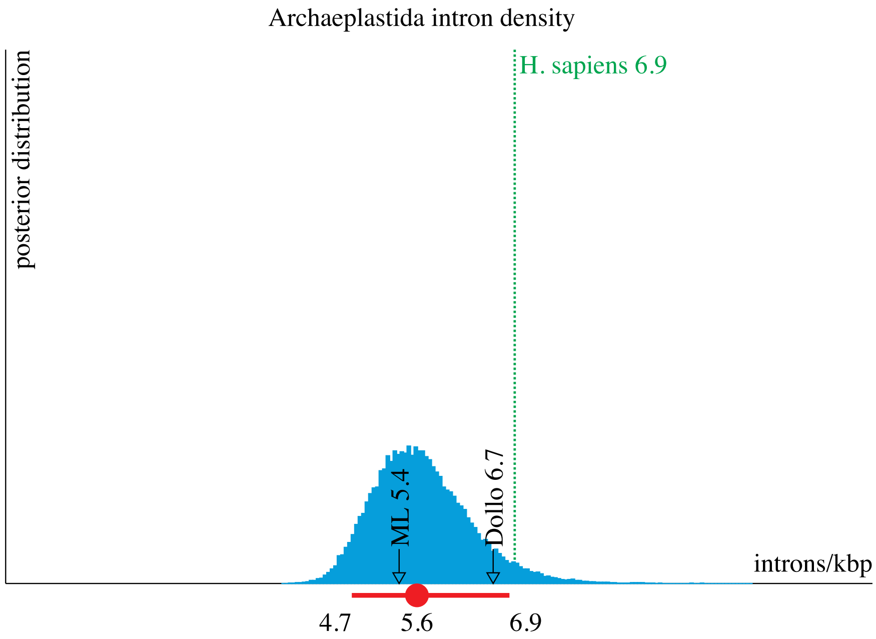

S10.xlvi Volvocales

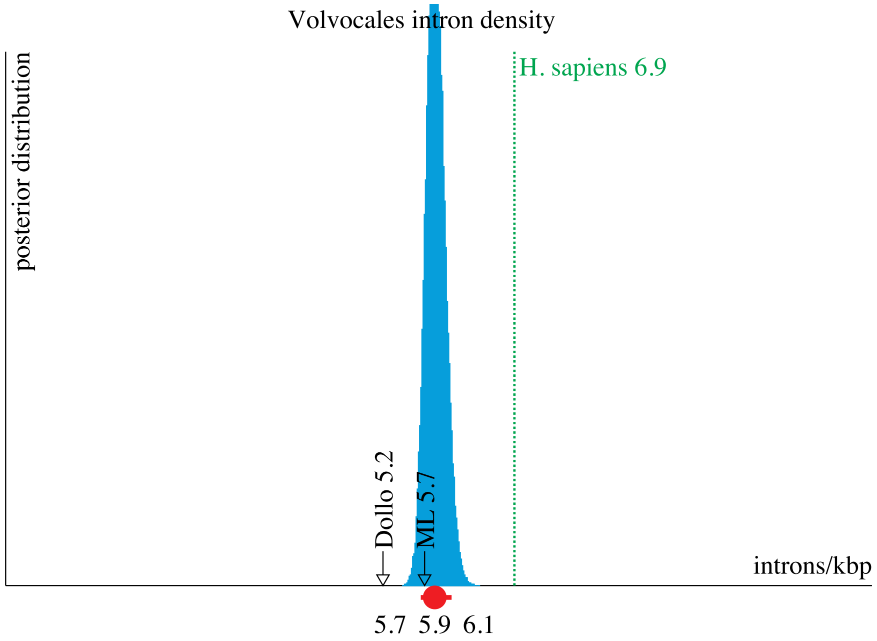

S10.xlvii xA1 (Plasmodium+Theileria+Babesia)

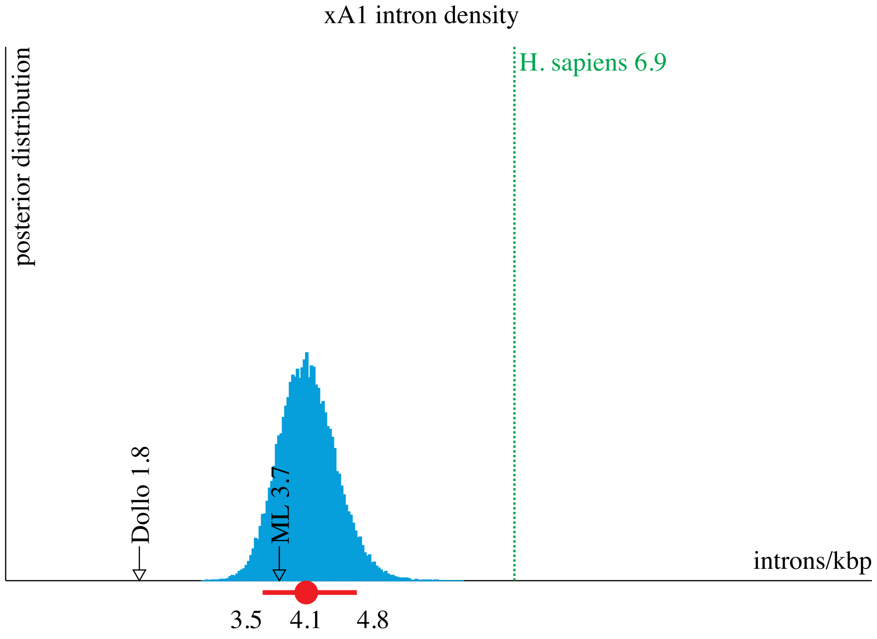

S10.xlviii xA2 (Theileria+Babesia)

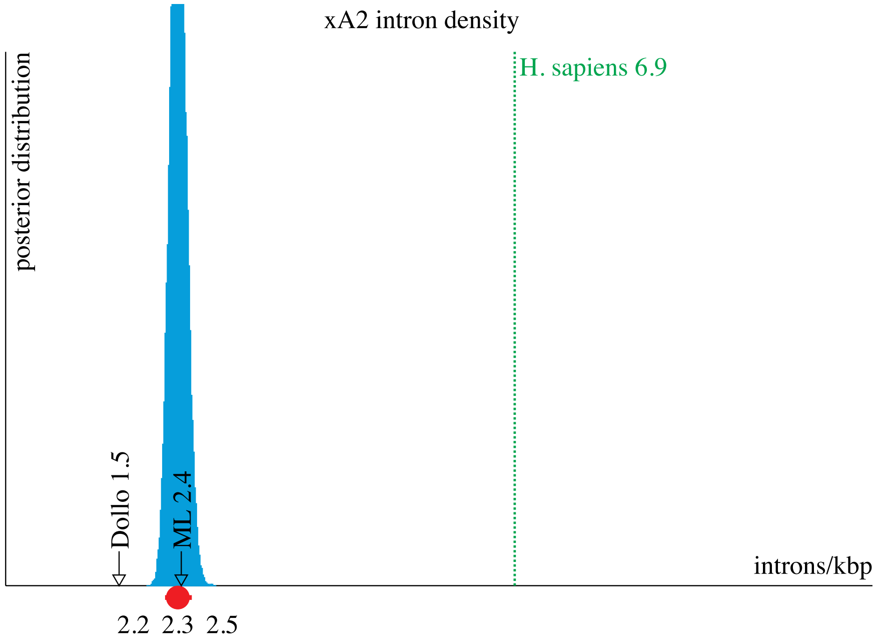

S10.xlix xF1 (sister of Chytridiomycota)

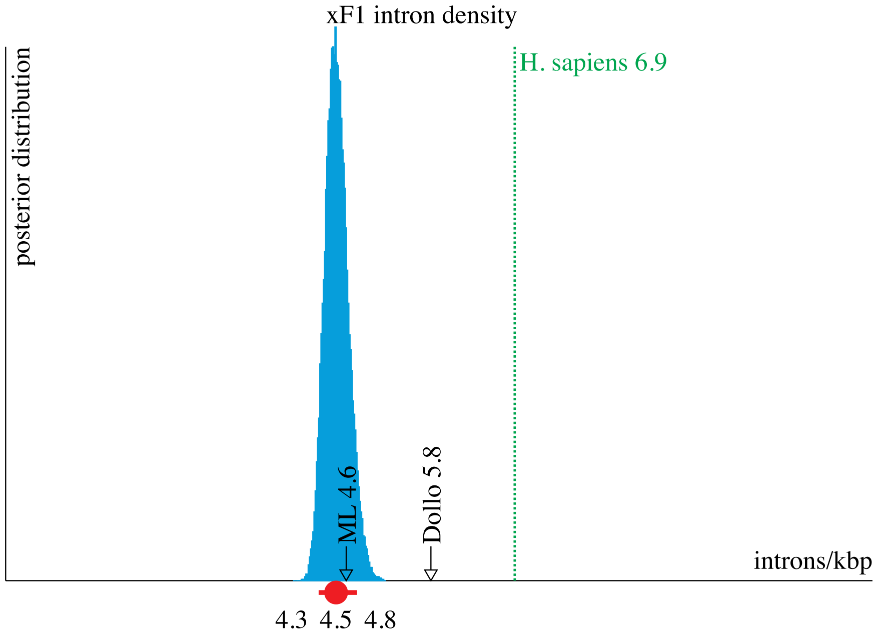

S10.l xF2

MRCA for *Allomyces macrogynus* (Chytridiomycota) and Zygomycota. Chytidiomycota are not monophyletic [Yames et al. "Reconstructing the early evolution of Fungi using a six-gene phylogeny." *Nature* 443:818–822 (2006) DOI:10.1038/nature05110].

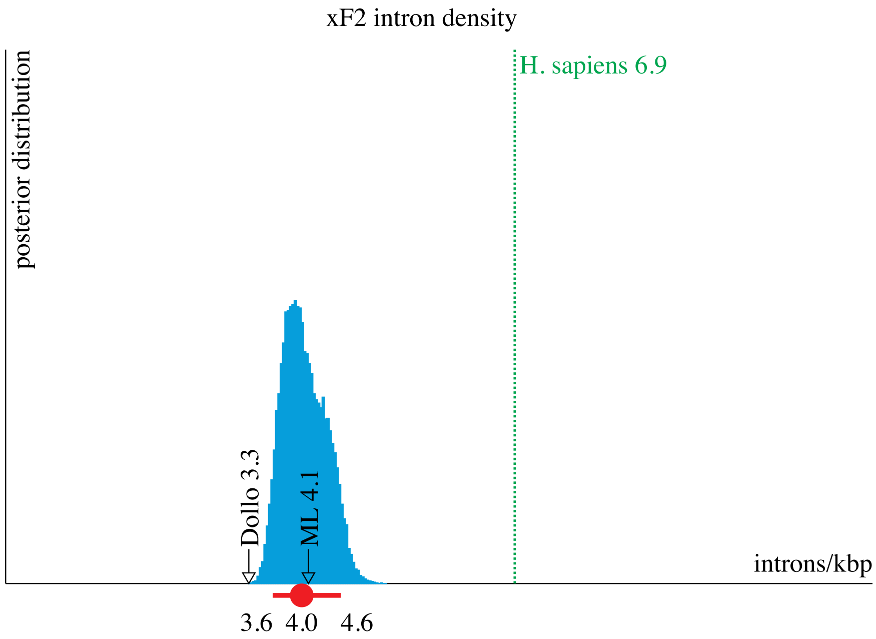

S10.li xF4 (sister of Schizosaccharomycetes)

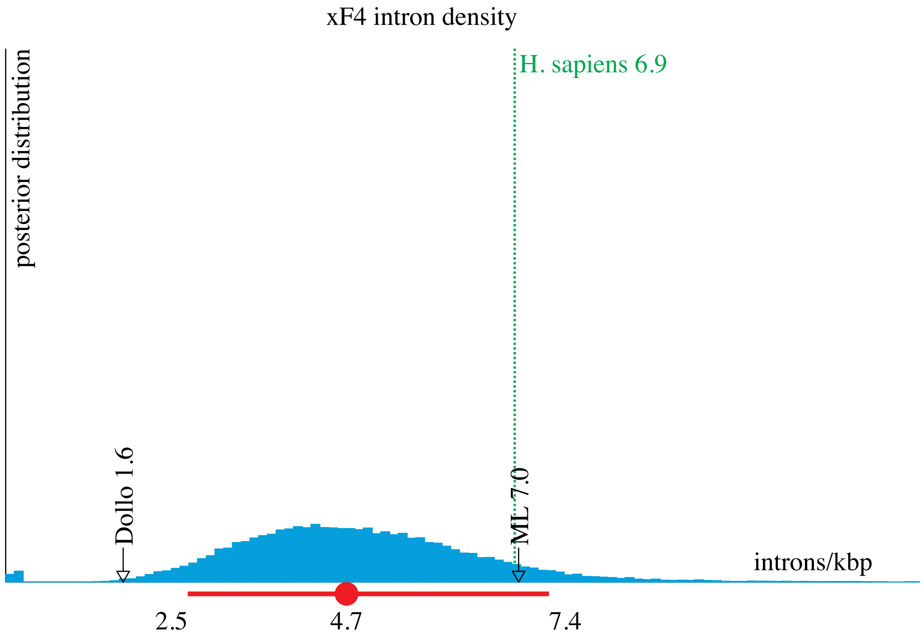

S10.lii xF10 (sister of Pucciniomycotina)

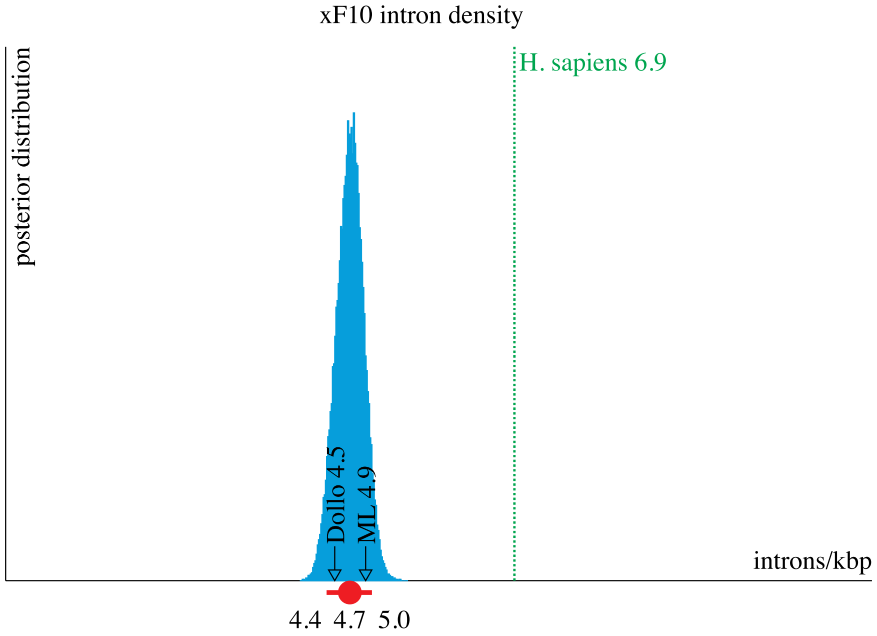

S10.liii xG1 (sister to Mamiellales)

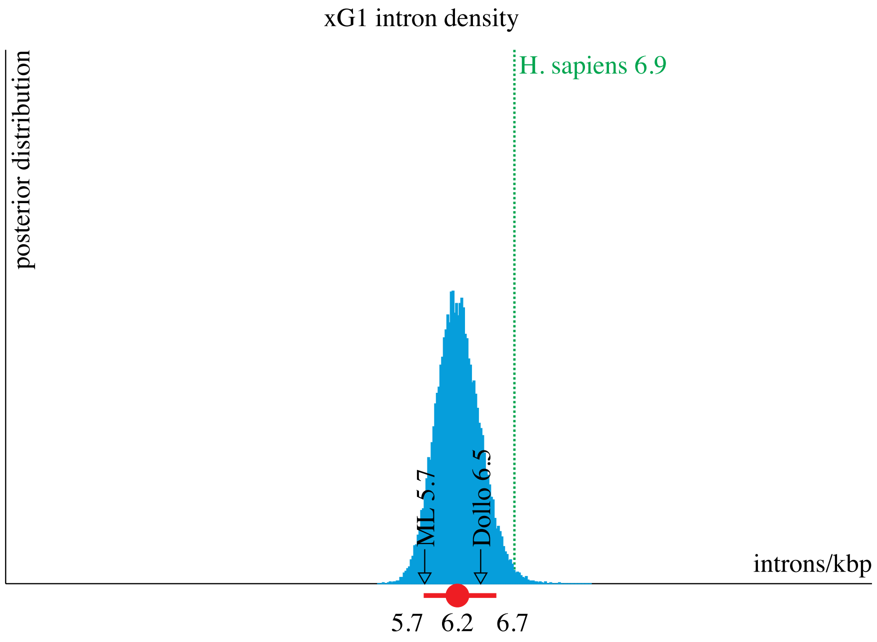

S10.liv xG2 (sister to C169)

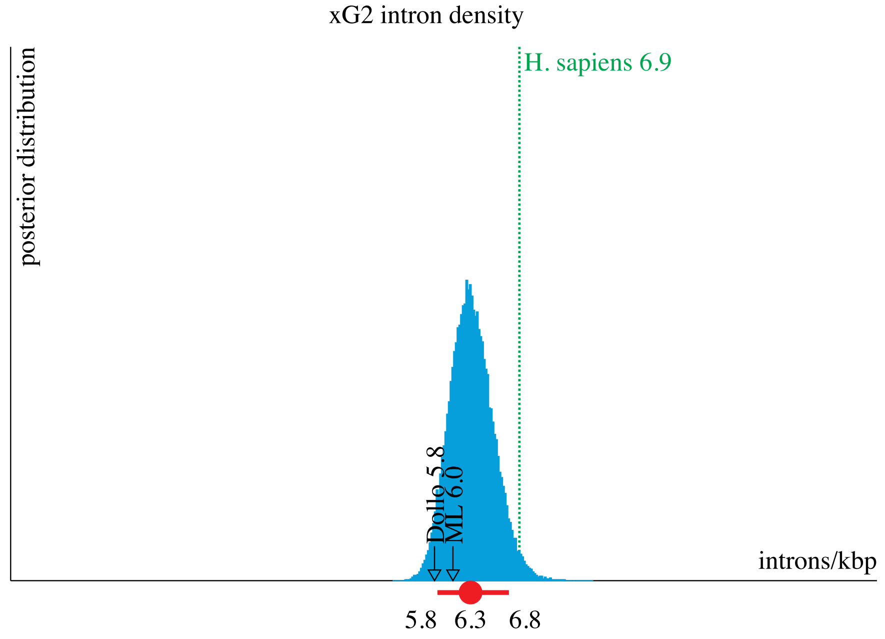

S10.lv xH1 (sister to Phytophthora)

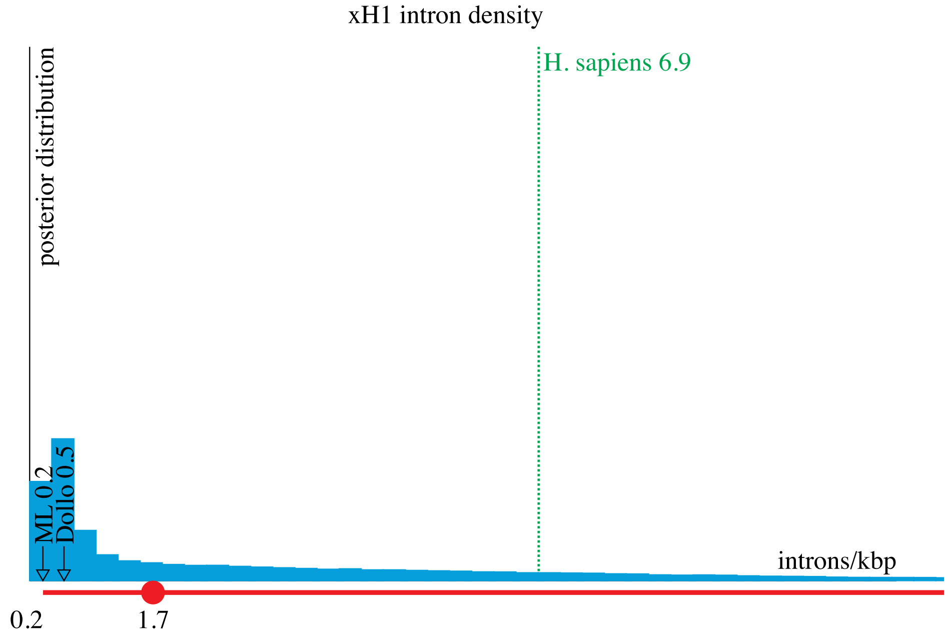

S10.lvi xM1 (sister to Trichoplax)

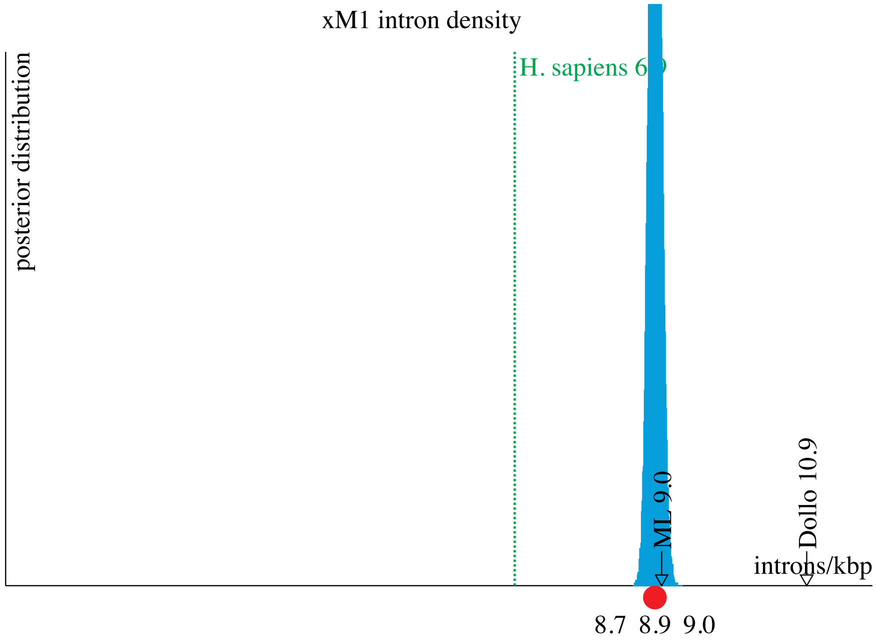

S10.lvii xM2 (insects+Daphnia)

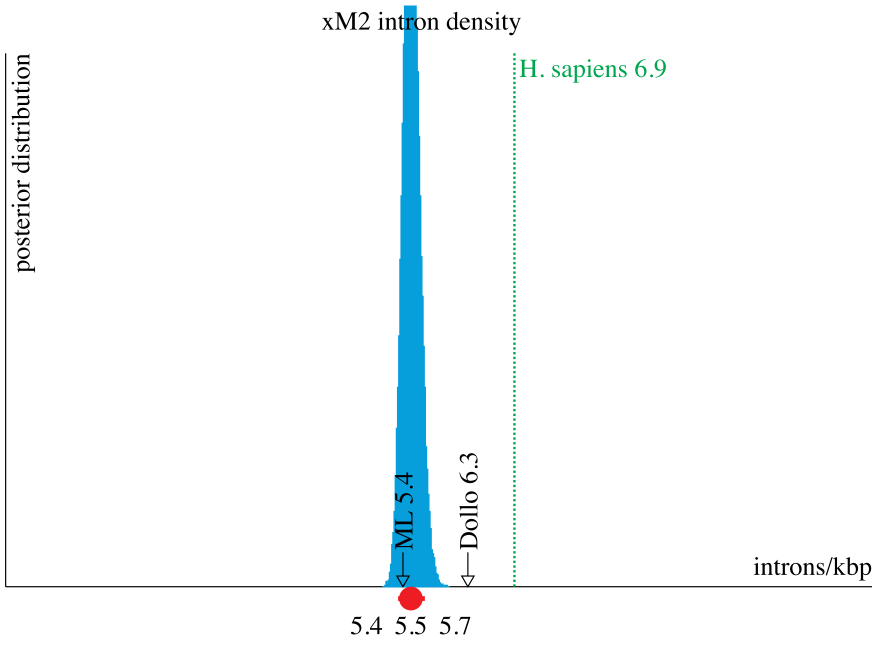

S10.lviii xM3 (sister to Hemiptera)

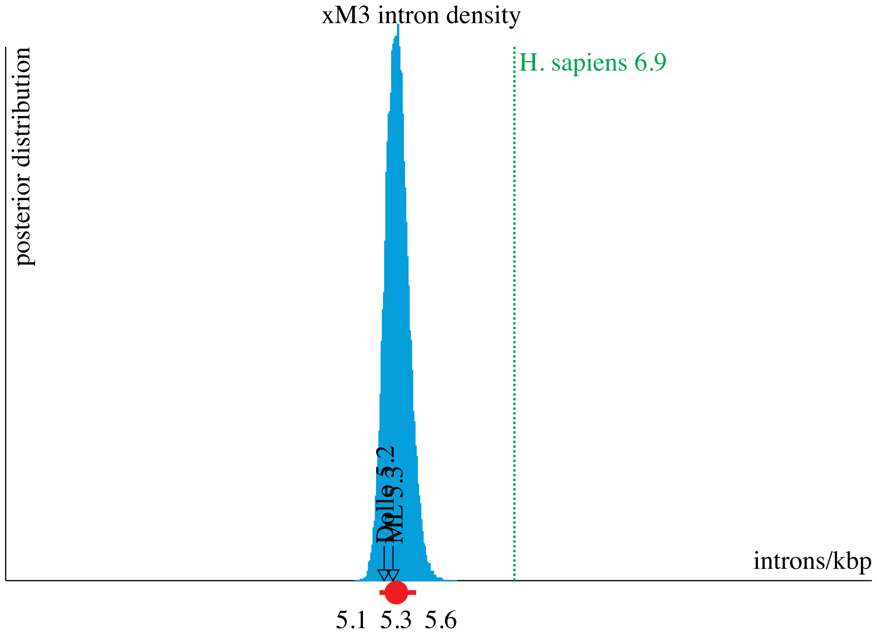

S10.lix xM4 (sister to Tcas [Coleoptera])

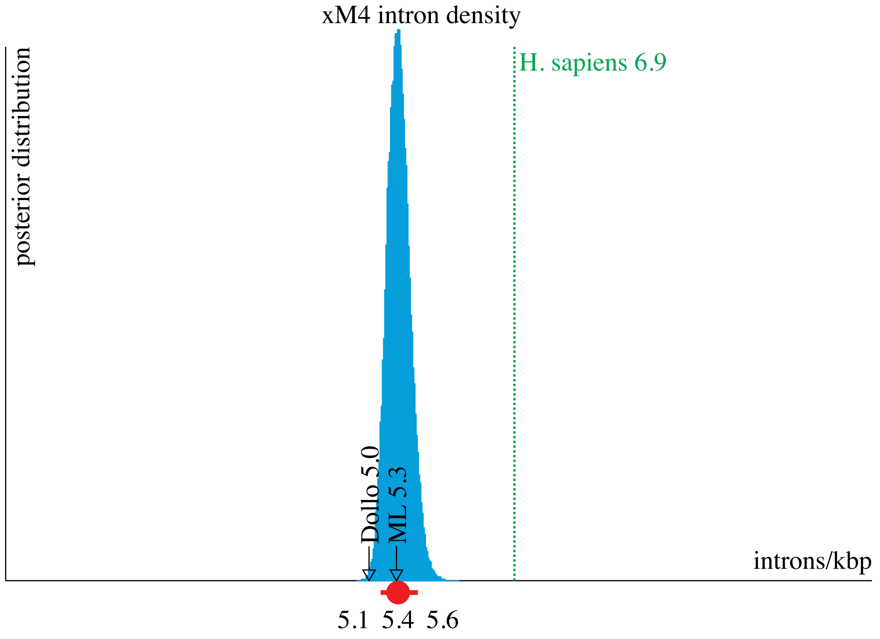

S10.lx xM5 (sister to Hymenoptera)

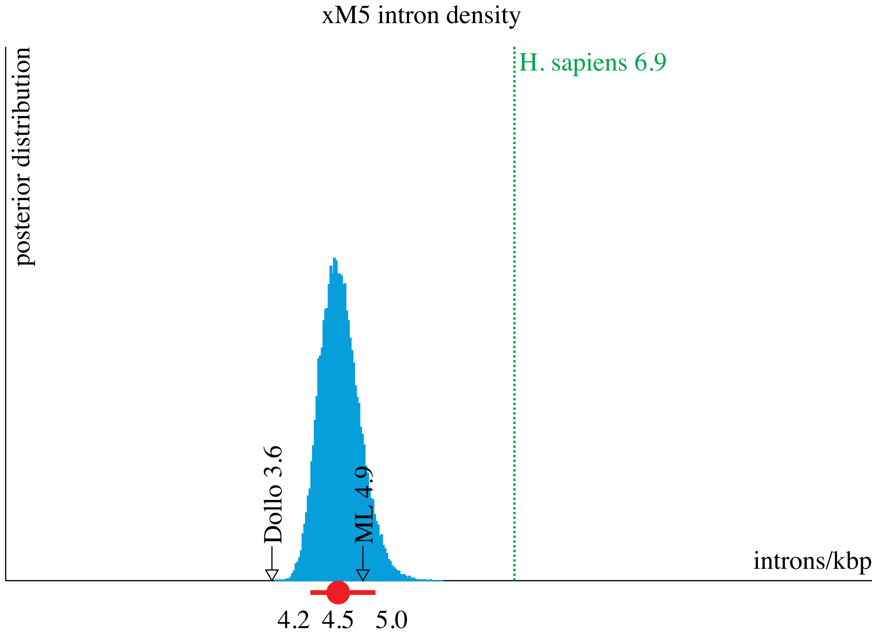

S10.lxi xM6

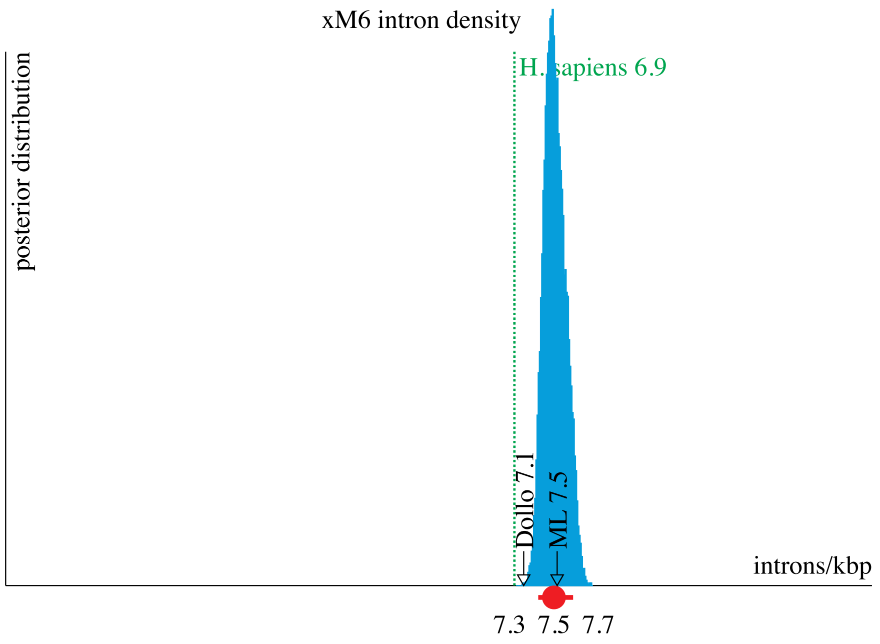

S10.lxii Zygomycota

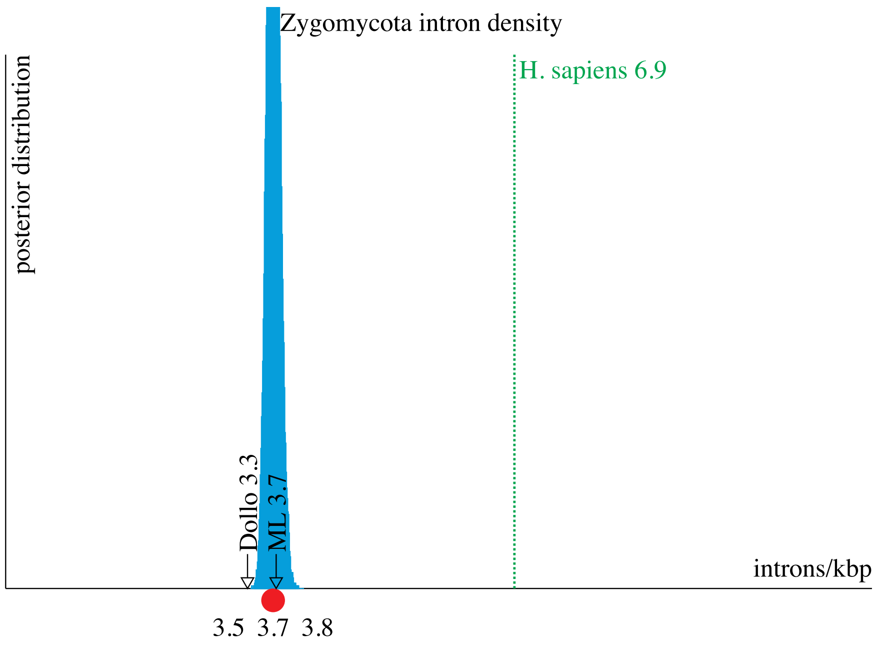

Very little uncertainty

S10.lxiii Agaricales

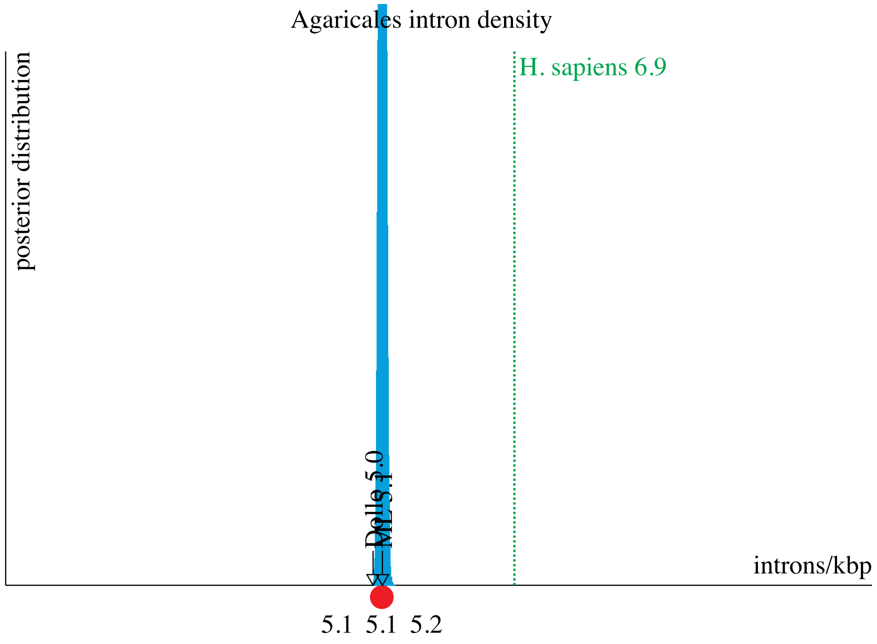

S10.lxiv Amniota

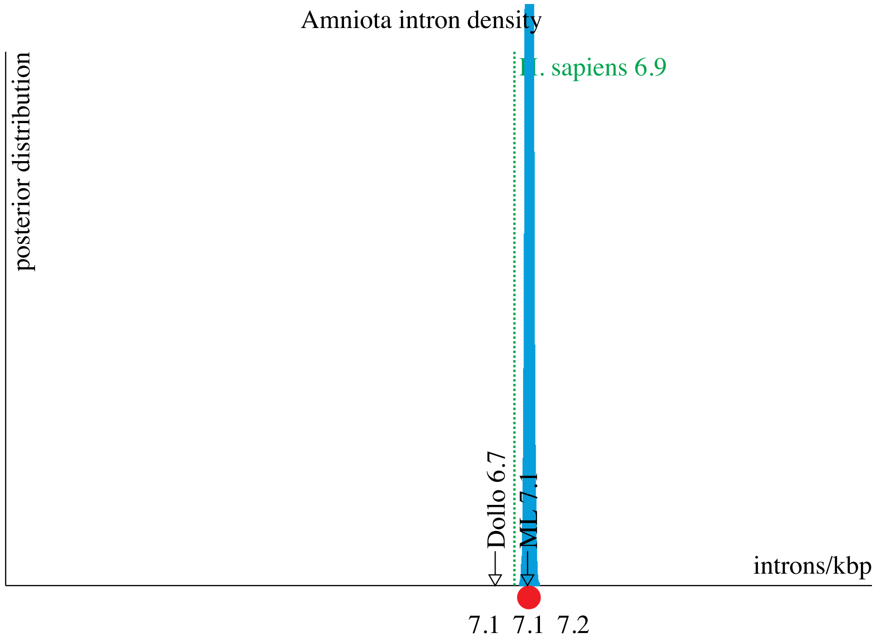

S10.lxv Aves

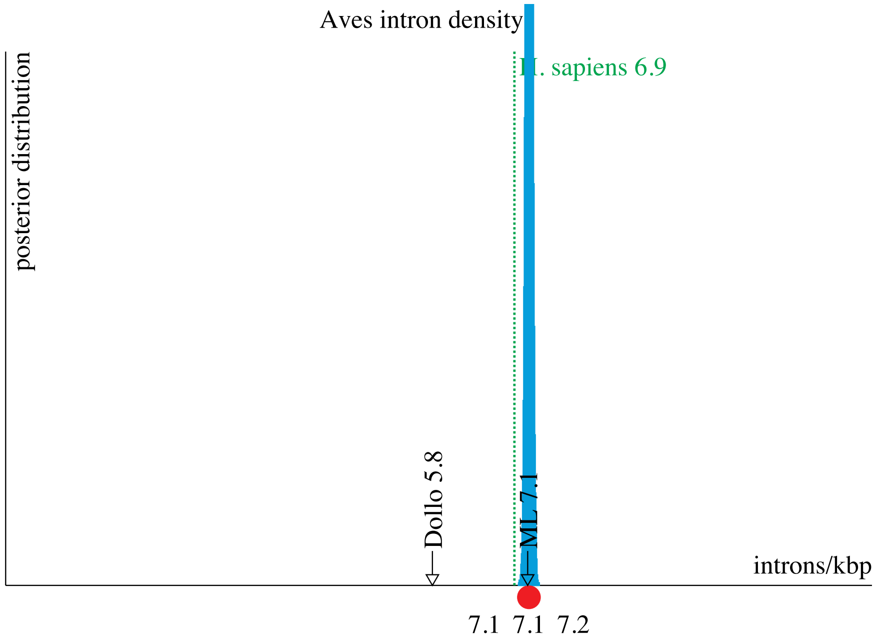

S10.lxvi Bacillariophycaea

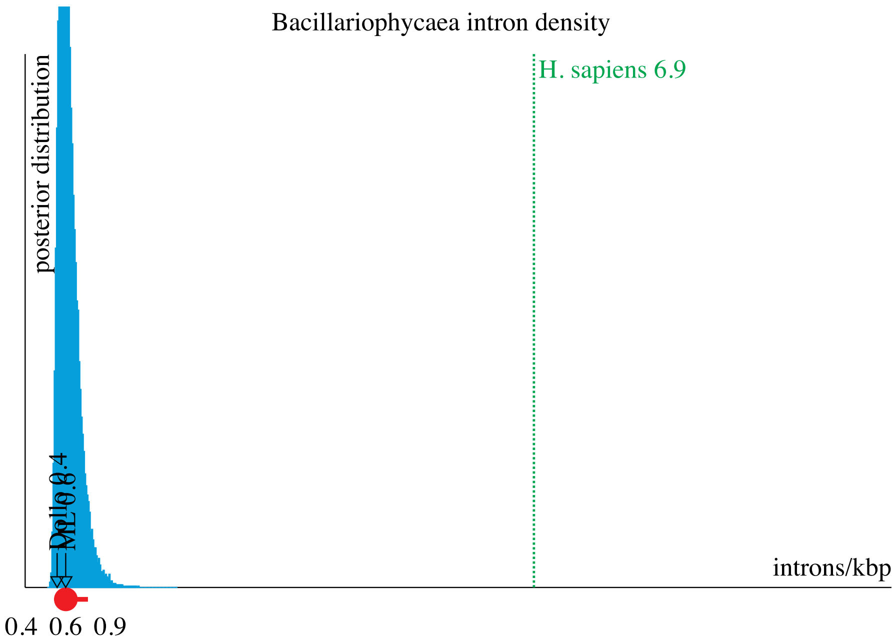

S10.lxvii Caenorhabditis

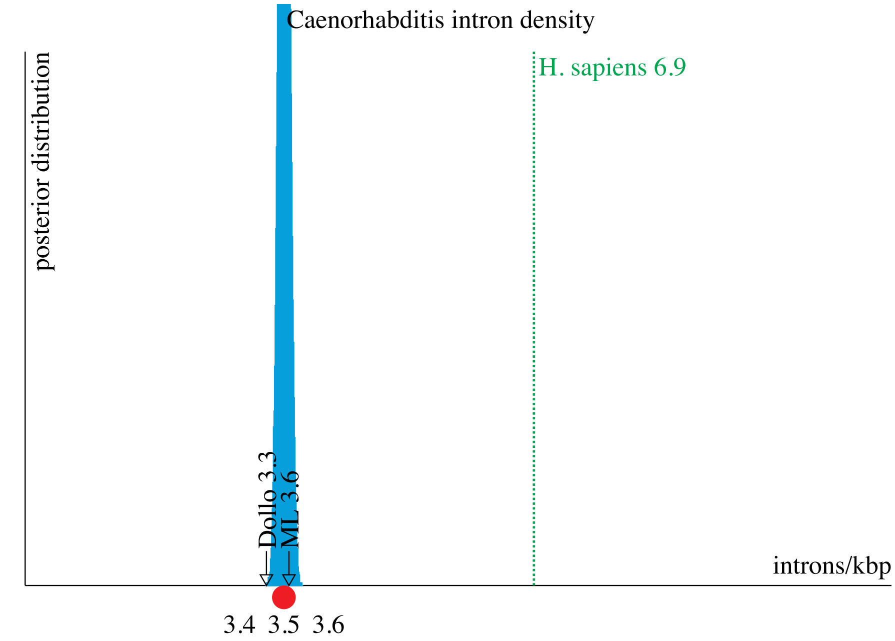

S10.lxviii Culicidae

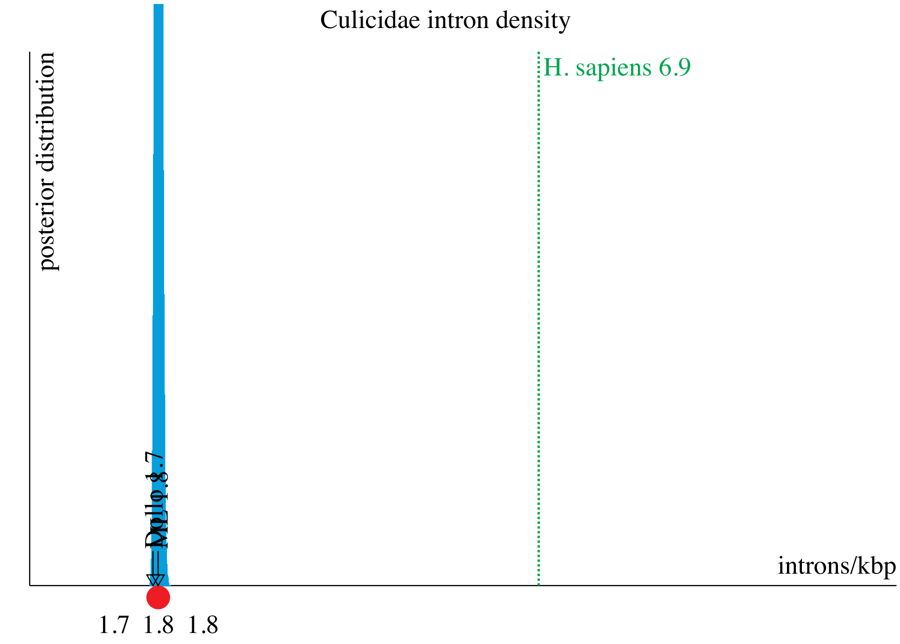

S10.lxix diatoms

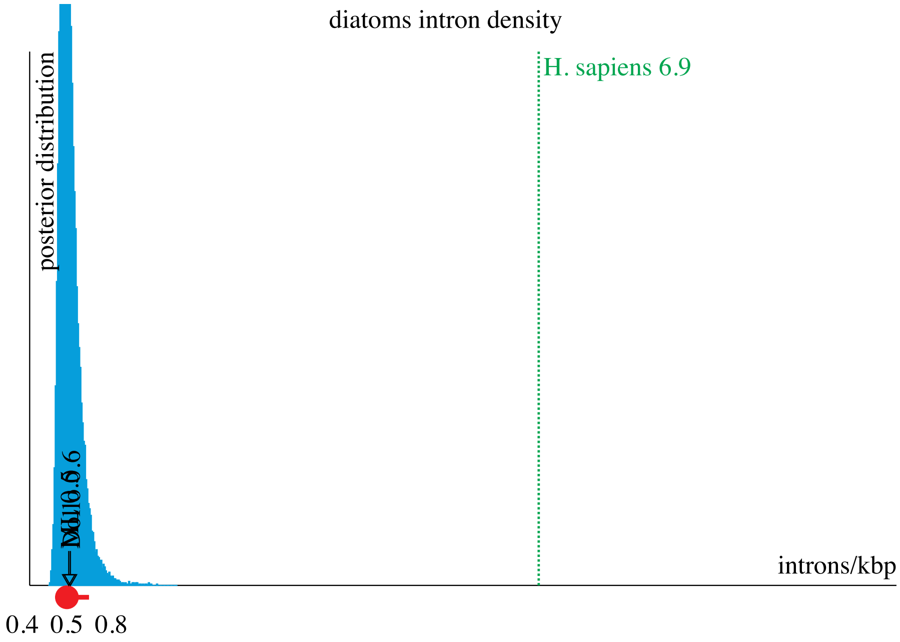

S10.lxx Dictyostelium

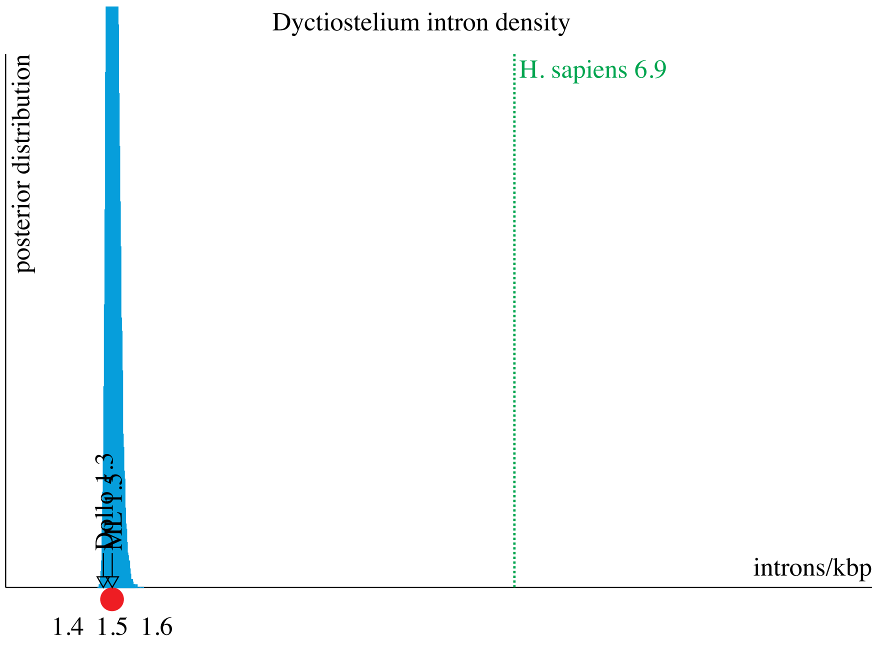

S10.lxxi Drosophilidae

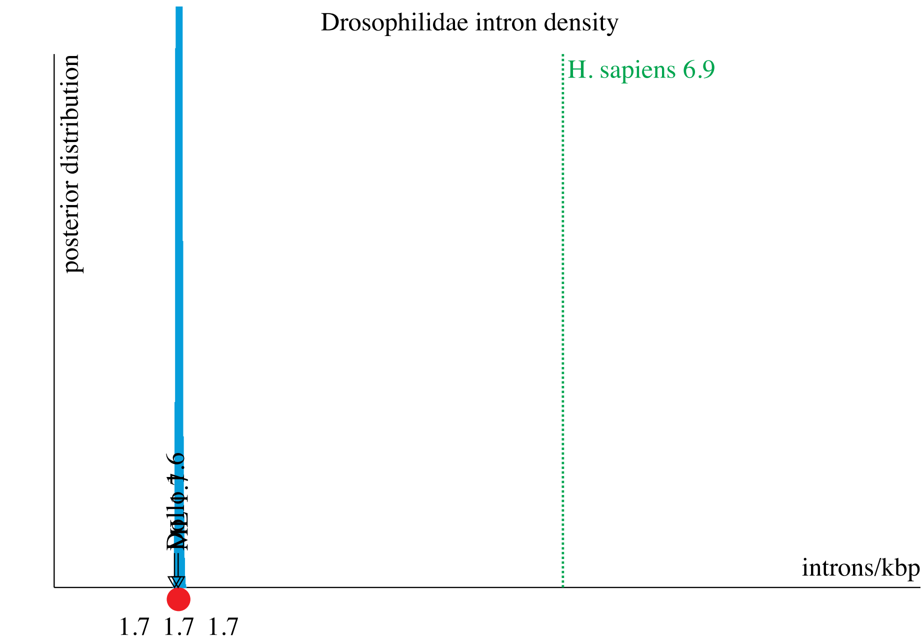

S10.lxxii Entamoeba

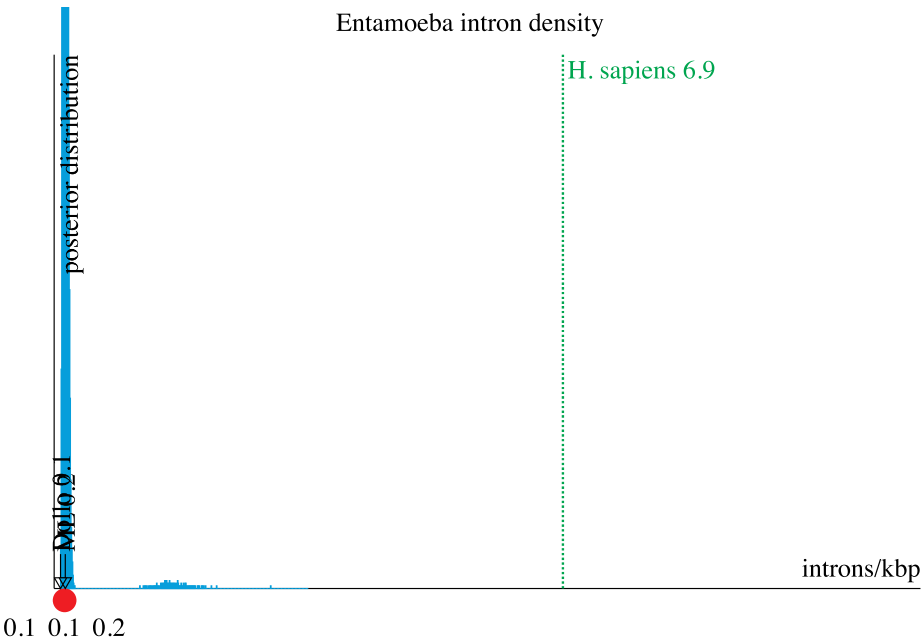

S10.lxxiii Eurotiomycetes

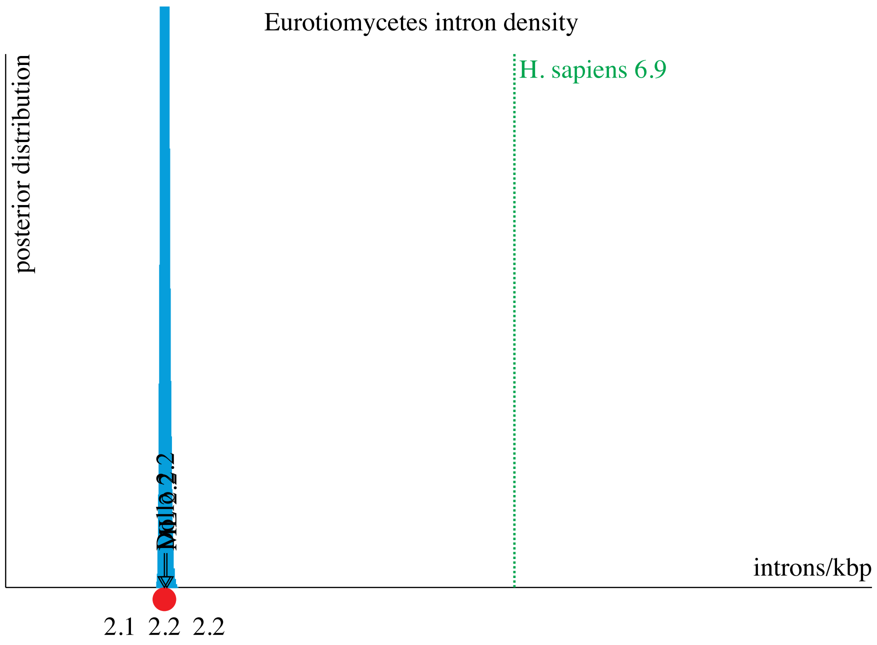

S10.lxxiv Hymenoptera

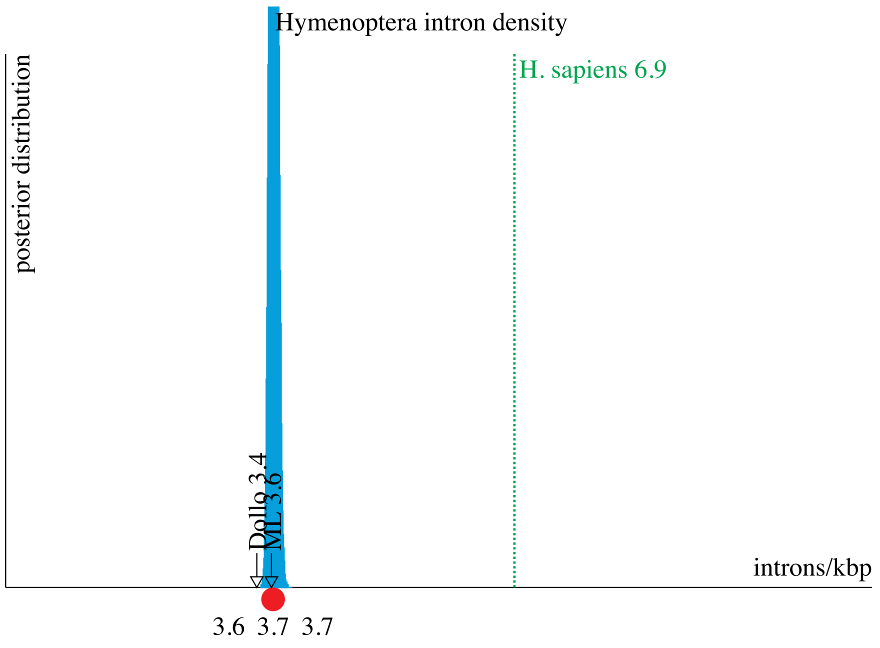

S10.lxxv Leotiomycetes

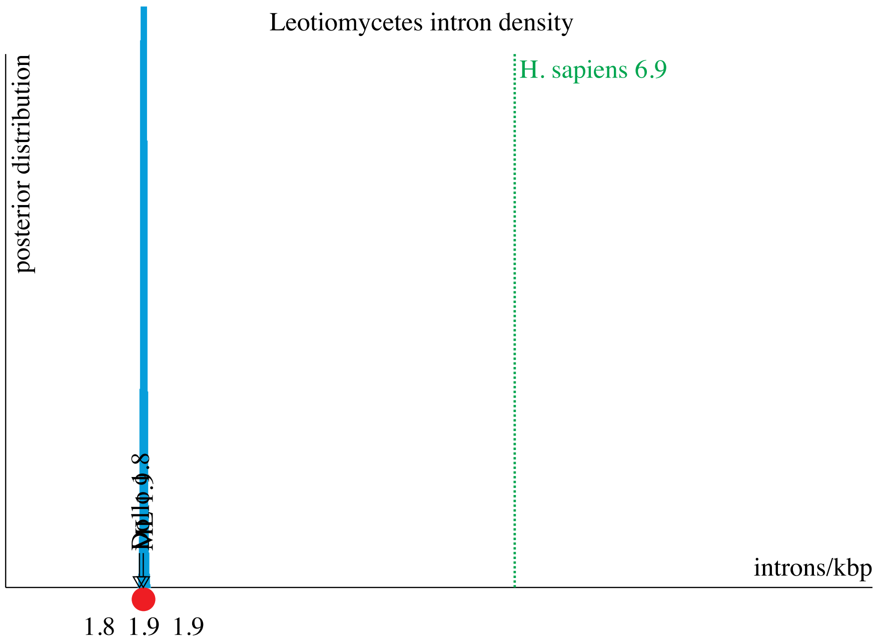

S10.lxxvi Mamiellales

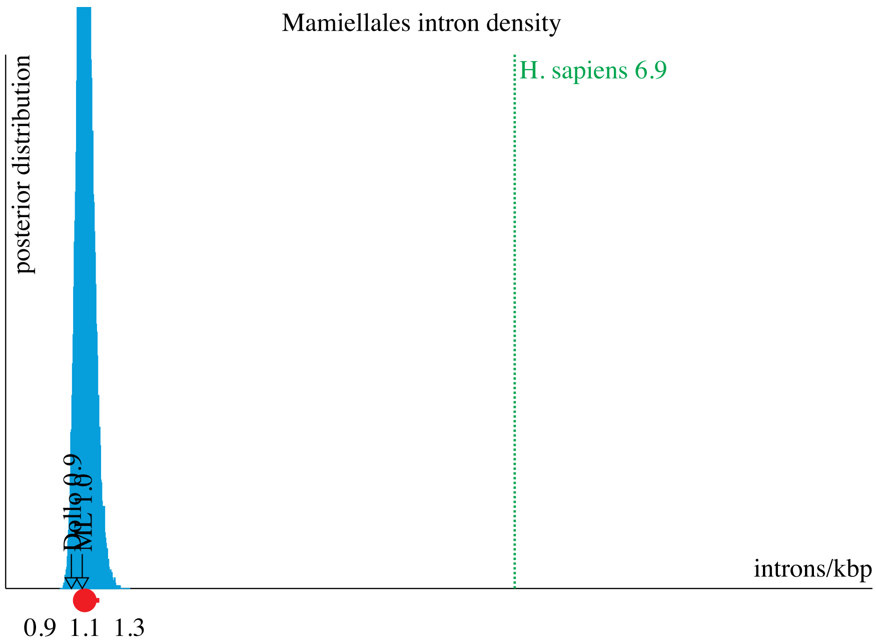

S10.lxxvii Micromonas

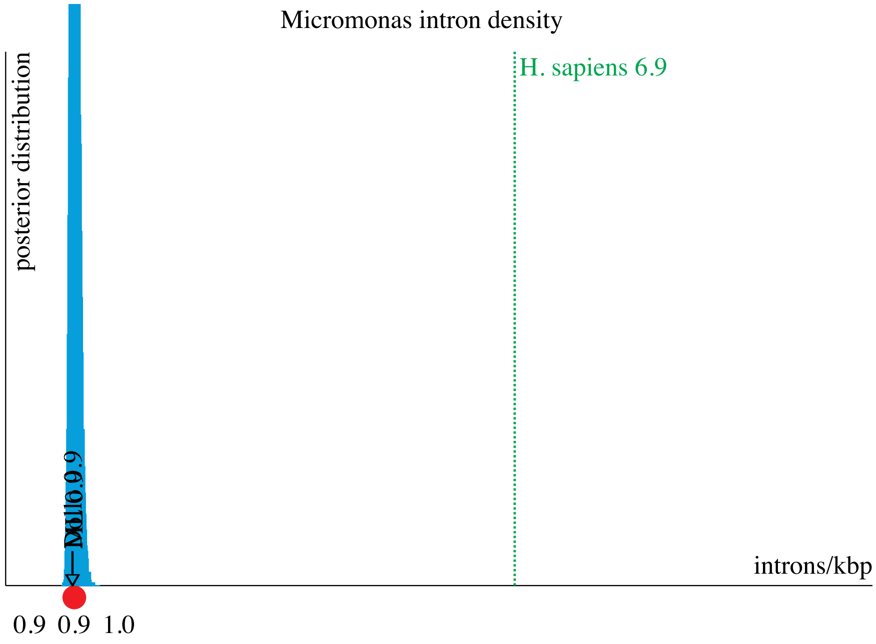

S10.lxxviii Mycosphaerella

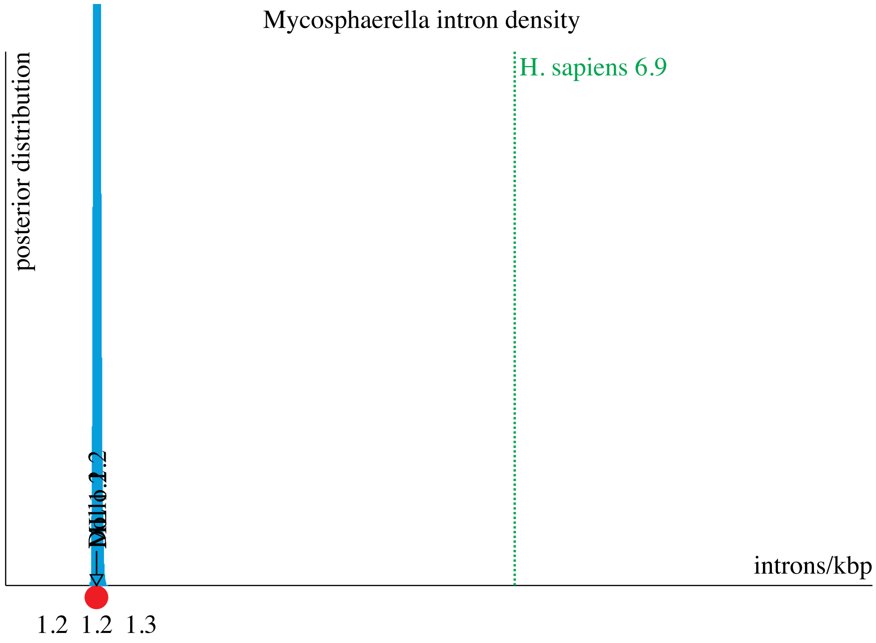

S10.lxxix Onygenales

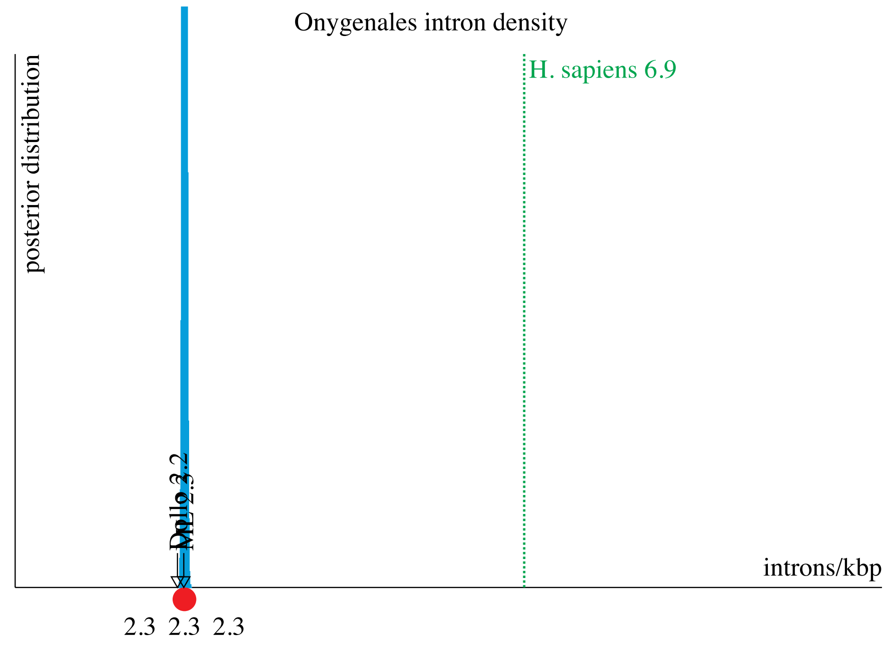

S10.lxxx Ostreococcus

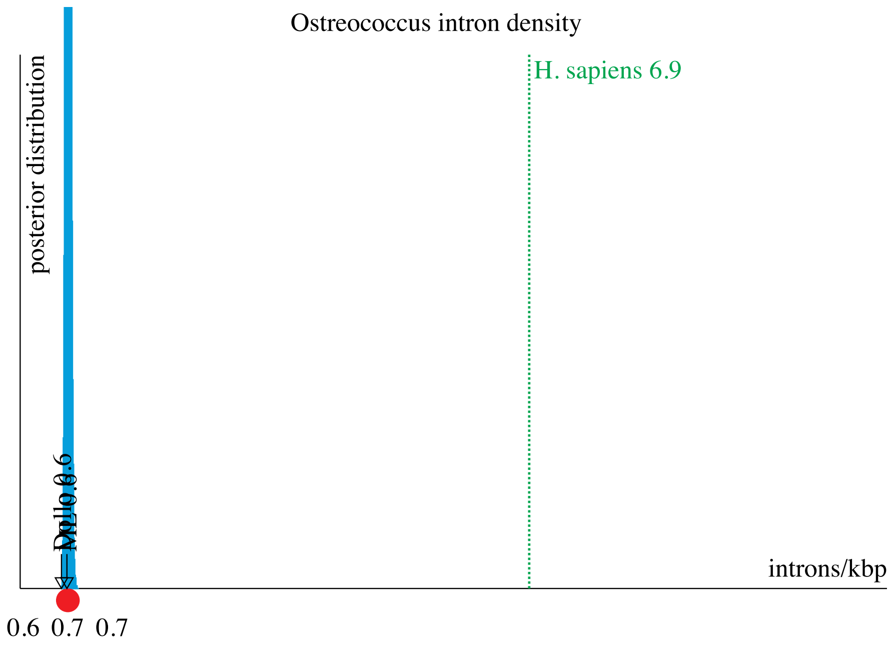

S10.lxxxi Phytophthora

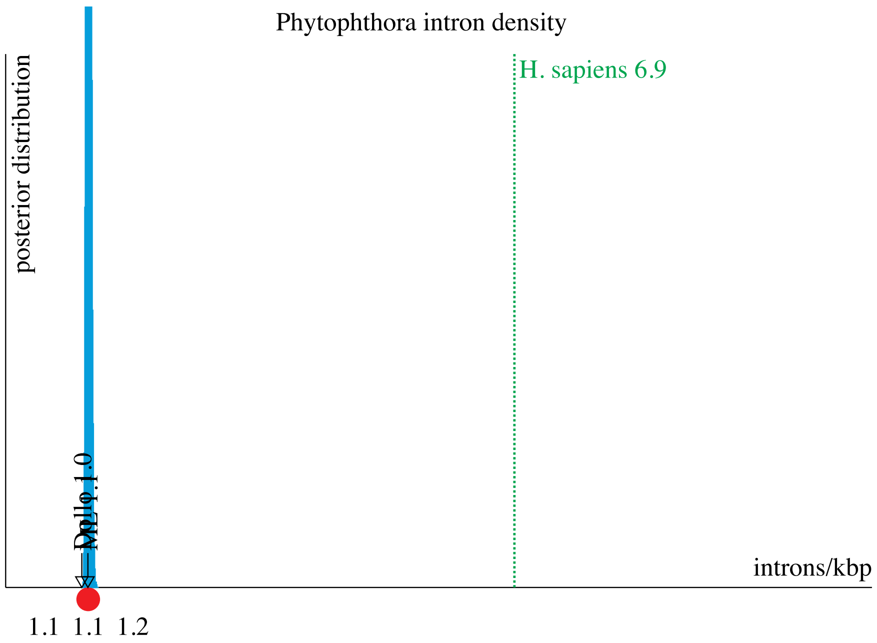

S10.lxxxii Plasmodium

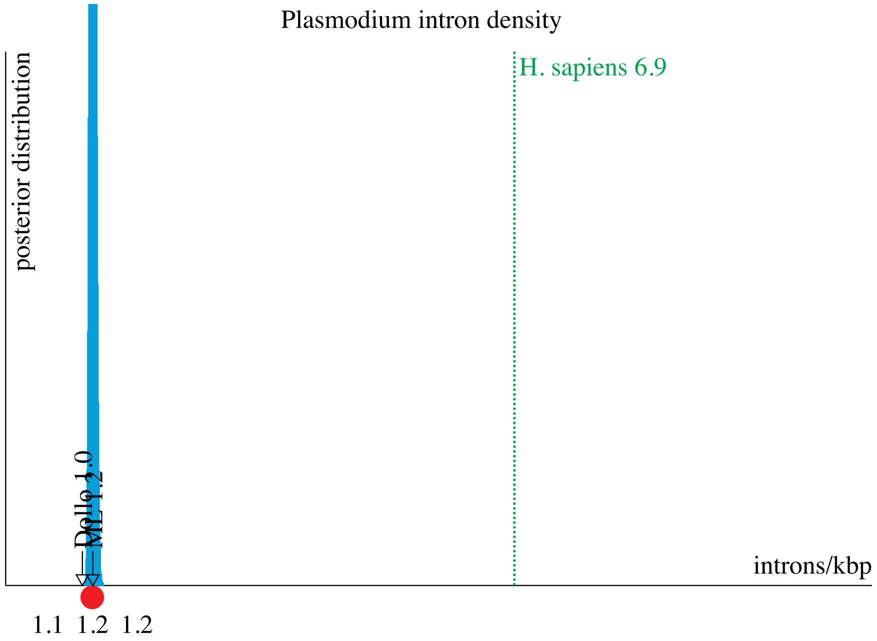

S10.lxxxiii Schizosaccharomycetes

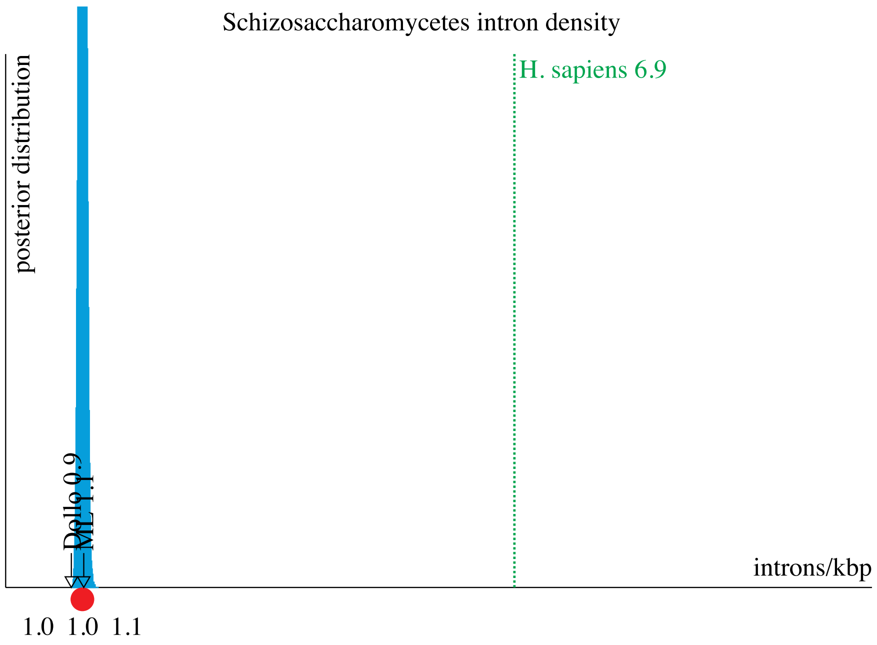

S10.lxxxiv Sordariomycetes

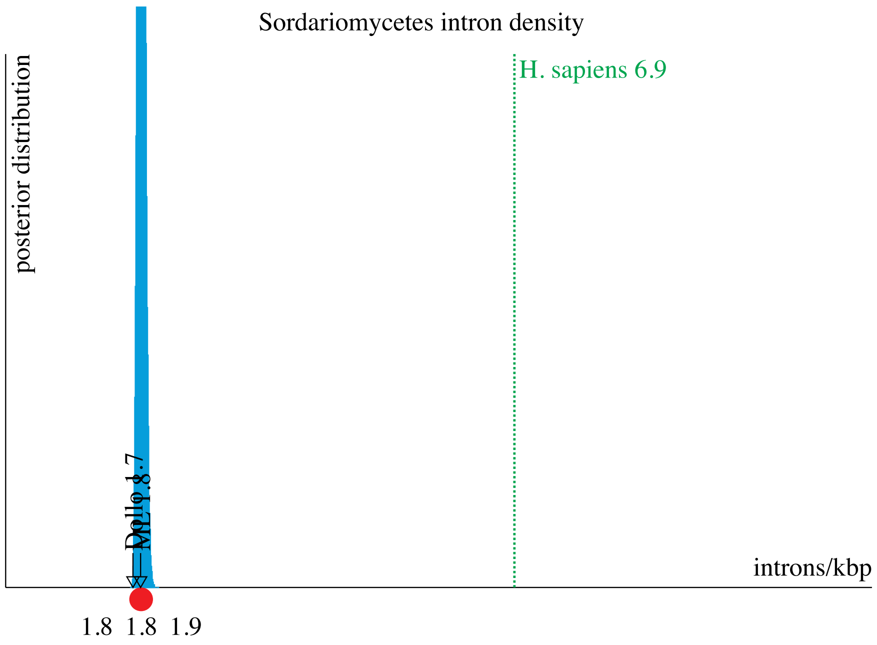

S10.lxxxv Theileria

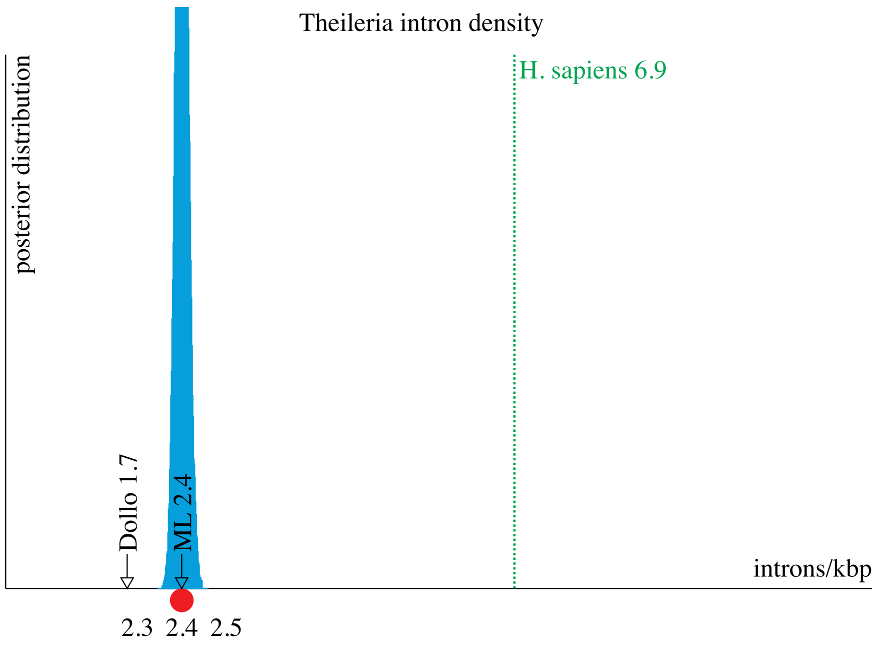

S10.lxxxvi xA3 (P. vivax+P.yoelii)

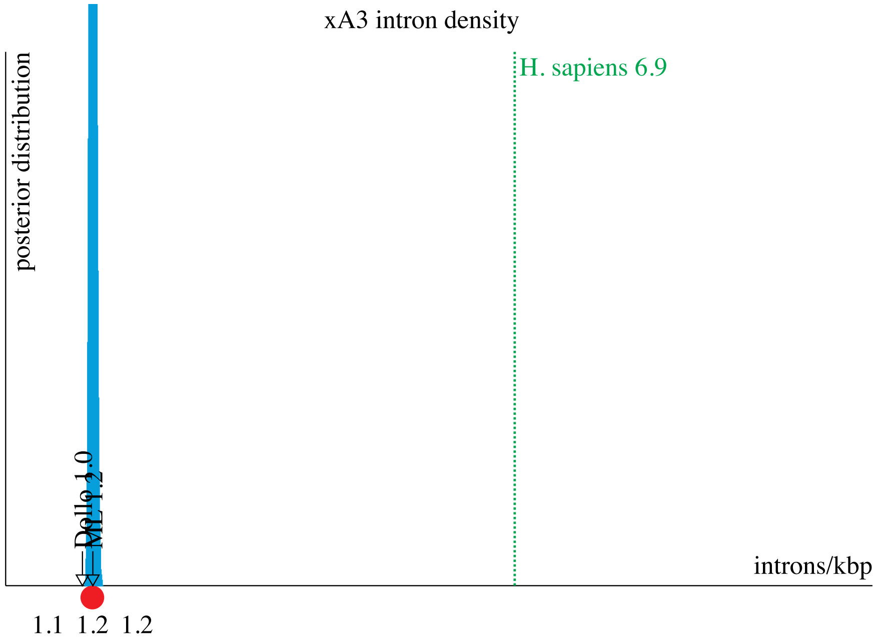

S10.lxxxvii xF3 (Rory+Mcir)

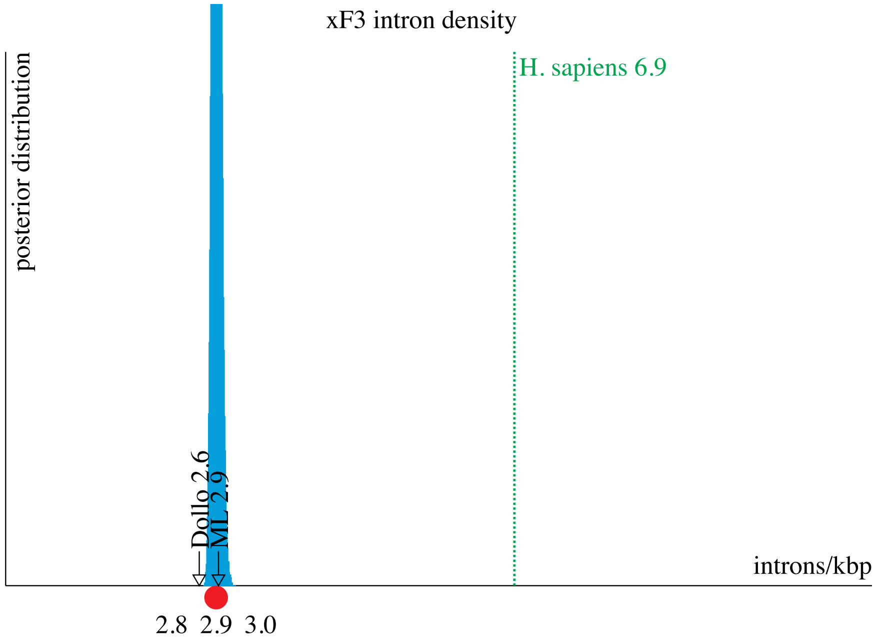

S10.lxxxviii xF5 (Leotiomycetes+Sordariomycetes)

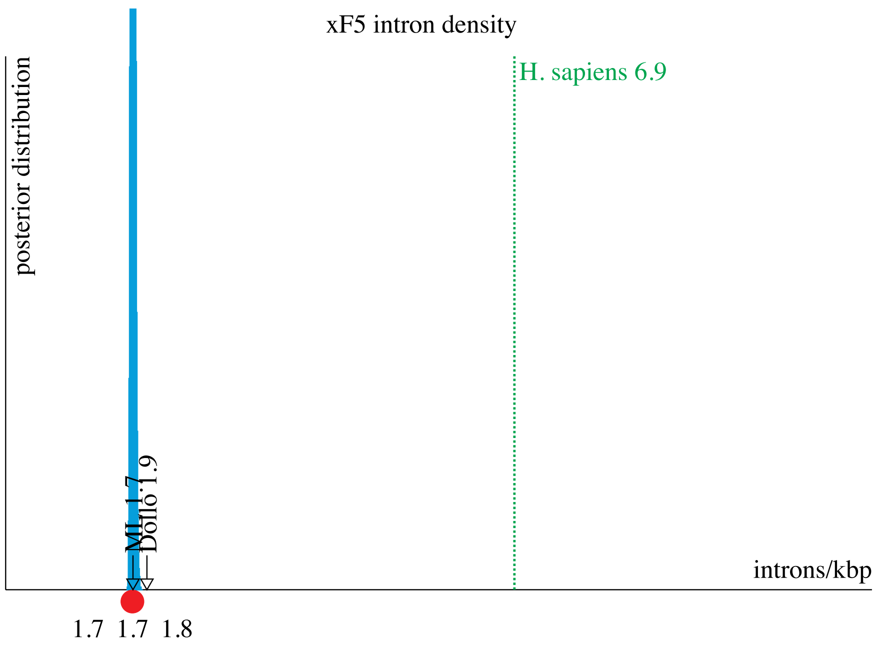

S10.lxxxix xF6 (sister of Leotiomycetes)

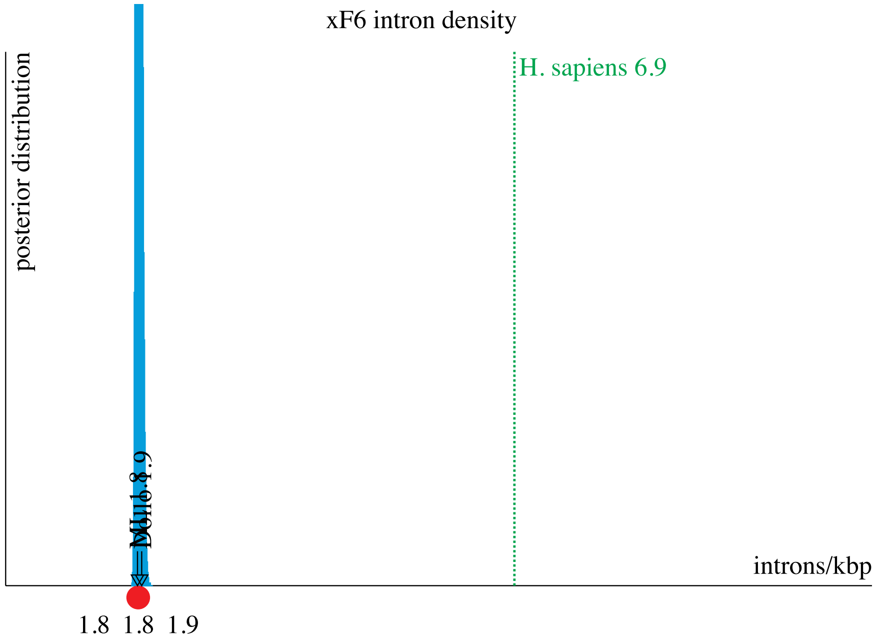

S10.xc xF7 (Eurotiomycetes+Dothideomycetes)

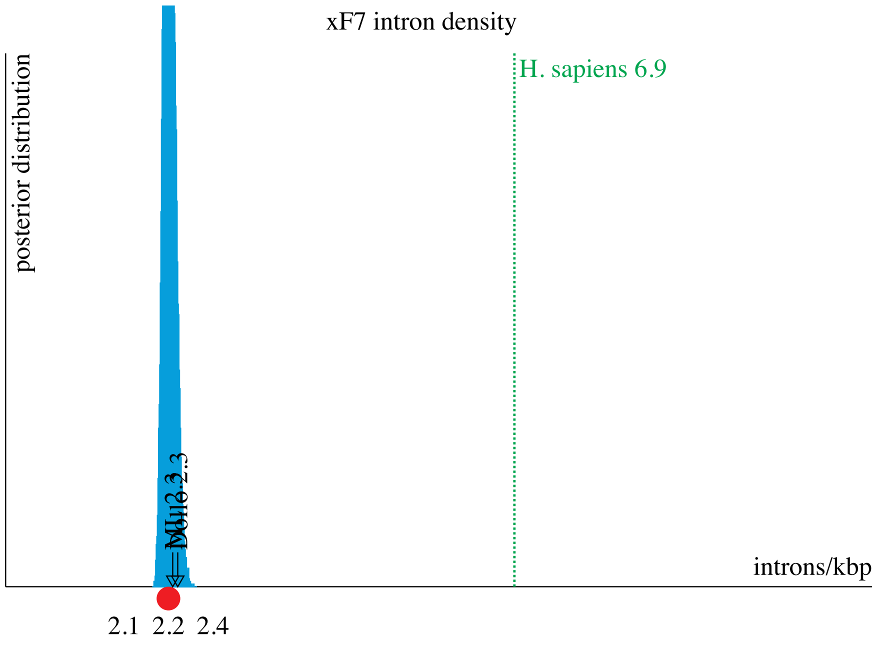

S10.xci xF8 (sister of Mycosphaerella)

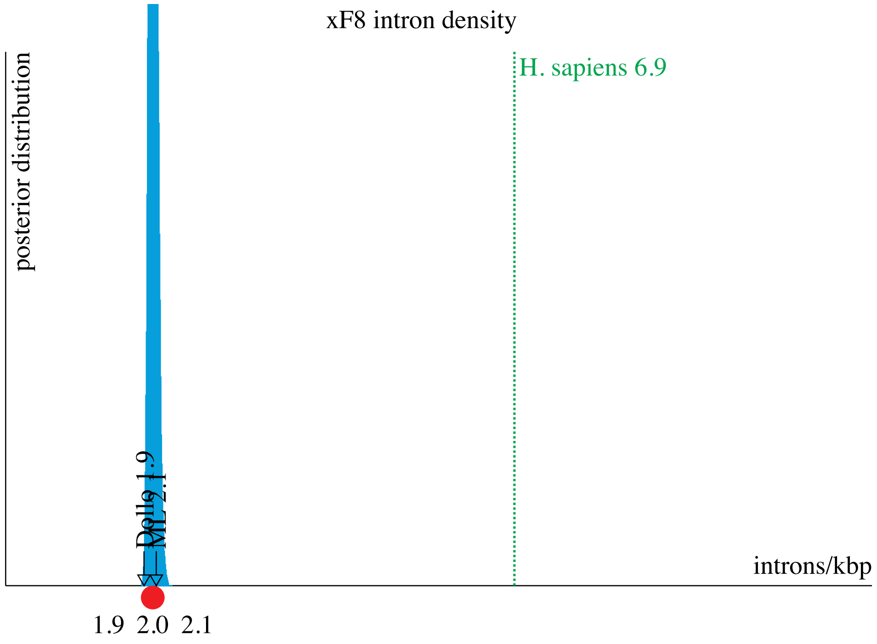

S10.xcii xF11 (sister Cneo)

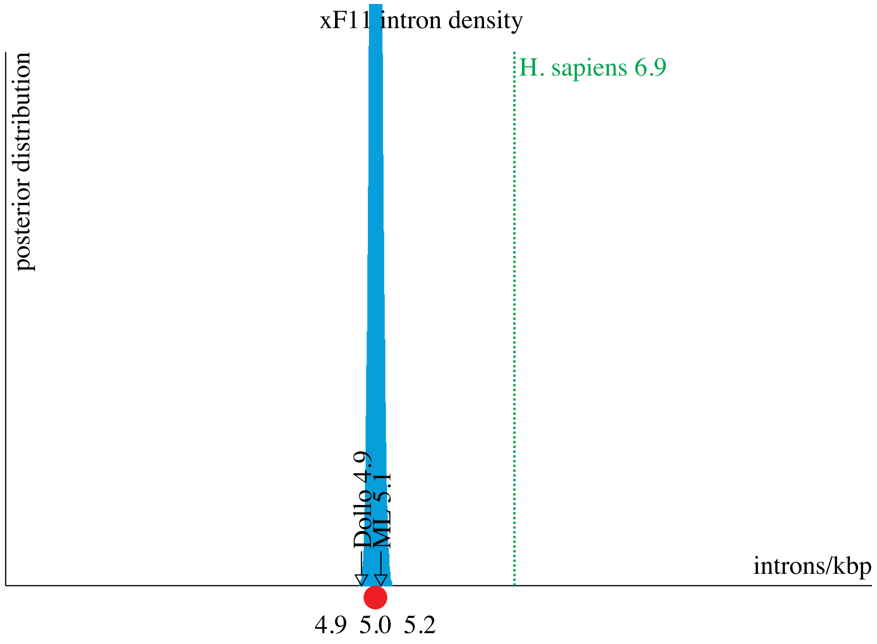

S10.xciii xF9 (Chet+Prep)

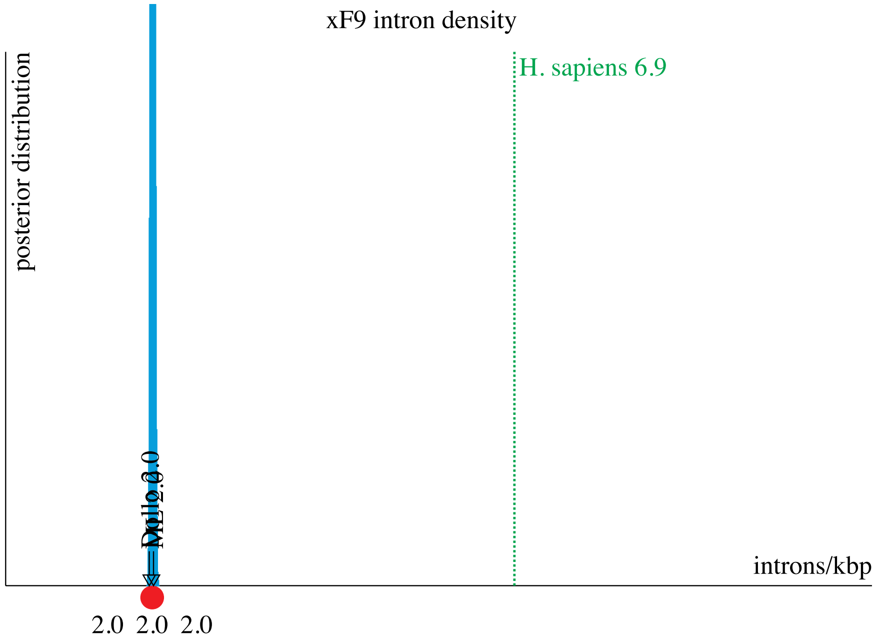

S10.xciv xF12 (Abis+Lbic)

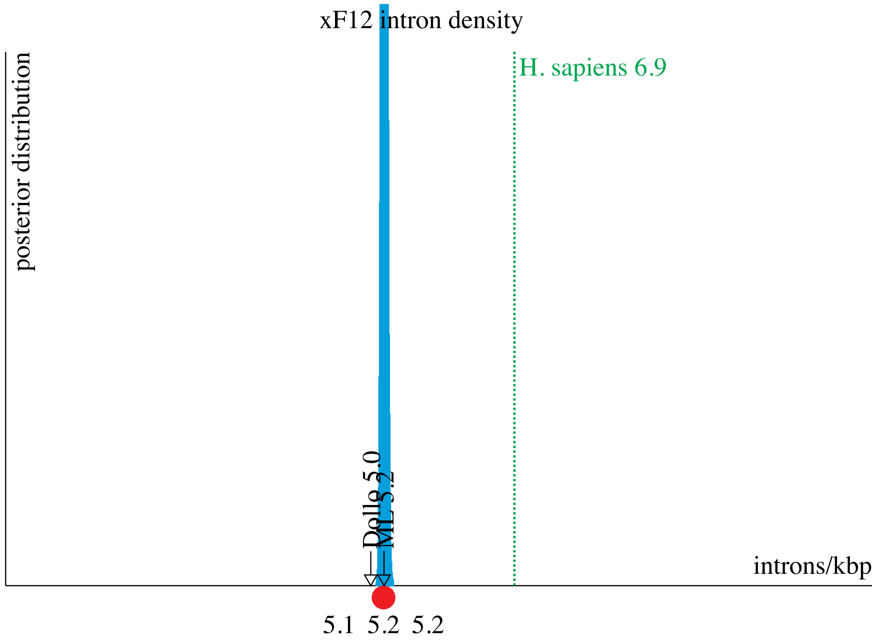

S10.xcv xF13 (Mlar+Pgra)

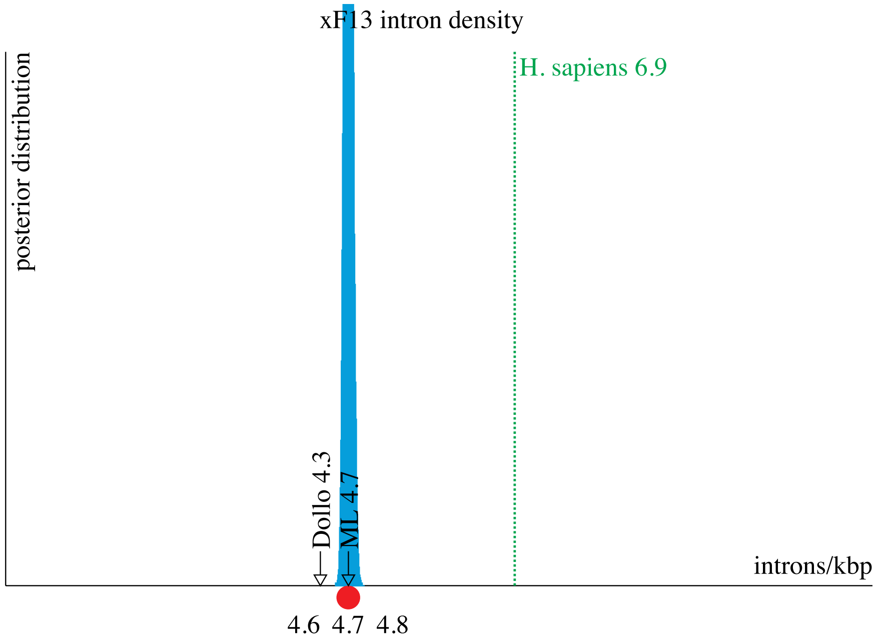

S10.xcvi xH2 (Pram+Pcap)

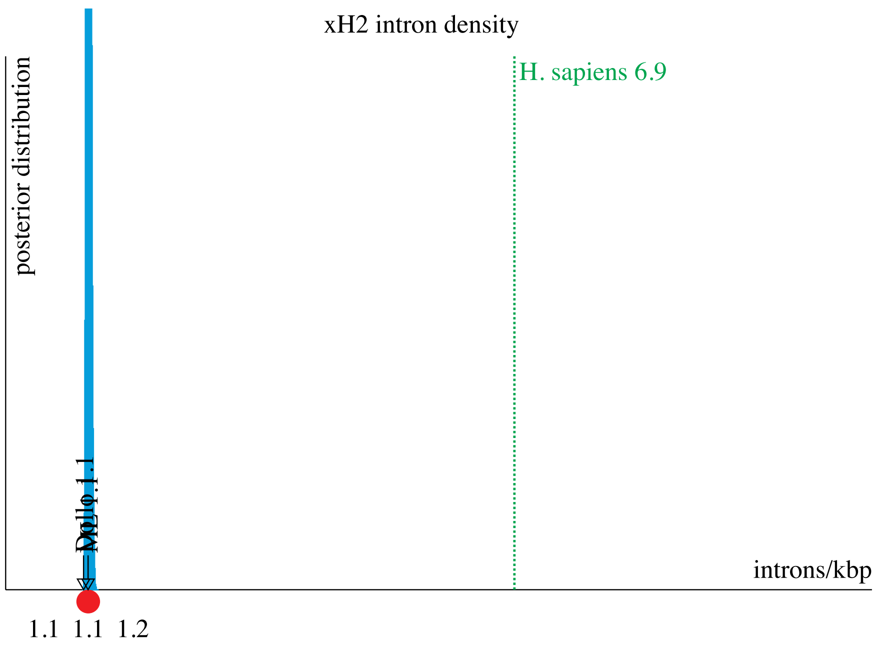

S10.xcvii xO

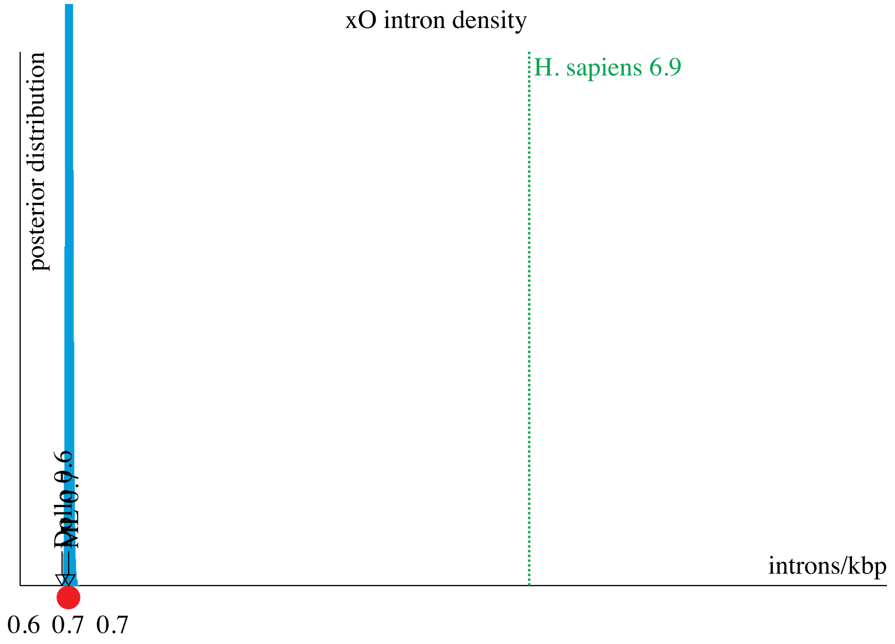

xO intron density
posterior distribution
H. sapiens 6.9
Dollo 0.6
ML 0.7
introns/kbp
0.6
0.7
0.7
