# Supplementary material for: A Detailed History of Intron-rich Eukaryotic Ancestors Inferred from a Global Survey of 100 Complete Genomes
Source: PLoS Comput Biol. 2011 Sep 15;7(9):e1002150. doi: 10.1371/journal.pcbi.1002150 (PMC3174169; doi:10.1371/journal.pcbi.1002150)
Supplement: Text S1 — Detailed methods and results with the illustrating figures and tables. (PDF) [file pcbi.1002150.s002.pdf]

A DETAILED HISTORY OF INTRON-RICH EUKARYOTIC ANCESTORS INFERRED FROM A GLOBAL SURVEY OF 100 COMPLETE GENOMES

Supporting Information

Miklós Csűrös   Igor B. Rogozin   Eugene V. Koonin

May 23, 2011

Contents

1 Supporting Methods **3**

1.1 Species and annotated genomes . . . 3

1.2 Homolog identification . . . 4

1.3 Ortholog set selection . . . 5

1.4 Input table of intron presence and absence . . . 5

2 Convergence **6**

2.1 Priors and proposals . . . 6

2.2 Sampling . . . 7

3 Ambiguity at Amoeoboza and Chromalveolates **11**

4 Ancestral phase distributions **16**

5 Model fit **19**

6 Ancestral inference **21**

7 Simulations **24**

8 Nægleria gruberi **26**

1 Supporting Methods

1.1 Species and annotated genomes

We included the following species in the study: *Aureococcus anophagefferens* (Aano), *Aedes aegypti* (Aaeg), *Agaricus bisporus* (Abis), *Anopheles gambiae* (Agam), *Allomyces macrogynus* ATCC 38327 (Amac), *Apis mellifera* (Amel), *Aspergillus nidulans* FGSC A4 (Anid), *Acyrthosiphon pisum* (Apis), *Arabidopsis thaliana* (Atha), *Babesia bovis* (Bbov), *Batrachochytrium dendrobatidis* (Bden), *Branchiostoma floridae* (Bflo), *Botryotinia fuckeliana* B05.10 (Bfuc), *Brugia malayi* (Bmal), *Bombyx mori* (Bmor), *Coccomyxa* sp. C-169 (C169), *Chlorella* sp. NC64a (C64a), *Caenorhabditis briggsae* (Cbri), *Caenorhabditis elegans* (Cele), *Coprinopsis cinerea* okayama7#130 (Ccin), *Cochliobolus heterostrophus* C5 (Chet), *Coccidioides immitis* RS (Cimm), *Ciona intestinalis* (Cint), *Cryptococcus neoformans var. neoformans* (Cneo), *Chlamydomonas reinhardtii* (Crei), *Capitella teleta* (Ctel), *Capsaspora owczarzaki* ATCC 30864 (Cowc), *Dictyostelium discoideum* (Ddis), *Dictyostelium purpureum* (Dpur), *Drosophila melanogaster* (Dmel), *Drosophila mojavenis* (Dmoj), *Daphnia pulex* (Dpul), *Danio rerio* (Drer), *Entamoeba dispar* (Edis), *Entamoeba histolytica* (Ehis), *Emiliania huxleyi* (Ehux), *Fragilariopsis cylindrus* (Fcyl), *Phanerochaete chrysosporium* (Fchr), *Phaeodactylum tricornutum* (Ftri), *Gallus gallus* (Ggal), *Gibberella zeae* PH-1 (Gzea), *Hydra magnipapillata* (Hmag), *Helobdella robusta* (Hrob), *Homo sapiens* (Hsap), *Ixodes scapularis* (Isca), *Laccaria bicolor* (Lbic), *Lottia gigantea* (Lgig), *Micromonas* sp. RCC299 (M299), *Monosiga brevicollis* (Mbre), *Mucor circinelloides* (Mcir), *Mycosphaerella fijiensis* (Mfij), *Mycosphaerella graminicola* (Mgra), *Magnaporthe grisea* 70-15 (Mgri), *Melampsora laricis-populina* (Mlar), *Micromonas pusilla* CCMP1545 (Mpus), *Neurospora crassa* OR74A (Ncra), *Nægleria gruberi* (Ngru), *Nematostella vectensis* (Nvec), *Nasonia vitripennis* (Nvit), *Ostreococcus* sp. RCC809 (O809), *Ostreococcus lucimarinus* (Oluc), *Oryza sativa japonica* (Osat), *Ostreococcus taurii* (Otau), *Phytophthora capsici* (Pcap), *Plasmodium falciparum* (Pfal), *Puccinia graminis* (Pgra), *Pediculus humanus* (Phum), *Phaeosphaeria nodorum* SN15 (Pnod), *Physcomitrella patens subsp. patens* (Ppat), *Phytophthora ramorum* (Pram), *Pyrenophora tritici-repentis* Pt-1C-BFP (Prep), *Proterospongia* sp. ATCC 50818 (Prsp), *Phytophthora sojae* (Psoj), *Paramecium tetraurelia* (Ptet), *Plasmodium vivax* (Pviv), *Plasmodium yoelii yoelii* (Pyoe), *Rhizopus oryzae* (Rory), *Sorghum bicolor* (Sbic), *Saccharomyces cerevisiae* (Scer), *Schizosaccharomyces japonicus* yFS275 (Sjap), *Schistosoma mansoni* (Sman), *Selaginella moellendorffii* (Smoe), *Schizosaccharomyces pombe* (Spom), *Spizellomyces punctatus* DAOM BR117

(Spun), *Strongylocentrotus purpuratus* (Spur), *Sporobolomyces roseus* (Sros), *Sclerotinia sclerotiorum* 1980 UF-70 (Sscl), *Trichoplax adhaerens* (Tadh), *Theileria annulata* (Tann), *Tribolium castaneum* (Tcas), *Toxoplasma gondii* (Tgon), *Taenopygia guttata* (Tgut), *Theileria parvum* (Tpar), *Thalassiosira pseudonana* (Tpse), *Tetrahymena thermophila* (Tthe), *Ustilago maydis* 521 (Umay), *Uncinocarpus reesii* 1704 (Uree), *Volvox carteri* (Vcar), *Vitis vinifera* (Vvin).

The following annotated genomes were downloaded from the Refseq database (version 40) of the National Center for Biotechnology Information [`ftp://ftp.ncbi.nih.gov/refseq/release/`]: Aaeg, Agam, Amel, Anid, Apis, Atha, Bbov, Bflo, Bfuc, Bmal, Cbri, Ccin, Cele, Cimm, Cint, Cneo, Crei, Ddis, Dmel, Dmoj, Drer, Edis, Ehis, Ggal, Gzea, Hmag, Hsap, Isca, M299, Mgri, Ncra, Nvec, Nvit, Oluc, Osat, Pfal, Phum, Pnod, Ppat, Prep, Ptet, Pviv, Pyoe, Sbic, Scer, Sjap, Sman, Spom, Spur, Sscl, Tadh, Tann, Tcas, Tgon, Tgut, Tpar, Tthe, Umay, Uree, Vvin.

The following genome sequence data were produced by the US Department of Energy Joint Genome Institute `http://www.jgi.doe.gov/` in collaboration with the user community: Aano, Abis, C169, C64a, Chet, Ctel, Dpul, Dpur, Ehux, Fchr, Fcyl, Ftri, Hrob, Lbic, Lgig, Mbre, Mcir, Mfij, Mgra, Mlar, Mpus, Ngru, O809, Otau, Pbla, Pcap, Pgra, Pram, Psoj, Smoe, Sros, Tpse, Vcar.

The following annotated genomes were downloaded from the MIT Broad Institute [`http://www.broadinstitute.org/`]: Amac, Bden, Prsp, Rory, Spun. The annotated Bbom genome was downloaded from the SilkDB (Institute of Sericulture and Systems Biology, Southwest University, China) [`http://silkworm.genomics.org.cn/`].

1.2 Homolog identification

Orthologs were identified by slightly modifying our previously described procedure (Csűrös *et al.*, 2008) We used the eukaryotic ortholog groups (so-called euKOGs and euNOGs) from the eggNog database (Muller *et al.*, 2010). The ortholog groups were not used directly, but only for "seeding" a homolog identification step based on bidirectional best hits between each genomes protein sequences, and the seed groups. Specifically, we used command-line tools of the NCBI software development kit (version 6.1, obtained from `ftp://ftp.ncbi.nlm.nih.gov/toolbox/ncbi_tools/ncbi.tar.gz`) to build BLAST databases on individual genomes and the seed groups. First, the seed groups were used to query each genome. Hits between a seed sequence and the query were retained if (1) had an E-value at most 10^{-9}, (2) had an amino acid identity at

least 50%, and (3) had a score within 90% of the best BLAST hit with the same query. Subsequently, the retained sequences were used to query the seed groups to filter out ambiguous group assignments. Sequences passed the filtering step if they had the highest scoring hit to the same seed group used in the initial search, and hit other groups with at most 98% of the best score.

1.3 Ortholog set selection

In a second phase, the collected paralogs were submitted to our ortholog "weaving" protocol described before (Csűrös *et al.*, 2008) which, by reconciling the gene phylogeny with the given species phylogeny, selected at most one sequence for each species that are highly likely to be orthologs. Finally, the resulting candidate ortholog sets were further filtered by verifying their phylogenetic relationships. In particular, we required a non-negative log-likelihood ratio between the neighbor-joining tree and the known species phylogeny, computed by using PhyML (Guindon and Gascuel, 2003).

1.4 Input table of intron presence and absence

Following a well-established analysis pipeline (Rogozin *et al.*, 2003, 2005; Csűrös *et al.*, 2008) we selected homologous intron-bearing positions by projecting the exon-intron structure onto aligned orthologous coding sequences. The resulting intron dataset is a table of intron absence and presence, in which each column corresponds to a splice site projected onto an unambiguous alignment column (retaining splice phase information), and each row corresponds to one of the 99 terminal taxa. Table entries may be 1 (splice site is present), 0 (no splice site), or "ambiguous," denoting a missing ortholog or an uncertain alignment. The table contains all columns with at most 24 ambiguous entries, and was produced using the Malin software package (Csűrös, 2008) with its default settings for ambiguity. The alignments were done using Muscle (Edgar, 2004) with its default settings.

2 Convergence

2.1 Priors and proposals

Recall that the model is defined by the parameters

$$\boldsymbol{\theta} = \bigl(\alpha_{\text{gain}}, \alpha_{\text{loss}}, \pi, \bigl(t_{uv}, \varrho_{uv}\bigr) \colon uv \in T\bigr).$$

The prior distribution was uniform for every parameter: over the range $[0, 10]$ for $\alpha_{.}$ and t_{uv}, and over the range $[0, 1]$ for π and ϱ_{uv}.

In a typical MCMC proposal, a subset of model parameters was chosen, and then multiplied by a random value. Random multipliers were generated by an exponential distribution restricted to some range $[e^{-\sigma}, e^{\sigma}]$. More precisely, the pseudorandom multipliers were drawn as $\mathsf{M}_\sigma = \exp\bigl(\mathsf{U}_{-\sigma,\sigma}\bigr)$, using a pseudorandom uniform $\mathsf{U}_{-\sigma,\sigma}$ over the interval $(-\sigma, \sigma)$. In an Edge move, for instance, the proposed parameter vector $\boldsymbol{\theta}'$ was produced by setting the length of a randomly picked to $t'_{uv} = t_{uv} \cdot M_\sigma$, and keeping all the other parameter values unchanged. The following table shows the proposals used during MCMC sampling.

**Move**	**Parameter**	**Max. multiplier** (e^σ)	**Frequency**
Root	π	1.44	$2/m$
Var	$\alpha_{\text{gain}}, \alpha_{\text{loss}}$	1.2	$1/m$
Edge	t_{uv}	1.44	$20/m$
Rates	ϱ_{uv}	1.44	$20/m$
Resize	all t_{uv} in a subtree	1.44	$20/m$
StarRates	all ϱ_{uv} incident to a node	1.44	$20/m$

For Edge and Rates moves a random edge was selected uniformly. For a Resize move, a random non-terminal node was selected uniformly, and all edge lengths were multiplied with the same random M_σ. For a StarRates move, a random non-terminal node was selected uniformly, and the ratios on each incident edge (children, and the parent — except for the root) were multiplied with a separately drawn random M_σ.

After the burn-in period, the multiplier range was reduced by setting $\sigma \leftarrow \sigma/2$ for each move type. The Var move was applied only to rate variations with more than 1 discrete categories. The frequency denominator $m = 82{+}v$ depends on the types of permitted Var moves: $m = 82$ for no rate variation across sites, $m = 83$ if loss or gain, but not both vary across sites, and $m = 84$ if both vary across sites.

2.2 Sampling

Sampling data was collected after a burn-in period of 500 thousand steps, subsampling every 1000th subsequent position, across 100 independently launched chains. Figure 1 illustrates the convergence of the log-likelihoods to the same value across all the chains. Figure 2 illustrates the mixing of the sampled reconstructions at LECA. In Figure 3, a mixing efficiency is calculated, determined by across- and within-chain variations. Sampled reconstructions at a given step are independent across chains, and therefore can be used to estimate the standard deviation for the estimated posterior distrbution. We define the mixing coeffecient as the ratio between the estimated standard deviation within a chain, and the standard deviaion of the estimated quantity. With sufficiently sparse subsampling, the coefficient approaches 1. Figure 3 illustrates the superior mixing at most ancestral nodes.

Figure 1 MCMC sampling traces: log-likelihood. The Y axis shows the log-likelihood; 12 of the 100 chains are grouped by fours on the plots. The vertical line on each plot shows the end of the burn-in period, and a horizontal dashed line shows the harmonic average of the likelihoods across all chains after burn-in. ►

Figure 2 MCMC sampling traces after burnin: LECA. The Y axis shows the number of introns at LECA at different sampled reconstrutions across 12 of the 100 chains, grouped by fours. The horizontal dashed line shows the average across all chains after burn-in. ►►

Figure 3 Mixing at ancestral estimates. The standard deviation (SD) was estimated within chains, and across chains. For within-chain variation, the SD was calculated from the 500 post-burnin sampled ancestral intron counts, and then averaged across the chains. For across-chain variation, the SD was calculated from the 100 chains at each MCMC step, and then averaged across the steps. ►►►

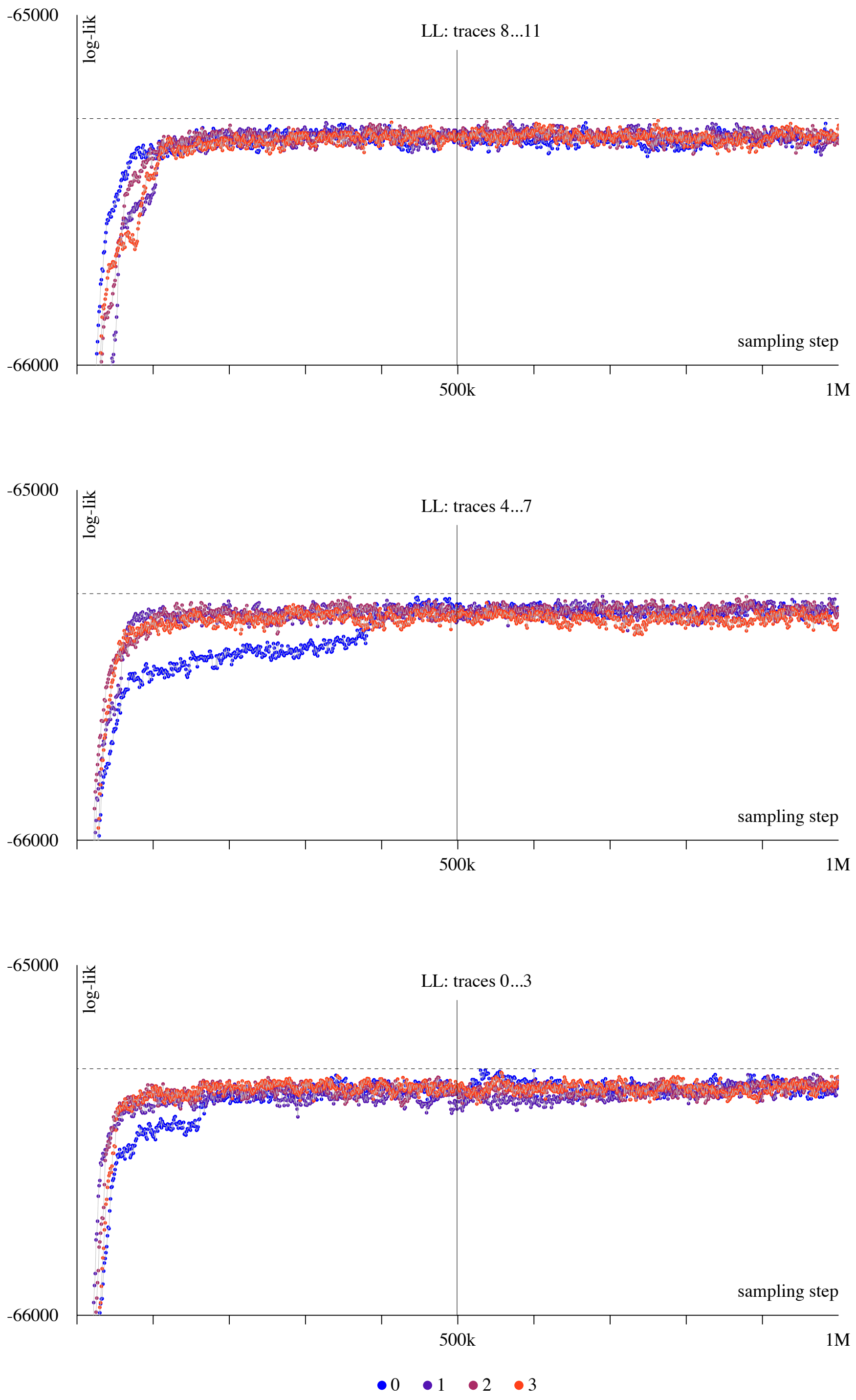
-65000
log-lik
LL: traces 8...11
sampling step
-66000
500k
1M
-65000
log-lik
LL: traces 4...7
sampling step
-66000
500k
1M
-65000
log-lik
LL: traces 0...3
sampling step
-66000
500k
1M
0
1
2
3

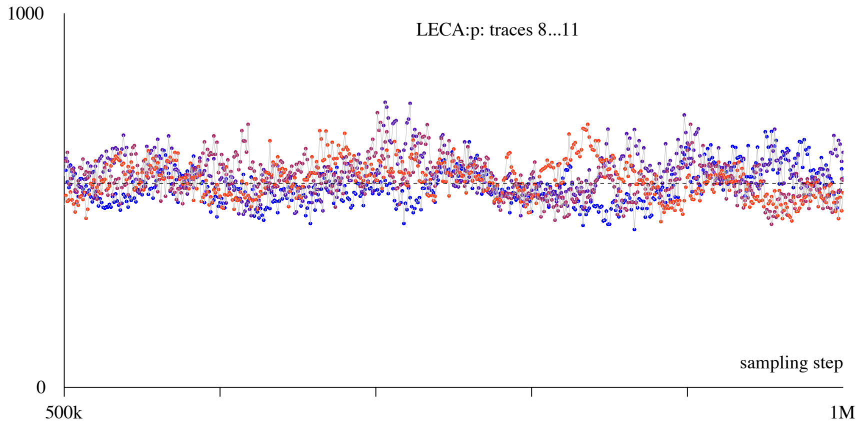
1000
LECA:p: traces 8...11
sampling step
0
500k
1M

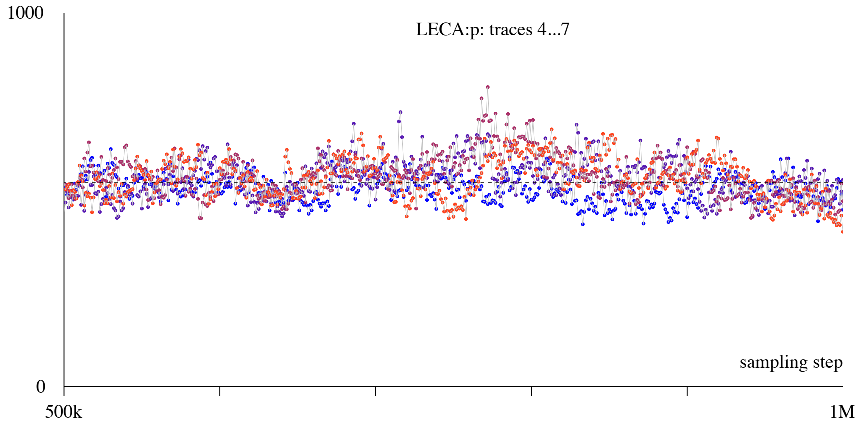
1000
LECA:p: traces 4...7
sampling step
0
500k
1M

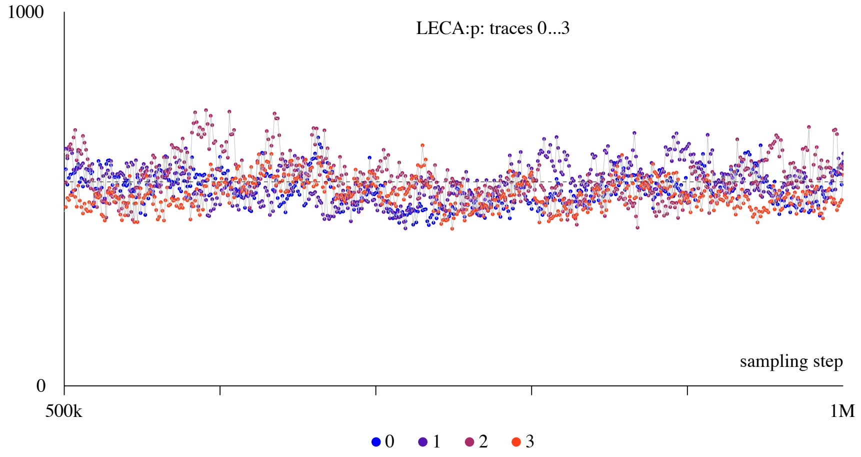
1000
LECA:p: traces 0...3
sampling step
0
500k
1M
0
1
2
3

Mixing efficiency for ancestral reconstruction

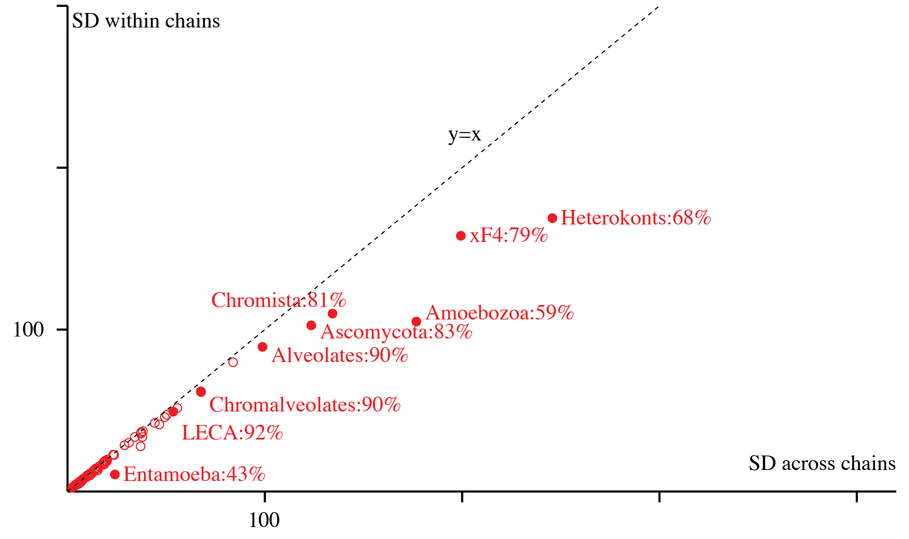

3 Ambiguity at Amoeboza and Chromalveolates

Figure 4 illustrates that the ambiguity at the amobozoan ancestor is clearly caused by the impossibility of inferring the timing of intron losses. Figure floatnbr5 illustrates the more complicated dependencies between chromalveolate ancestors. The tree below facilitates the interpretation by showing the assumed phylogenetic relationships between protists, and the naming of ancestral nodes. Table 6 shows the lack of signal in chromalveolate algae, and the strong intron conservation seen in human introns.

21 protists

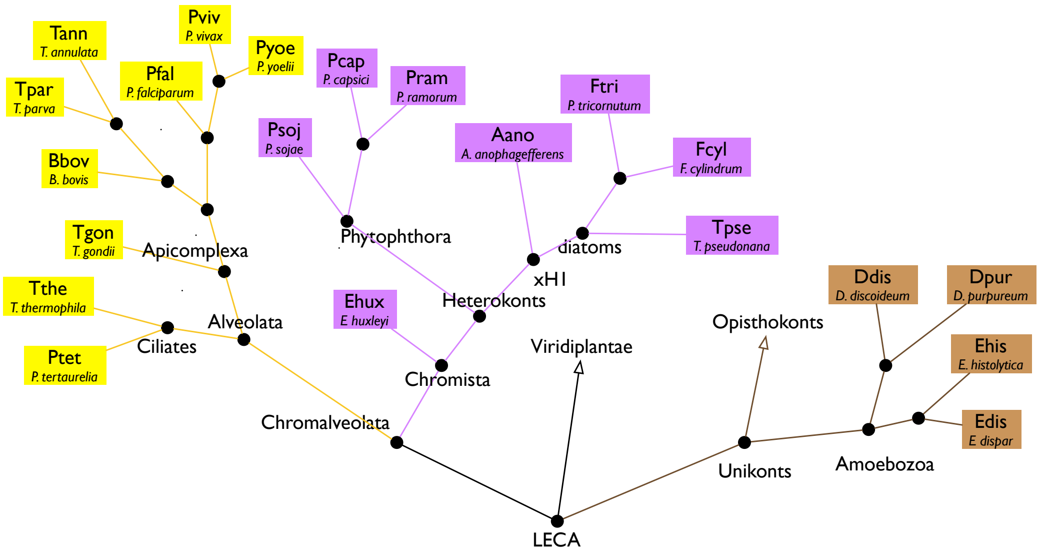

Figure 4 Sampled ancestral reconstructions at the ancestor of Amoebozoa in an MCMC trace. The plots show introns lost on the branches (":l") to the inferred number of introns in the Amoebozoan ancestor ("Amoebozoa:p"). The coloring of the dots indicate when the state was visited. ►

Figure 5 Sampled ancestral reconstructions at the ancestors of chromalveolate algae an MCMC trace. The plots show introns lost on the branches (":l") or inferred to be present (":p") at various positions. The coloring of the dots indicate when the state was visited. ►►

Table 6 Introns in shared positions between some nodes in the data set. The first data row in each subtable gives the reference taxon and the number of sites in which the site has an intron in the reference group's members. The subsequent rows list the intron-sharing patterns between the reference and other taxa t. "Shared" sites have introns in both taxa; "reference-specific" sites have introns in the reference taxon, opposing absent and ambiguous entries in t. "Taxon-specific" sites have introns in t but not in the reference taxon. Notice that the counts include only unambiguosly aligned sites, and, thus, they may not sum up to as expected. For instance, only 654 of 780 (84%) human introns can be unambiguously projected onto homologous amphioxus sequence, and 537 of those amphioxus positions correspond to a splice site. For higher-level taxa, "sharing" was established by at least one terminal descendant taxon: e.g., 568 human introns are projected to an annotated splice site in *at least one* arthropod genome. Percentages in parentheses are calculated with respect to all introns for the taxa in question: either the percentage of introns in the reference ("15% of all human introns do not align with amphioxus introns), or those in t ("20% of amphioxus introns do not align with human introns). ►►►

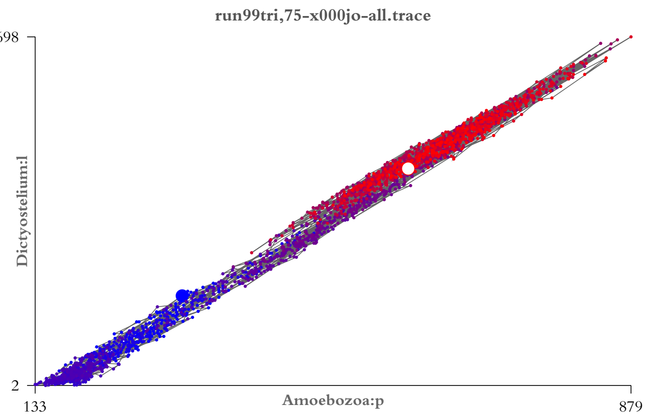
run99tri,75-x000jo-all.trace
698
Dictyostelium:l
2
133
Amoeobozoa:p
879

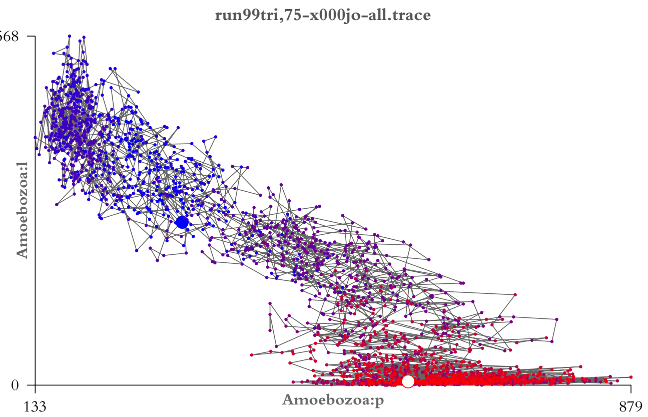
run99tri,75-x000jo-all.trace
568
Amoebozoa:l
0
133
Amoebozoa:p
879

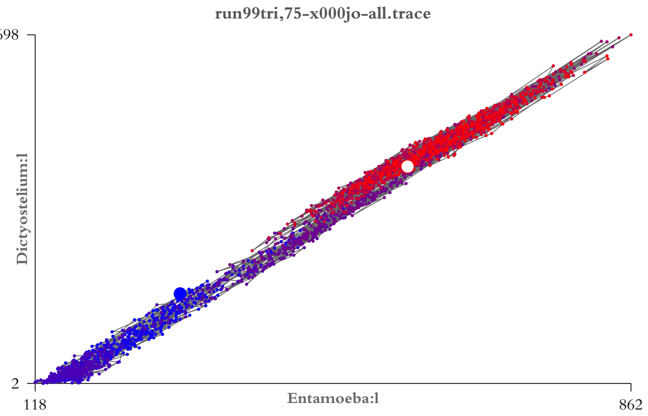
run99tri,75-x000jo-all.trace
698
Dictyostelium:l
2
118
Entamoeba:l
862

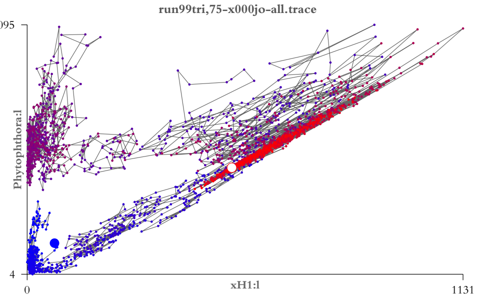
run99tri,75-x000jo-all.trace
1095
Phytophthora:l
4
0
xH1:l
1131

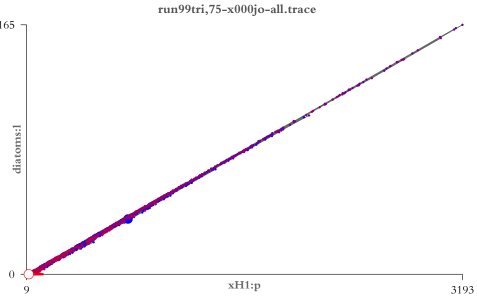
run99tri,75-x000jo-all.trace
3165
diatoms:l
0
9
xH1:p
3193

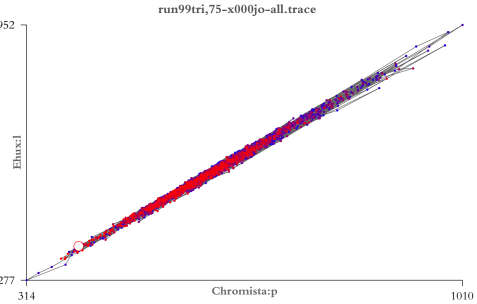
run99tri,75-x000jo-all.trace
952
Ehux:l
277
314
Chromista:p
1010

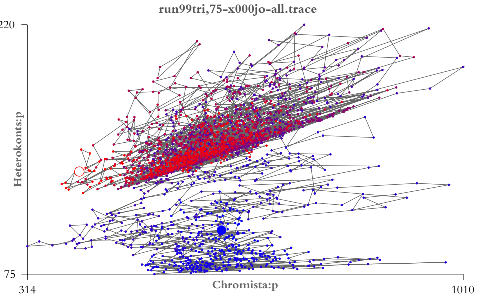
run99tri,75-x000jo-all.trace
1220
Heterokonts:p
75
314
Chromista:p
1010

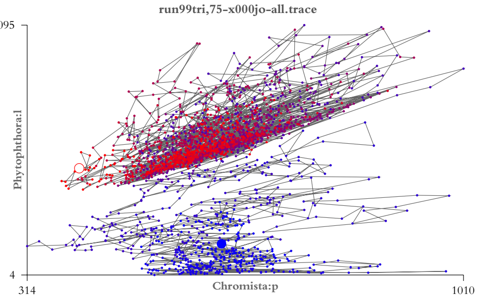
run99tri,75-x000jo-all.trace
1095
Phytophthora:l
4
314
Chromista:p
1010

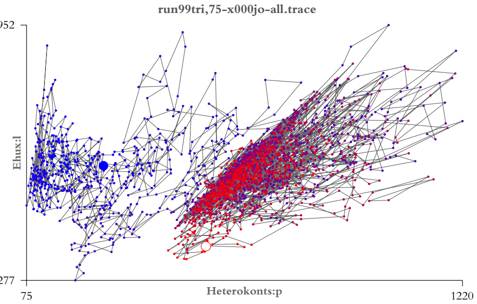
run99tri,75-x000jo-all.trace
952
Ehux:l
277
75
Heterokonts:p
1220

Human introns in shared positions with other taxa

**Taxon**	introns	shared	human-specific	taxon-specific
Hsap [human]	780			
Bflo [amphioxus]	711	537	117 (15%)	140 (20%)
Arthropods	1424	568	200 (26%)	701 (49%)
Tadh [Trichoplax]	893	564	145 (19%)	244 (27%)
Fungi	2565	318	462 (59%)	2036 (79%)
Dictyostelium	212	82	672 (86%)	110 (52%)
Embryophyta [land plants]	853	146	618 (79%)	614 (72%)
Apicomplexa	611	79	622 (80%)	469 (77%)
Phytophthora	165	45	692 (89%)	104 (63%)

Phytophthora introns in shared positions with other taxa

**Taxon**	introns	shared	Phytophthora-spec.	taxon-spec.
Phytophthora	165			
diatoms	208	7	145 (88%)	197 (95%)
Ehux [*E. huxleyi*]	353	3	69 (60%)	302 (86%)
Apicomplexa	611	27	128 (78%)	556 (91%)
Dictyostelium	212	27	132 (80%)	175 (82%)
Chlorophyta [green algae]	1781	42	120 (72%)	1658 (93%)
Embryophyta [land plants]	853	50	111 (67%)	768 (90%)

T. pseudonana introns in shared positions with other taxa

**Taxon**	introns	shared	Tpse-specific	taxon-specific
Tpse [*T. pseudonana*]	115			
Bacillariophycaea [pennate diatoms]	121	28	78 (68%)	81 (67%)
Phytophthora	165	4	109 (95%)	128 (78%)
Ehux [*E. huxleyi*]	353	3	69 (60%)	302 (86%)
Apicomplexa	611	4	105 (91%)	539 (88%)
Dictyostelium	212	4	106 (92%)	181 (85%)
Chlorophyta [green algae]	1781	8	105 (91%)	1505 (85%)
Embryophyta [land plants]	853	5	108 (94%)	726 (85%)

E. huxleyi introns in shared positions with other taxa

**Taxon**	introns	shared	Ehux-specific	taxon-specific
Ehux [*E. huxleyi*]	353			
Phytophthora	165	3	302 (86%)	69 (60%)
Chlorophyta [green algae]	1781	44	297 (84%)	1163 (65%)
Bilateria	2742	47	306 (87%)	1883 (43%)

4 Ancestral phase distributions

Individual splice site histories were reconstructed using the maximum-likelihood model found during sampling. Figure 7 and Table 8 show the reconstructed phases at sites represented in terminal taxa.

**Figure 7**	Intron phase distribution at different nodes. Branch widths are proportional to (inferred) intron density. Pie charts show the relative frequencies of phase-1, -2 and -0 splice sites. Notice that the reconstruction covers only the splice sites in the data set (where phase can be established), and not the extrapolated all-absent profiles.	►
**Table 8**	Intron phase distribution, gains and losses at different nodes. Pie charts show the relative frequencies of phase-1, -2 and -0 splice sites.	►►

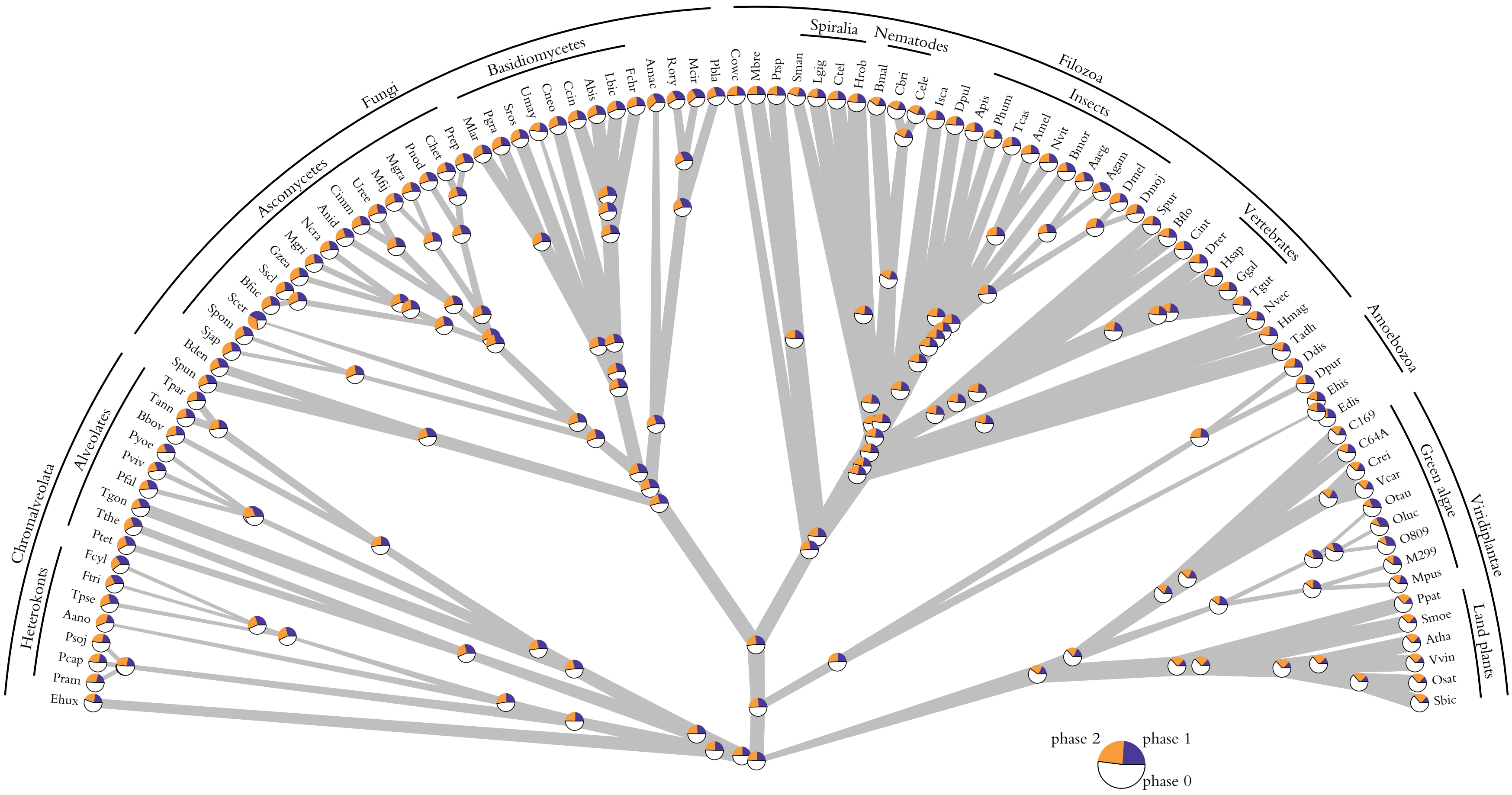

Chromalveolata
Heterokonts
Alveolates
Fungi
Ascomycetes
Basidiomycetes
Spiralia
Nematodes
Filozoa
Insects
Vertebrates
Amoebozoa
Green algae
Viridiplantae
Land plants
Ehux
Pram
Pcap
Psoj
Aano
Tpse
Ftri
Fcyl
Ptet
Tthe
Tgon
Pfal
Pviv
Pyoe
Bbov
Tann
Tpar
Spun
Bden
Sjap
Spom
Scer
Bfuc
Sscl
Gzea
Mgri
Ncra
Anid
Cimm
Uree
Mfij
Mgra
Pnod
Chet
Prep
Mlar
Pgra
Sros
Umay
Cneo
Ccin
Abis
Lbic
Fchr
Amac
Rory
Mcir
Pbla
Cowc
Mbre
Prsp
Sman
Lgig
Ctel
Hrob
Bmal
Cbri
Cele
Isca
Dpul
Apis
Phum
Tcas
Amel
Nvit
Bmor
Aaeg
Agam
Dmel
Dmoj
Spur
Bflo
Cint
Drer
Hsap
Ggal
Tgut
Nvec
Hmag
Tadh
Ddis
Dpur
Ehis
Edis
C169
C64A
Crei
Vcar
Otau
Oluc
O809
M299
Mpus
Ppat
Smoe
Atha
Vvin
Osat
Sbic
phase 2
phase 1
phase 0

Node	Introns	Phase 1	Phase 2	Phase 0	Gain	Phase 1	Phase 2	Phase 0	Loss	Phase 1	Phase 2	Phase 0
Ehux	403	22%	21%	55%	356	21%	21%	57%	484	24%	24%	51%
Pram	148	21%	27%	51%	14	16%	36%	46%	8	21%	19%	59%
Pcap	137	21%	26%	51%	7	29%	1%	69%	12	20%	14%	64%
Psoj	148	21%	26%	52%	8	32%	21%	45%	1	76%	5%	18%
Aano	48	20%	35%	43%	45	20%	36%	42%	123	26%	26%	47%
Tpse	125	27%	28%	44%	85	30%	25%	43%	15	49%	15%	35%
Ftri	64	32%	23%	43%	20	44%	17%	37%	17	36%	29%	33%
Fcyl	98	33%	26%	40%	57	34%	26%	39%	20	22%	29%	47%
Ptet	249	29%	29%	41%	125	25%	29%	45%	211	27%	26%	46%
Tthe	320	30%	27%	41%	46	34%	27%	38%	61	26%	25%	47%
Tgon	548	30%	22%	47%	141	33%	19%	47%	86	26%	23%	50%
Pfal	148	28%	22%	48%	4	9%	30%	60%	5	68%	3%	28%
Pviv	150	30%	22%	47%	0	32%	19%	48%	0	20%	26%	53%
Pyoe	140	30%	19%	49%	18	25%	13%	60%	27	27%	28%	43%
Bbov	174	25%	27%	47%	1	7%	66%	25%	123	24%	27%	47%
Tann	304	26%	26%	47%	6	22%	29%	48%	7	20%	35%	43%
Tpar	304	26%	26%	47%	1	30%	7%	61%	2	9%	51%	39%
Spun	495	29%	25%	44%	28	40%	20%	39%	100	32%	22%	44%
Bden	427	29%	27%	43%	24	30%	35%	34%	163	29%	21%	48%
Sjap	113	26%	30%	42%	3	1%	30%	68%	21	20%	27%	51%
Spom	120	28%	29%	42%	2	20%	47%	31%	13	5%	42%	51%
Scer	15	41%	35%	22%	5	20%	62%	16%	455	27%	27%	45%
Bfuc	243	27%	30%	42%	8	41%	29%	29%	2	29%	4%	65%
Sscl	240	26%	29%	43%	7	38%	7%	53%	3	52%	41%	5%
Gzea	243	26%	31%	41%	42	35%	33%	30%	26	41%	22%	36%
Mgri	230	26%	29%	44%	26	29%	25%	45%	28	30%	31%	38%
Ncra	239	26%	27%	45%	48	26%	23%	49%	42	27%	33%	38%
Anid	272	29%	25%	44%	25	40%	23%	36%	26	26%	36%	36%
Cimm	301	28%	28%	43%	10	50%	18%	31%	0	61%	10%	28%
Uree	288	27%	28%	44%	2	1%	1%	97%	4	56%	7%	36%
Mfij	172	26%	31%	42%	24	21%	43%	34%	8	30%	7%	61%
Mgra	163	27%	27%	45%	11	26%	29%	44%	3	35%	55%	9%
Pnod	246	31%	23%	45%	12	46%	26%	27%	18	19%	40%	40%
Chet	268	29%	25%	45%	18	23%	15%	60%	2	44%	12%	42%
Prep	260	29%	26%	43%	10	31%	47%	20%	2	44%	3%	51%
Mlar	599	28%	30%	41%	20	50%	24%	25%	9	36%	28%	34%
Pgra	595	27%	30%	42%	8	28%	36%	35%	1	72%	12%	14%
Sros	607	27%	30%	42%	210	29%	30%	39%	156	30%	24%	44%
Umay	46	23%	23%	52%	17	30%	26%	42%	558	28%	27%	43%
Cneo	604	28%	29%	42%	252	26%	31%	41%	251	25%	28%	46%
Ccin	595	26%	29%	43%	14	29%	21%	49%	67	34%	23%	42%
Abis	612	27%	29%	42%	3	1%	61%	37%	40	20%	27%	51%
Lbic	643	27%	29%	43%	17	40%	23%	36%	24	39%	24%	35%
Fchr	579	25%	29%	45%	25	4%	16%	78%	81	32%	22%	45%
Amac	204	31%	30%	37%	43	42%	20%	36%	340	30%	22%	47%
Rory	330	32%	25%	41%	4	59%	3%	36%	34	33%	25%	40%
Mcir	304	33%	27%	39%	10	39%	15%	45%	67	27%	20%	52%
Pbla	418	29%	26%	43%	9	40%	20%	39%	48	41%	19%	38%
Cowc	329	24%	26%	48%	52	18%	27%	54%	295	27%	22%	49%
Mbre	666	25%	24%	50%	63	25%	16%	58%	301	23%	19%	56%
Prsp	802	25%	23%	51%	50	21%	17%	61%	153	20%	21%	57%
Sman	533	20%	24%	55%	109	18%	16%	65%	507	25%	22%	52%
Lgig	836	24%	23%	51%	20	49%	24%	25%	115	20%	27%	51%
CspI	762	23%	25%	50%	16	19%	25%	55%	175	21%	18%	59%
Hrob	922	24%	23%	51%	79	40%	21%	37%	79	23%	25%	50%
Bmal	748	19%	20%	60%	84	17%	15%	66%	43	29%	25%	45%
Cbri	404	18%	23%	57%	6	36%	2%	60%	46	19%	21%	59%
Cele	438	18%	23%	57%	14	21%	25%	53%	19	22%	27%	49%
Isca	667	24%	24%	51%	52	29%	23%	46%	326	22%	23%	53%
Dpul	598	23%	25%	51%	112	27%	27%	44%	207	27%	20%	52%
Apis	526	25%	23%	51%	29	39%	25%	34%	218	22%	24%	52%
Phum	459	23%	24%	52%	30	31%	30%	37%	286	25%	22%	51%
Tcas	321	26%	26%	46%	31	41%	15%	43%	375	24%	21%	54%
Amel	459	24%	26%	48%	6	55%	24%	20%	7	21%	23%	54%
Nvit	439	24%	26%	49%	23	36%	22%	41%	44	26%	26%	47%
Bmor	520	26%	24%	49%	115	30%	22%	47%	151	25%	23%	51%
Aaeg	221	27%	23%	49%	5	33%	18%	47%	4	53%	8%	37%
Agam	195	30%	23%	46%	9	31%	11%	57%	34	18%	17%	64%
Dmel	211	22%	31%	45%	2	7%	0%	92%	6	5%	4%	90%
Dmoj	217	22%	30%	46%	3	36%	28%	34%	0	13%	19%	67%
Spur	822	24%	24%	51%	69	33%	29%	37%	219	23%	22%	53%
Bflo	898	23%	23%	53%	46	21%	25%	53%	115	22%	26%	51%
Cint	561	25%	23%	51%	158	27%	26%	46%	538	23%	25%	51%
Drer	838	24%	24%	50%	57	27%	26%	45%	131	18%	27%	54%
Hsap	875	23%	23%	52%	0	4%	43%	51%	25	34%	35%	29%
Ggal	875	24%	25%	50%	93	29%	32%	38%	118	25%	21%	53%
Tgut	883	23%	24%	51%	12	35%	35%	29%	29	23%	18%	57%
Nvec	965	23%	22%	53%	9	36%	15%	47%	87	21%	25%	53%
Hmag	750	24%	24%	51%	79	42%	33%	24%	372	24%	23%	52%
Tadh	988	21%	23%	54%	14	24%	20%	54%	137	30%	21%	47%
Ddis	165	23%	26%	50%	11	25%	25%	48%	27	30%	25%	43%
Dpur	193	26%	24%	49%	29	34%	14%	51%	17	19%	27%	53%
Ehis	18	25%	20%	53%	0	26%	25%	48%	0	21%	31%	47%
Edis	19	25%	20%	54%	0	12%	12%	75%	0	24%	27%	47%
C169	803	18%	19%	62%	147	19%	22%	57%	107	18%	22%	58%
C64A	903	23%	21%	55%	429	27%	25%	46%	300	16%	21%	61%
Crei	789	18%	20%	61%	115	14%	22%	63%	64	10%	20%	69%
Vcar	760	17%	19%	62%	43	14%	21%	63%	21	11%	20%	68%
Otau	71	31%	14%	54%	6	52%	26%	21%	16	31%	9%	58%
Oluc	74	32%	12%	55%	3	39%	2%	58%	12	15%	12%	71%
O809	82	29%	12%	57%	1	88%	3%	8%	1	75%	6%	17%
M299	126	24%	15%	60%	12	30%	14%	54%	3	11%	41%	47%
Mpus	149	21%	17%	60%	50	20%	21%	57%	19	34%	16%	48%
Ppat	699	13%	24%	62%	17	28%	30%	40%	123	16%	23%	59%
Smoe	754	13%	23%	62%	14	25%	21%	52%	57	11%	22%	66%
Atha	752	13%	23%	63%	5	58%	1%	40%	25	1%	29%	68%
Vvin	770	12%	24%	63%	5	18%	44%	37%	7	1%	10%	88%
Osat	770	12%	23%	63%	3	85%	9%	5%	9	7%	25%	66%
Sbic	770	12%	23%	63%	2	2%	47%	50%	9	9%	38%	51%
xH2	142	21%	26%	52%	0	24%	30%	44%	0	40%	19%	40%
Phytophthora	142	21%	26%	52%	49	13%	23%	62%	311	24%	23%	51%
Bacillariophycaea	61	29%	27%	43%	6	35%	17%	47%	0	29%	25%	45%
diatoms	55	29%	28%	42%	27	26%	30%	42%	98	24%	26%	48%
xH1	126	26%	26%	47%	54	25%	27%	47%	332	24%	24%	50%
Heterokonts	403	25%	24%	50%	36	21%	25%	53%	163	24%	23%	52%
Chromista	531	25%	24%	50%	26	22%	24%	53%	21	24%	22%	52%
Ciliates	335	29%	27%	43%	143	32%	27%	40%	378	24%	22%	52%
xA3	150	30%	22%	47%	0	33%	14%	52%	0	25%	25%	49%
Plasmodium	150	30%	22%	47%	8	57%	13%	29%	357	28%	24%	47%
Theileria	305	25%	26%	47%	16	37%	20%	42%	8	32%	28%	38%
xA2	296	25%	26%	47%	10	26%	27%	45%	213	32%	19%	47%
xA1	499	28%	23%	47%	14	28%	22%	49%	8	32%	19%	47%
Apicomplexa	493	28%	23%	47%	295	29%	22%	48%	372	24%	23%	51%
Alveolates	570	25%	24%	50%	58	28%	26%	44%	14	23%	22%	53%
Chromalveolates	526	25%	24%	50%	28	27%	24%	47%	11	22%	23%	53%
Chytridiomycota	566	29%	25%	45%	104	32%	22%	45%	110	28%	22%	49%
Schizosaccharomycetes	131	26%	30%	43%	28	27%	27%	45%	246	28%	25%	45%
Leotiomycetes	237	26%	30%	42%	23	21%	34%	44%	5	39%	25%	34%
Sordariomycetes	232	26%	29%	43%	7	31%	27%	40%	3	34%	42%	22%
xF6	228	26%	29%	43%	17	13%	22%	64%	7	32%	8%	58%
xF5	218	27%	29%	42%	5	25%	30%	44%	70	29%	20%	49%
Onygenales	291	27%	28%	43%	22	29%	40%	30%	4	77%	10%	12%
Eurotiomycetes	272	28%	27%	44%	21	37%	25%	37%	26	35%	27%	37%
Mycosphaerella	156	27%	28%	44%	20	16%	33%	50%	143	27%	27%	45%
xF9	252	29%	25%	44%	12	19%	40%	40%	12	17%	19%	62%
xF8	252	29%	24%	45%	56	35%	24%	40%	83	29%	32%	38%
Dothideomycetes	279	28%	27%	44%	8	30%	29%	39%	6	30%	35%	34%
xF7	277	28%	27%	44%	4	31%	19%	48%	9	24%	33%	42%
Euascomycota	283	28%	27%	44%	68	27%	29%	43%	250	26%	27%	45%
xF4	465	27%	27%	45%	134	26%	27%	45%	18	27%	27%	45%
Ascomycota	349	27%	27%	44%	92	28%	28%	42%	254	29%	25%	44%
xF13	588	27%	30%	42%	214	30%	26%	42%	180	31%	19%	48%
Pucciniomycotina	553	27%	28%	44%	70	23%	35%	40%	56	32%	23%	44%
xF12	650	27%	29%	43%	3	73%	18%	8%	0	11%	26%	62%
Agaricales	647	27%	29%	43%	16	31%	30%	38%	4	5%	27%	67%
xF11	635	27%	29%	43%	96	23%	33%	43%	63	30%	20%	49%
Agaricomycotina	603	28%	27%	44%	17	25%	31%	43%	2	28%	22%	49%
xF10	588	28%	27%	44%	51	23%	32%	43%	3	30%	21%	47%
Basidiomycota	540	28%	26%	44%	111	28%	27%	43%	82	30%	23%	46%
Dikarya	511	28%	26%	45%	30	32%	22%	45%	81	33%	22%	44%
xF3	361	32%	26%	41%	14	46%	19%	33%	109	28%	23%	47%
Zygomycota	457	30%	25%	43%	66	40%	18%	41%	111	31%	22%	45%
xF2	501	29%	26%	44%	3	44%	18%	37%	64	27%	22%	49%
xF1	562	29%	25%	44%	29	44%	21%	33%	40	35%	14%	50%
Fungi	573	28%	25%	45%	31	39%	21%	39%	91	21%	18%	59%
Choanoflagellates	904	24%	23%	51%	420	25%	20%	53%	335	24%	21%	53%
Annelids	922	23%	23%	52%	1	35%	17%	46%	10	26%	29%	43%
Lophotrochozoa	931	23%	24%	52%	1	28%	22%	48%	1	26%	20%	53%
Spiralia	931	23%	24%	52%	6	40%	27%	32%	11	39%	27%	33%
Caenorhabditis	443	18%	23%	57%	152	17%	25%	56%	416	20%	20%	58%
Nematodes	708	19%	21%	58%	239	15%	18%	65%	470	24%	25%	50%
Hemiptera	715	23%	23%	52%	5	32%	22%	45%	2	23%	24%	51%
Hymenoptera	460	24%	26%	49%	38	25%	32%	42%	244	24%	21%	53%
Culicidae	220	28%	22%	48%	11	45%	18%	35%	120	22%	30%	47%
Drosophilidae	214	22%	30%	46%	7	31%	40%	27%	123	31%	17%	51%
Diptera	329	25%	25%	48%	24	37%	26%	35%	251	26%	23%	50%
xM5	556	25%	24%	49%	74	31%	25%	43%	185	23%	23%	52%
xM4	667	24%	24%	51%	2	24%	28%	47%	0	20%	20%	58%
xM3	665	24%	24%	51%	36	31%	19%	49%	84	22%	16%	61%
Insects	713	23%	23%	52%	29	31%	23%	44%	9	22%	24%	52%
xM2	693	23%	23%	53%	116	27%	23%	48%	365	25%	25%	49%
Arthropods	941	23%	24%	52%	6	31%	30%	37%	3	22%	24%	52%
Ecdysozoa	938	23%	24%	52%	5	21%	22%	56%	3	21%	25%	52%
Protostomes	936	23%	24%	52%	14	34%	43%	21%	51	25%	24%	50%
Aves	900	23%	24%	52%	0	41%	26%	31%	0	31%	27%	41%
Amniota	900	23%	24%	52%	1	7%	44%	48%	12	15%	53%	30%
Vertebrates	911	23%	24%	51%	48	26%	33%	39%	78	25%	21%	53%
xM6	941	23%	23%	52%	13	32%	27%	40%	38	22%	22%	55%
Chordates	966	23%	23%	52%	7	7%	40%	52%	13	15%	34%	50%
Deuterostomes	973	23%	23%	52%	0	19%	25%	55%	1	30%	19%	50%
Bilateria	973	23%	23%	52%	30	48%	16%	34%	172	23%	20%	56%
Cnidaria	1043	23%	23%	53%	12	41%	13%	45%	84	19%	26%	53%
xM1	1115	22%	23%	53%	6	23%	21%	54%	1	19%	19%	60%
Metazoa	1110	22%	23%	53%	307	18%	21%	60%	17	23%	21%	55%
Holozoa	820	24%	24%	51%	264	19%	23%	56%	17	29%	22%	47%
Filozoa	572	26%	24%	48%	31	26%	18%	54%	93	31%	22%	46%
Opisthokonts	634	27%	24%	48%	139	31%	24%	44%	15	18%	24%	56%
Dyctiostelium	181	24%	26%	49%	22	25%	24%	49%	202	26%	23%	49%
Entamoeba	18	25%	20%	53%	2	7%	7%	85%	344	25%	25%	49%
Amoebozoa	361	25%	25%	49%	49	24%	24%	50%	198	26%	22%	50%
Unikonts	510	26%	24%	49%	48	27%	25%	46%	48	15%	22%	61%
Volvocales	738	18%	19%	62%	267	20%	21%	57%	303	20%	21%	58%
xG2	774	18%	19%	62%	14	18%	23%	58%	2	18%	22%	59%
xG1	762	18%	19%	62%	377	18%	20%	61%	24	19%	24%	55%
xO	83	29%	12%	57%	2	36%	1%	62%	0	26%	23%	49%
Ostreococcus	82	29%	12%	57%	6	46%	7%	46%	54	17%	18%	63%
Micromonas	117	23%	16%	60%	8	17%	22%	60%	21	24%	16%	59%
Mamiellales	130	23%	15%	60%	49	22%	20%	57%	329	16%	20%	63%
Chlorophyta	410	18%	18%	63%	84	17%	18%	64%	333	16%	25%	57%
eudicots	772	12%	23%	63%	0	48%	5%	46%	7	4%	39%	56%
monocots	776	12%	23%	63%	12	21%	7%	70%	15	20%	24%	55%
Angiosperms	779	12%	23%	63%	2	3%	3%	92%	21	31%	17%	51%
Tracheophyta	798	13%	23%	63%	6	9%	25%	64%	13	23%	24%	52%
Embryophyta	805	13%	23%	62%	268	9%	25%	65%	122	26%	18%	55%
Viridiplantae	659	17%	22%	60%	329	11%	20%	67%	179	28%	23%	47%
LECA	510	25%	23%	50%								

5 Model fit

Model fit was assessed by computing the the harmonic average of the likelihood in a trace, after the burn-in period, denoted as H. In the limit, the statistic H is proportional to the so-called marginal likelihood, and the ratio of H between two models may serve as a proxy for the Bayes factor (Newton and Raftery, 1994). The distribution of the statistic was computed from the 100 MCMC traces, in order to interpret the significance of differences seen between H of different models. In one set of experiments, we selected the rate variation model with a trifurcating LECA. As Figure 9 shows, multiple gain categories do not improve the models dramatically. Multiple loss categories give significant improvements, but the returns are diminishing beyond 4 categories. The most likely root resolution (CU,P) is the bifurcation between plants and the other two supergroups, but the average likelihoods do not differ more than between the trifurcating traces.

Figure 9 Model fit for different models and phylogeny rootings. The X axis plots the natural logarithm of H for different models, computed from 500 subsampled MCMC steps. Rate variation models are denoted by LxGy for x loss and y gain categories. The shaded area shows the empirical cumulative distribution function (cdf) of H in 100 traces with the L4G1 model chosen for the ancestral inference. Alternative model scores are projected onto the cdf for establishing significance levels: for instance, L5G1 has a score H surpassing 64 of 100 L4G1 traces. Different LECA rooting models (tri is the rooting chosen for the inference) are denoted as shown below. ▶

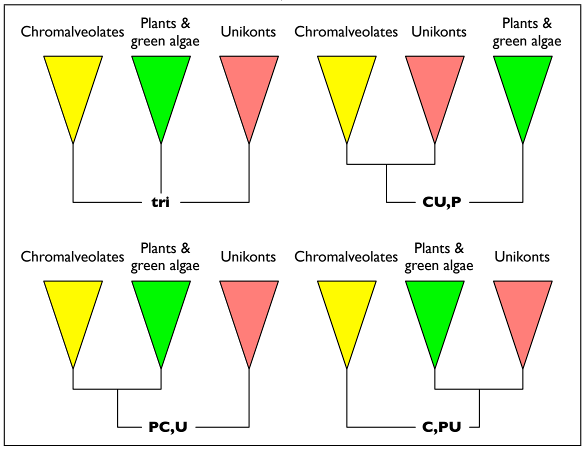

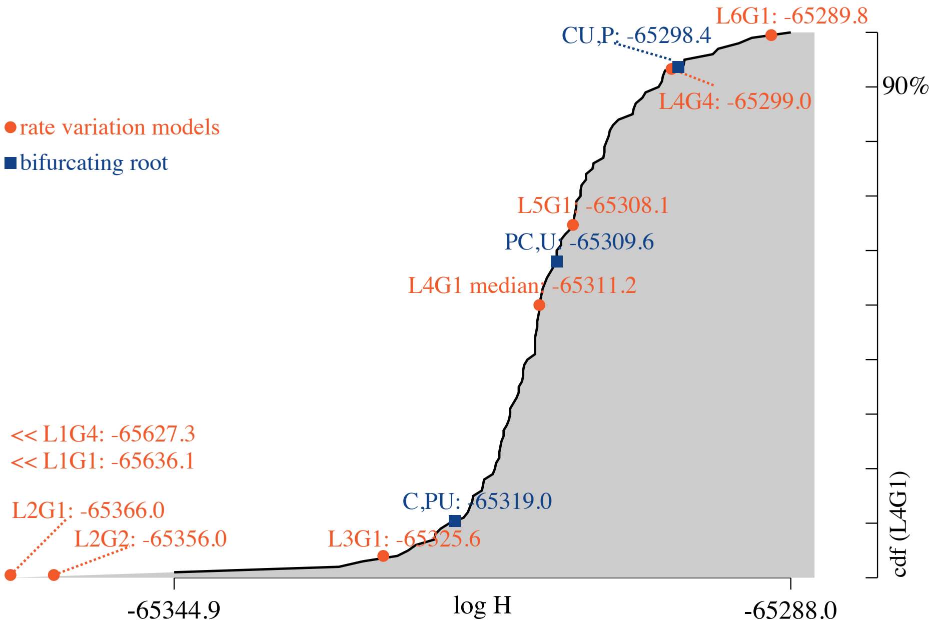

L6G1: -65289.8
CU,P: -65298.4
L4G4: -65299.0
90%
rate variation models
bifurcating root
L5G1: -65308.1
PC,U: -65309.6
L4G1 median: -65311.2
<< L1G4: -65627.3
<< L1G1: -65636.1
C,PU: -65319.0
L2G1: -65366.0
L2G2: -65356.0
L3G1: -65325.6
-65344.9
log H
-65288.0
cdf (L4G1)

6 Ancestral inference

**Figure S10.i–xcvii**	Ancestral density and confidence intervals. The plots show the posterior distribution of the ancestral intron density inferred from the sampling chains. On each plot, the horizontal red line shows the median (by the dot) and the 95% ($\pm 47.5\%$) credible interval around it estimated from 50000 subsampled MCMC steps. The Dollo parsimony and maximum-likelihood (ML) reconstructions are also shown.	Separate PDF file
**Figure 11**	Number of ancestral introns in different reconstructions. The plots compare the number of ancestral introns inferred by MCMC sampling (median in 50000 subsampled steps) with the inference by Dollo parsimony, and by the maximum-likelihood (ML) model found during sampling.	►
**Figure 12**	Lineage-specific loss and gain rates ($\bar{\mu}_{uv}$ and $\bar{\lambda}_{uv}$). On both plots, the X axis shows the median rate seen in the 50000 MCMC-sampled steps. The Y axis plots the rate in the maximum-likelihood model. The two models do not differ much in their parameters.	►►

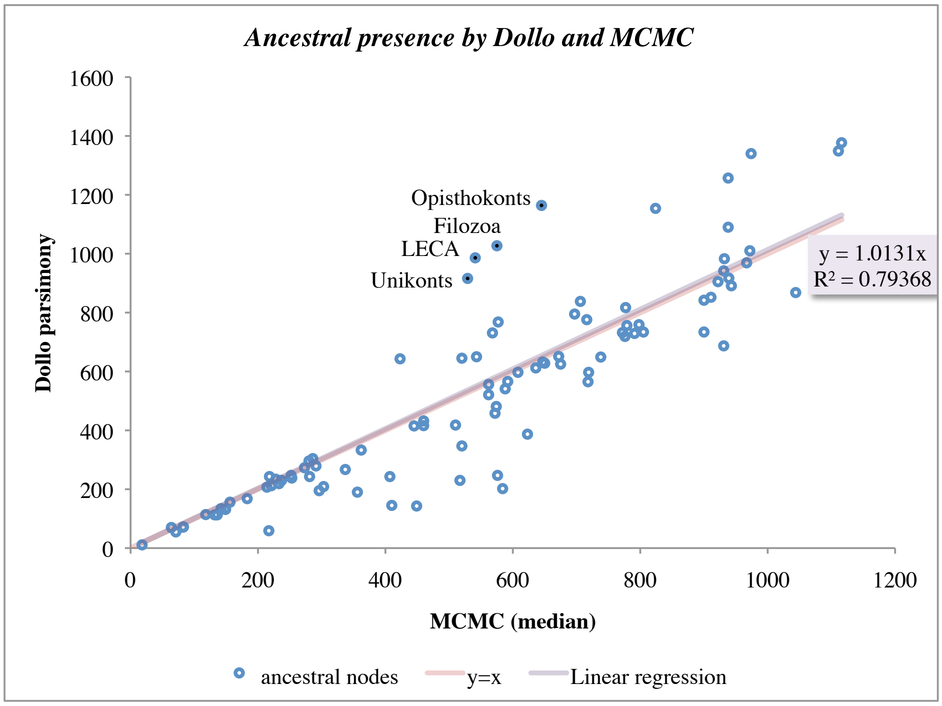
Ancestral presence by Dollo and MCMC
Dollo parsimony
MCMC (median)
Opisthokonts
Filozoa
LECA
Unikonts
y = 1.0131x
R² = 0.79368
0
200
400
600
800
1000
1200
1400
1600
0
200
400
600
800
1000
1200
ancestral nodes
y=x
Linear regression

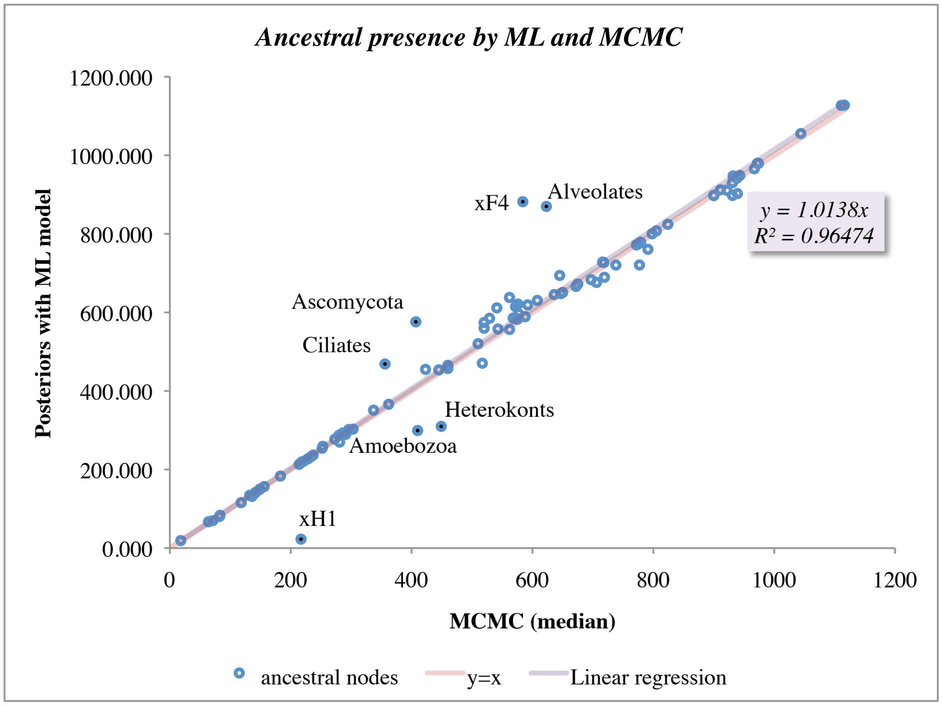
Ancestral presence by ML and MCMC
Posteriors with ML model
MCMC (median)
xF4
Alveolates
Ascomycota
Ciliates
Heterokonts
Amoebozoa
xH1
y = 1.0138x
R² = 0.96474
0.000
200.000
400.000
600.000
800.000
1000.000
1200.000
0
200
400
600
800
1000
1200
ancestral nodes
y=x
Linear regression

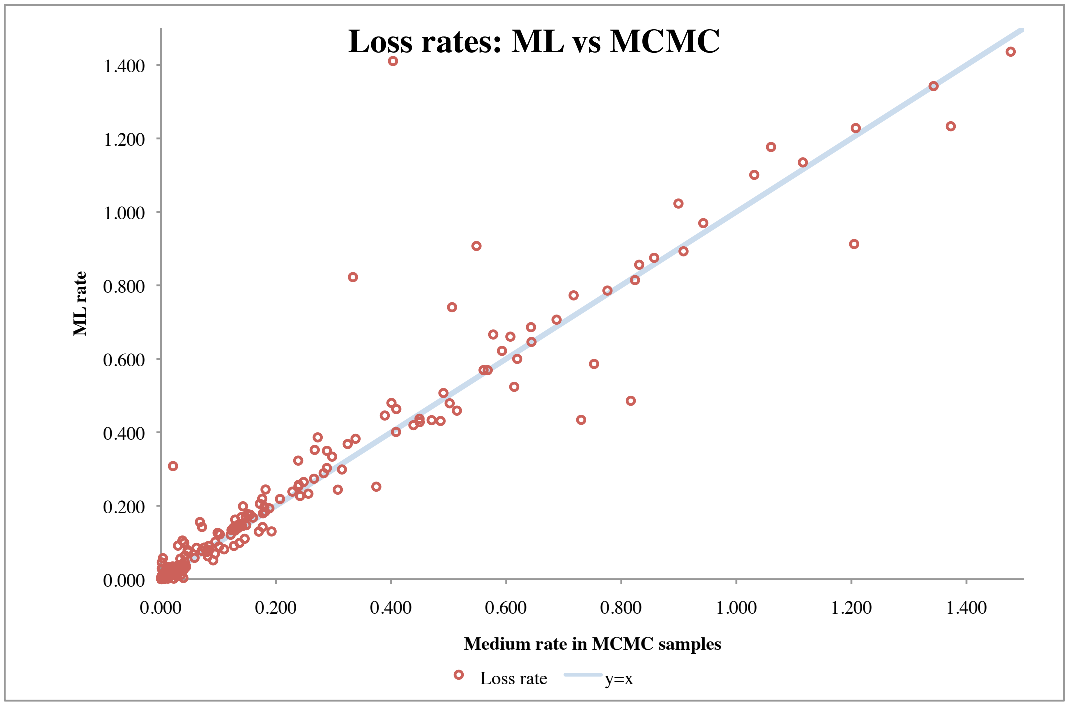
Loss rates: ML vs MCMC
ML rate
Medium rate in MCMC samples
0.000
0.200
0.400
0.600
0.800
1.000
1.200
1.400
Loss rate
y=x

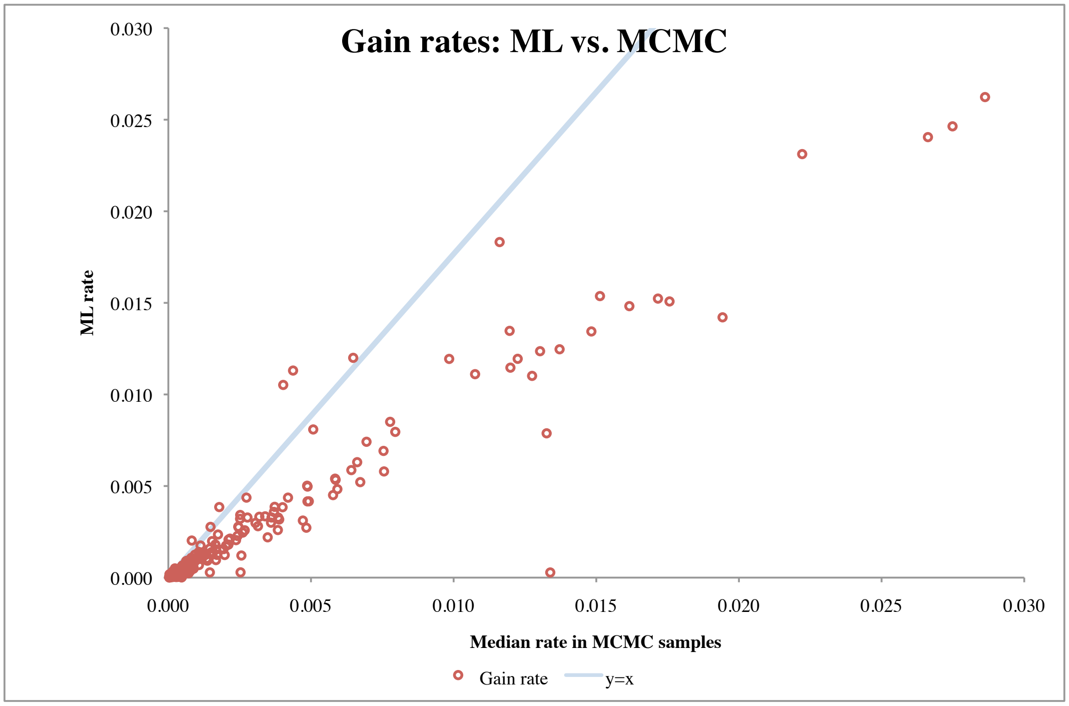
Gain rates: ML vs. MCMC
ML rate
Median rate in MCMC samples
0.000
0.005
0.010
0.015
0.020
0.025
0.030
Gain rate
y=x

7 Simulations

The consistency of different inference methods was assessed by generating 100 random simulated data sets using the median model inferred from the MCMC sampling (same sampling settings as before) on the main data set ("**Same model**"). In particular, we generated 7200 random intron absence-presence profiles, and performed ancestral reconstruction using Dollo parsimony, 100 independently run MCMC samples, and the maximum likelihood model found using sampling. The reconstrauction was compared to the known ancestral intron counts.

In order to assess the methods' robustness, we performed 100 additional simulations using a "**Mixed model**,' in which model parameters were multiplied by randomly drawn random variables. At each intron site, the root presence probability (π), as well as the site-specific rate multipliers (γ_j, ν_j) were multiplied by random lognormal variables $\ln \mathcal{N}(0, \sigma^2)$ with $\sigma = 0.2$ for π, $\sigma = 0.1$ for the gain rate multiplier ν_j, and $\sigma = 0.3$ for the loss rate multiplier γ_j. Concomitantly, the edge length parameters were also multiplied independently (among themselves, and across sites) by exponentially distributed random variables with mean 1.

In a final set of experiments, we assessed inference robustness with respect to **missing orthologs**. Using 100 simulated sets, we replaced randomly chosen entries with ambiguity. Namely, we split the sites into blocks of 34 (average number of sites per gene in the main data set), and erased presence-absence information independently within each block by 13% probability.

We chose the number of simulated intron-bearing sites in each set of experiments to yield similar number of informative entries as the main data set (containing 14% ambiguous entries), generating fewer sites without ambiguity (7200 sites for "Same model" and "Mixed model"), and to 9000 sites with "Missing orthologs" (since some sites without unambiguous intron presence are filtered out in the inference).

Figure 13 Estimation error in simulated data at some key nodes. Each point on the plots shows the actual number of ancestral introns along the X axis, and the estimation error (actual number subtracted from inferred number) along the Y axis. The 25% error level is shown by the slanted dotted lines. The MCMC and ML procedures have very small empirical bias, but Dollo parsimony is typically completely off target. ▶

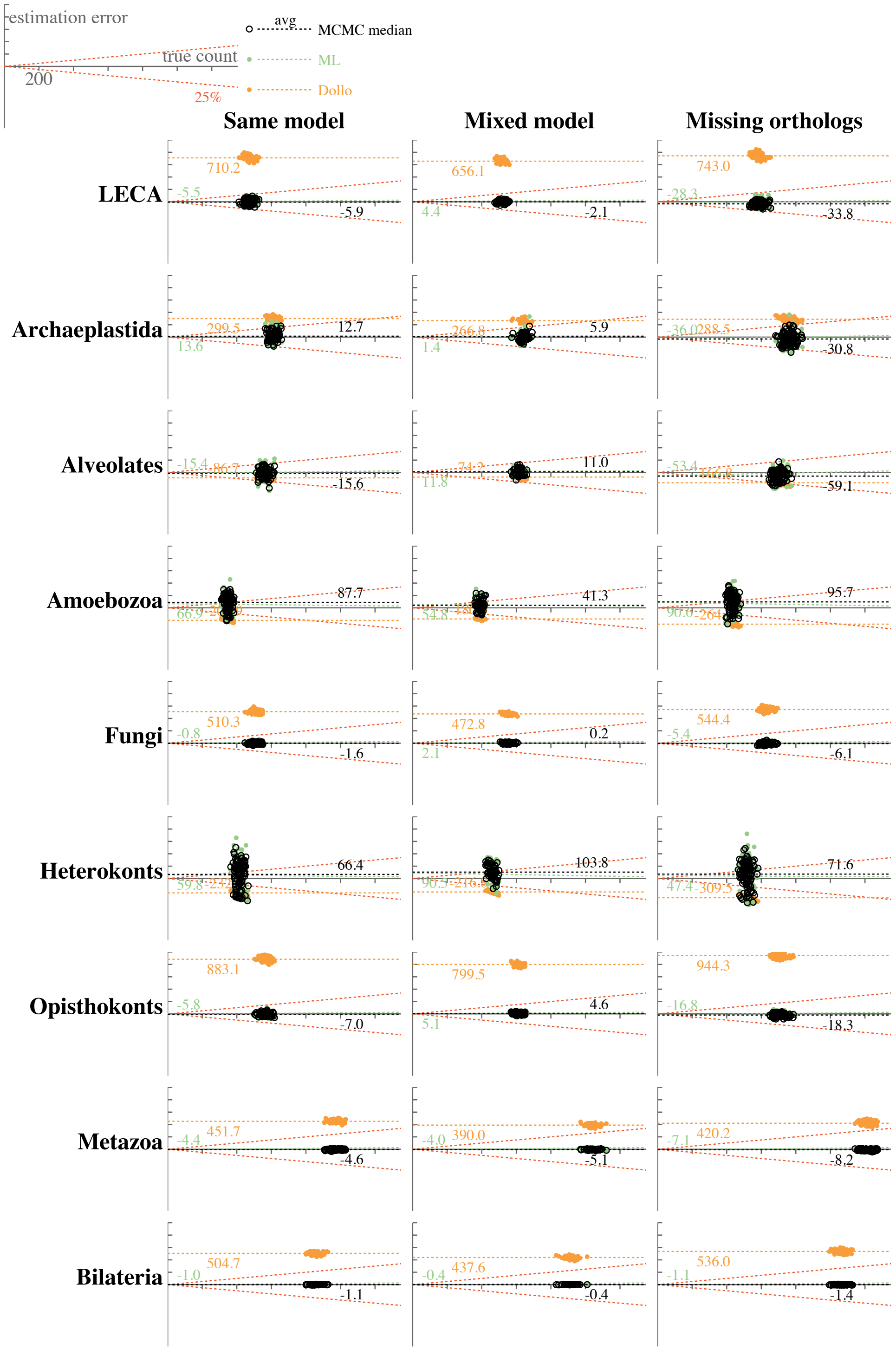

estimation error
true count
200
25%
avg
MCMC median
ML
Dollo
Same model
Mixed model
Missing orthologs
LECA
710.2
-5.5
-5.9
656.1
4.4
-2.1
743.0
-28.3
-33.8
Archaeplastida
299.5
13.6
12.7
266.8
1.4
5.9
-36.0
288.5
-30.8
Alveolates
15.4
-15.6
11.0
11.8
53.4
-59.1
Amoebozoa
87.7
66.9
41.3
54.8
95.7
90.0
Fungi
510.3
-0.8
-1.6
472.8
2.1
0.2
544.4
-5.4
-6.1
Heterokonts
66.4
59.8
103.8
71.6
47.4
309.5
Opisthokonts
883.1
-5.8
-7.0
799.5
5.1
4.6
944.3
-16.8
-18.3
Metazoa
451.7
-4.4
-4.6
390.0
-4.0
-5.1
420.2
-7.1
-8.2
Bilateria
504.7
-1.0
-1.1
437.6
-0.4
-0.4
536.0
-1.1
-1.4

8 Nægleria gruberi

We identified 68 homolog groups represented in the *Nægleria gruberi* genome (Fritz-Laylin *et al.*, 2010), using bi-directional best BLAST hits in the same way as for the main data set (§1.2). For groups with multiple Nægleria paralogs, we selected the one with the highest BLAST score. The 68 selected Nægleria genes were aligned to the multiple alignment profile constructed for their respective homolog groups (without recomputing the alignment between the 99 eukaryotes of the original data set). The alignments scored aligned intron positions alongside protein sequences, as described before (Csűrös *et al.*, 2007). The intron presence-intron table was computed from the alignments in the same way as for the main data set (§1.4). The resulting table comprises 4812 intron sites with 141 ambiguous Nægleria entries.

The data set contains 97 aligned Nægleria introns, from which 57 fall into a homologous position with at least one other eukaryote. Twenty-three of those are inferred to have been absent at the trifurcating LECA (average posterior presence probability 9%), and 34 are inferred to have been present (average probability 95%). At the chromalveolate and unikont ancestors, the numbers are the same. The shared Nægleria intron counts do not differ significantly across deep-branching unikonts (Capsaspora, Dictyostelium, Allomyces), either. Two introns are inferred to have been inserted in parallel between Ngru and the Metazoan ancestor. At the common ancestor of plants and green algæ, only 26 Nægleria introns are inferred to have been present.

Table 14 Nægleria introns projected onto the data set. The table lists the number of introns shared between Naegleria and other eukaryotic taxa. The table rows list the intron-sharing patterns between the Ngru and other taxa t ("Taxon" column). "Shared" sites have introns in both taxa, "Ngru-specific" sites have introns in Nægleria, opposing absent and ambiguous entries in t. "taxon-specific" sites have introns in t but not in Ngru. Notice that the counts include only unambiguosly aligned sites, and, thus, they may not sum up to as expected. For instance, only 88 of the 97 Ngru introns can be unambiguously projected onto homologous Dictyostelium sequences, and 25 of those correspond to a splice site in at least one Dictyostelium genome, the remaining 63 are "Ngru-specific." ▶

Nægleria introns in shared positions with other taxa

**Taxon**	introns	shared	Ngru-specific	taxon-specific
Ngru *Naegleria gruberi*	97			
99 eukaryotes	4772	57	40 (41%)	4574 (96%)
Dictyostelium	127	25	63 (65%)	97 (76%)
Metazoa	1767	40	57 (59%)	1676 (95%)
Hsap [*Homo sapiens*]	530	32	64 (66%)	485 (92%)
Cowc [*Capsaspora owczarzaki*]	217	25	64 (66%)	186 (86%)
Choanoflagellates Mbre+Prsp	567	25	68 (70%)	528 (93%)
Amac [*Allomyces macrogynus*]	132	24	65 (67%)	104 (79%)
Chytridiomycota Bden+Spun	378	28	66 (68%)	343 (91%)
Fungi	1454	41	56 (58%)	1375 (95%)
Unikonts	3160	51	46 (47%)	3013 (95%)
Alveolates	555	24	69 (71%)	517 (93%)
Chromalveolates	997	31	66 (68%)	935 (94%)
Atha [*Arabidopsis thaliana*]	460	25	59 (61%)	426 (93%)
Embryophyta [land plants]	543	27	69 (71%)	505 (93%)
Viridiplantae	1483	30	67 (69%)	1420 (96%)

References

Csűrös, M., Rogozin, I. B., and Koonin, E. V. (2008). Extremely intron-rich genes in the alveolate ancestors inferred with a flexible maximum likelihood approach. *Molecular Biology and Evolution*, **25**(5), 903–911.

Csűrös, M. (2008). Malin: maximum likelihood analysis of intron evolution in eukaryotes. *Bioinformatics*, **24**(13), 1538–1539.

Csűrös, M., Holey, J. A., and Rogozin, I. B. (2007). In search of lost introns. *Bioinformatics*, **23**(13), i87–i96.

Edgar, R. C. (2004). MUSCLE: multiple sequence alignment with high accuracy and high throughput. *Nucleic Acids Research*, **32**(5), 1792–1797.

Fritz-Laylin, L. K. *et al.* (2010). The genome of Naegleria gruberi illuminates early eukaryotic versatility. *Cell*, **140**, 631–642.

Guindon, S. and Gascuel, O. (2003). A simple, fast, and aaccurate accurate algorithm to estimate large phylogenies by maximum likelihood. *Systematic Biology*, **52**(5), 696–704.

Muller, J., Szklarczyk, D., Julien, P., Letunic, I., Roth, A., Kuhn, M., Powell, S., von Mering, C., Doerks, T., Jensen, L. J., and Bork, P. (2010). eggNOG v2.0: extending the evolutionary genealogy of genes with enhanced non-supervised orthologous groups, species and functional annotations. *Nucleic Acids Research*, **38**, D190–195.

Newton, M. E. and Raftery, A. E. (1994). Approximate Bayesian inference with the weighted likelihood bootstrap. *Journal of the Royal Statistical Society Series B*, **56**(1), 3–48.

Rogozin, I. B., Wolf, Y. I., Sorokin, A. V., Mirkin, B. G., and Koonin, E. V. (2003). Remarkable interkingdom conservation of intron positions and massive, lineage-specific intron loss and gain in eukaryotic evolution. *Current Biology*, **13**, 1512–1517.

Rogozin, I. B., Sverdlov, A. V., Babenko, V. N., and Koonin, E. V. (2005). Analysis of evolution of exon-intron structure of eukaryotic genes. *Briefings in Bioinformatics*, **6**(2), 118–134.
